# Interactions of Antiretroviral Drugs with Food, Beverages, Dietary Supplements, and Alcohol: A Systematic Review and Meta-analyses

**DOI:** 10.1007/s10461-022-03880-6

**Published:** 2022-11-01

**Authors:** Agnieszka Wiesner, Magdalena Skrońska, Gabriela Gawlik, Monika Marcinkowska, Paweł Zagrodzki, Paweł Paśko

**Affiliations:** 1 Department of Food Chemistry and Nutrition, Faculty of Pharmacy, Jagiellonian University Medical College, 9 Medyczna Str., 30-688 Kraków, Poland; 2 Department of Community and Public Health, Idaho State University, 1311 E Central Dr, Meridian, ID 83642 USA; 3 Department of Medicinal Chemistry, Faculty of Pharmacy, Jagiellonian University Medical College, 9 Medyczna Str., 30-688 Kraków, Poland

**Keywords:** Antiretroviral, Interaction, Food, Juice, Alcohol, Antirretroviral, Interacción, Comida, Jugo, Alcohol

## Abstract

Multiple factors may affect combined antiretroviral therapy (cART). We investigated the impact of food, beverages, dietary supplements, and alcohol on the pharmacokinetic and pharmacodynamic parameters of 33 antiretroviral drugs. Systematic review in adherence to PRISMA guidelines was performed, with 109 reports of 120 studies included. For each drug, meta-analyses or qualitative analyses were conducted. We have found clinically significant interactions with food for more than half of antiretroviral agents. The following drugs should be taken with or immediately after the meal: tenofovir disoproxil, etravirine, rilpivirine, dolutegravir, elvitegravir, atazanavir, darunavir, lopinavir, nelfinavir, ritonavir, saquinavir. Didanosine, zalcitabine, zidovudine, efavirenz, amprenavir, fosamprenavir, and indinavir should be taken on an empty stomach for maximum patient benefit. Antiretroviral agents not mentioned above can be administered regardless of food. There is insufficient evidence available to make recommendations about consuming juice or alcohol with antiretroviral drugs. Resolving drug-food interactions may contribute to maximized cART effectiveness and safety.

**Supplementary Information:**

The online version contains supplementary material available at 10.1007/s10461-022-03880-6.

## Resumen

Múltiples factores pueden afectar la terapia antirretroviral combinada (cART). Investigamos el impacto de los alimentos, las bebidas, los suplementos dietéticos y el alcohol en los parámetros farmacocinéticos y farmacodinámicos de 33 medicamentos antirretrovirales. Se realizó la revisión sistemática en apego a las guías PRISMA, con 109 reportes de 120 estudios incluidos. Para cada fármaco se realizaron metanálisis o análisis cualitativos. Hemos encontrado interacciones clínicamente significativas con alimentos para más de la mitad de los fármacos antirretrovirales. Los siguientes medicamentos deben tomarse durante o inmediatamente después de comer: tenofovir, disoproxil, etravirina, rilpivirine, dolutegravir, elvitegravir, atazanavir, darunavir, lopinavir, nelfinavir, ritonavir, saquinavir. Didanosina, zalcitabina, zidovudina, efavirenz, amprenavir, fosamprenavir e indinavir deben tomarse con el estómago vacío para obtener el máximo beneficio para el paciente. Los fármacos antirretrovirales no mencionados anteriormente se pueden administrar independientemente de los alimentos. No hay suficiente evidencia disponible para hacer recomendaciones sobre el consumo de jugo o alcohol con medicamentos antirretrovirales. Resolver las interacciones entre medicamentos y alimentos puede contribuir a maximizar la eficacia y la seguridad de cART.

## Introduction

Introducing combined antiretroviral therapy (cART) to the management of human immunodeficiency virus (HIV) infection not only did significantly reduce morbidity and mortality rates but also improved the quality of patients’ life [1]. However, multiple factors may alter the effectiveness and safety of antiretroviral treatment, e.g., potential cumulative toxicity, suboptimal patients’ adherence, drug-induced resistance, autoinduction, and inter-individual or inter-ethnical variability in drug response [2].

cART does not eradicate HIV, hence lifelong antiretroviral therapy is necessary. Newer classes of antiretroviral agents are better tolerated by patients with temporary gastrointestinal discomfort and fatigue as the most commonly reported side effects [3].

Nevertheless, chronic HIV treatment and aging may both contribute to the greater risk of metabolic disorders (e.g. dyslipidemia, insulin resistance, diabetes mellitus), cardiovascular diseases, hepatotoxicity, and renal impairment in people living with HIV (PLWH) [3–5]. Both temporary and prolonged adverse effects may entail therapy discontinuation and patients’ poor adherence [6].

The causes of poor adherence to cART are diverse: starting from the individual (such as forgetting, depression, alcohol misuse), through treatment-related (e.g. the complexity of dosing regimen, side effects), ending with health-service barriers (such as poor patient-physician relationship, distance to the clinic) [6]. Prolonged suboptimal adherence to the cART may result in disease progression, a higher plasma viral load (that implies the increased risk of HIV transmission), and the development of drug-resistant HIV strains [6, 7].

There are to main types of HIV drug resistance: pretreatment or acquired. Pretreatment HIV drug resistance occurs approximately in 10 percent of PLWH receiving initial treatment and its prevalence is the highest in low- and middle-income countries [8]. Acquired HIV drug resistance develops during the virus replication in the systemic presence of antiretroviral medications. According to the World Health Organisation (WHO) global report from 2019, the levels of resistance to commonly used nucleoside reverse-transcriptase inhibitors (NRTIs) and non-nucleoside reverse transcriptase inhibitors (NNRTIs) may range from 21 to 91% and from 50 to 97%, respectively [8]. Medications like dolutegravir, lopinavir, or darunavir are characterized by higher genetic barriers to drug resistance [8, 9].

Even in non-cART-resistant patients with high adherence levels, therapy can be ineffective, e.g. due to the autoinduction process. Several antiretroviral drugs, e.g. efavirenz, nevirapine, or nelfinavir may induce their own metabolism, leading to subtherapeutic plasma concentrations and unsatisfying treatment outcomes [10, 11].

Individual and ethnic global population variability settles the antiretroviral drug response as well. There are both qualitative and quantitative differences in genetic variants of drug-metabolizing enzymes (especially cytochrome P450) between racial and ethnic groups [12]. Genetic polymorphisms determine levels of expression, activity, and stability of enzymes, and thus impact the drug metabolism rate.

Considering all the above-mentioned factors, cART optimization is critical for achieving effective virologic response and a satisfactory safety profile. In our previous publications, it was scientifically proved that the dosing regimen and drug-food interactions can either positively or negatively influence the treatment with several groups of drugs [13–15]. So far, two systematic reviews have been published addressing the topic of antiretroviral drug-food interactions. The first, by de Souza et al. covered studies to 2012, and only 11 studies were included [16]. Since then, many new antiretroviral drugs have been registered and novel formulations of already registered drugs have been developed. The second systematic review, by Siritientong et al., is up-to-date, however, in this review, very general keywords were used, making the number of included studies relatively small [17]. In addition, the effect of food was investigated either for all antiretroviral drugs simultaneously or within pharmacological groups, and not for individual drugs. Hence, there is a need for an up-to-date, detailed, comprehensive systematic review that considers the effect of food on particular antiretroviral drugs.

In this systematic review, we assessed the potential impact of food, beverages, dietary supplements, and alcohol on the pharmacokinetic and pharmacodynamic parameters of antiretroviral drugs. We decided to examine all antiretroviral drugs that were on the global market at the time the review was prepared or have been registered in the past. We included 33 drugs in our analysis. The main aim of this study was to identify the clinically significant drug-food interactions and to propose practical guidelines on how to take antiretroviral agents in relation to food.

## Biopharmaceutical Characteristics of Antiretroviral Drugs

Even within the same pharmacological group, antiretroviral drugs may differ considerably in their chemical structure and physicochemical properties. In Table 1 we present biopharmaceutical characteristics of investigated antiretroviral drugs, namely the Biopharmaceutical Classification System (BCS) class, predicted logarithm of the partition coefficient (log P), solubility in water, and formulations available on the worldwide market (as of the date 10.10.2021). The data was obtained from http://www.drugbank.ca and Micromedex database).

**Table 1:** Biopharmaceutical characteristics of antiretroviral drugs (prepared based on data available from http://www.drugbank.ca and Micromedex database)

Drug	BCS class	Log P	Solubility in water (mg/mL)	Available oral formulations
Nucleoside reverse-transcriptase inhibitors (NRTIs)				
Abacavir	3	0.61	1.21	Tablet, solution
Apricitabine	3	− 1.1	3.41	Capsule
Didanosine	3	− 0.99	6.58	Tablet, delayed-release capsule, capsule with enteric-coated beads, powder to prepare the oral solution
Emtricitabine	1	− 0.8	2	Tablet, capsule, oral solution
Lamivudine	3	− 1.3	2.76	Tablet, capsule, oral solution
Stavudine	1	− 0.73	40.5	Capsule, oral solution, powder to prepare the oral solution
Tenofovir disoproxil	3	2.65	0.712	Tablet, granule, oral powder
Tenofovir alafenamide	3	1.49	0.236	Tablet
Zalcitabine	3	− 1.3	7.05	Tablet
Zidovudine	3	− 0.1	16.3	Capsule, tablet, oral solution, syrup
Non-nucleoside reverse transcriptase inhibitors (NNRTIs)				
Delavirdine	Not specified	2.77	0.086	Tablet
Doravirine	2	3.47	0.0115	Tablet
Efavirenz	2	3.89	0.00855	Tablet, capsule, oral solution
Etravirine	4	3.67	0.0169	Tablet
Nevirapine	2	1.75	0.105	Tablet, modified-release tablet, oral suspension
Rilpivirine	2	3.8	0.016	Tablet
Integrase strand transfer inhibitors (INSTIs)				
Dolutegravir	2	2.2	0.0922	Tablet, tablet to prepare oral suspension
Elvitegravir	2	3.66	0.00652	Tablet
Raltegravir	3	− 0.39	53.9	Tablet, chewable tablet, granules to prepare oral suspension
Bictegravir	2	1.28	0.0537	Tablet
Cabotegravir	2	0.76	0.113	Tablet
Protease inhibitors (PIs)				
Amprenavir	2	2.43	0.0491	Capsule
Atazanavir	2	4.54	4.5	Capsule, oral powder
Darunavir	2	1.8	0.0668	Tablet, oral suspension
Fosamprenavir	2	1.92	0.685	Tablet, oral suspension
Indinavir	1	2.9	0.015	Capsule
Lopinavir	4	4.69	0.00192	Capsule, tablet, oral solution
Nelfinavir	2	4.72	0.00191	Tablet, oral powder
Ritonavir	4	4.24	0.00126	Capsule, tablet, oral solution, oral suspension
Saquinavir	4	3.96	0.00765	Capsule, tablet
Tipranavir	4	6.29	0.000205	Capsule, liquid capsule, oral solution
Fusion inhibitors				
Maraviroc	3	4.79	0.0106	Tablet, oral solution
Fostemsavir	Not specified	0.64	0.431	Extended-release tablet

### Biopharmaceutical Classification System (BCS)

The Biopharmaceutical Classification System (BCS) divides drugs into 4 classes, based on their solubility in water and intestinal permeability:

- class 1 (high solubility and high permeability)—such compounds are well absorbed and less vulnerable to factors that may affect bioavailability; food usually has no significant impact on absorption,
- class 2 (low solubility and high permeability)—the bioavailability of those compounds is limited by their solvation rate; food usually has a positive impact on absorption (e.g. high-fat meal promotes dissolution of lipophilic drugs),
- class 3 (high solubility and low permeability)—the drug dissolves fast but the absorption is limited by the permeation rate; food may negatively affect absorption by altering the process of drug dissolution,
- class 4 (low solubility and low permeability)—those compounds have poor bioavailability; it is hard to predict the impact of food [18].

### Logarithm of the Partition Coefficient (log P)

Partition coefficient (P) is the ratio of concentrations of an un-ionized compound in a mixture of two immiscible solvents (one is lipophilic, e.g. octanol, and the second hydrophilic, e.g. water). Log P value describes drug lipophilicity—the higher log P, the more lipophilic drug is:

- log P < 1—hydrophilic drug, absorption can be lower in the presence of food rich in fat,
- log P between 1 and 3—a drug with moderate lipophilicity,
- log P > 3—lipophilic drug, food rich in fat may have a positive impact on drug absorption [19].

The abovementioned biopharmaceutical characteristics explains the rationale for considering the interactions with food for individual antiretroviral drugs, rather than within the pharmacological group.

## Methods

This systematic review was conducted in adherence to the Preferred Reporting Items for Systematic Reviews and Meta-analyses (PRISMA) statement. The protocol was not prepared and not registered.

### Information Sources and Search Strategy

In July 2022, the search in two databases was performed: Medline (via PubMed) and Embase, covering reports from the date of database inception to the date of the search. Additional records that can be classified as grey literature were identified via a Google Scholar search. Further reports were found by checking the product characteristics of antiretroviral drugs registered on the global market, as well as the reference lists of previously identified scientific publications.

During the searching process, the following keywords and phrases were applied: antiretroviral drugs names in combination with “food”, “food-drug interaction”, “meal”, “diet”, “breakfast”, “dietary supplement”, “alcohol”, and “juice”. When possible, MeSH terms and Emtree terms were used. In Medline (via Pubmed) and Embase, the keyword search was restricted to titles and abstracts, while in Google Scholar to titles only. The detailed searching strategy is provided in Supplementary Material S1.

### Eligibility Criteria

All articles describing or investigating the impact of food, beverages, dietary supplements, and alcohol on pharmacokinetic and/or pharmacodynamic parameters of orally taken antiretroviral drugs were considered for inclusion in this systematic review. The pharmacokinetic parameters of interest were primarily AUC—area under the plasma drug concentration–time curve that reflects the extent of exposure to a drug, C_max_—the maximum (or peak) serum drug concentration, and t_max_—the time to reach C_max_ that both relate to the rate of drug absorption. Pharmacodynamic indicators of drug efficacy were mainly plasma HIV-RNA count, mean CD4 gain, and frequency of virological failure. To present possibly the most complete and reliable evidence, no restrictions were applied regarding study type, study year, the number of participants, or their characteristics (e.g. age, gender, race). Both studies involving healthy volunteers and HIV (+) patients were considered. We excluded review studies, in vitro studies, and studies performed on animals.

### Selection Process

The selection process was carried out using the Rayyan software. The authors, namely AW and PP, independently screened titles and abstracts of each record and selected those eligible for inclusion in the systematic review. Any disagreements between authors were discussed among the remaining authors and resolved by consensus.

### Data Collection Process

From included studies, the authors, namely AW and MS, independently extracted available data of study type, the number of participants and their characteristics (health state, gender, race), antiretroviral drug dose and formulation, quantitative food composition (caloric load, percentage or weight amount of fat, carbohydrates, and protein), qualitative meal composition, alcohol concentration (in studies of interactions with alcohol), pre- and postprandial values of pharmacokinetic parameters (AUC, C_max_, t_max_), pharmacodynamic parameters, and possible mechanism of interaction between antiretroviral drug and food. Additionally, statistically and/or clinically significant percentage changes in pharmacokinetic parameters were collected (if given) or calculated (if not provided by study authors). The data collection process was supervised by PP, who resolved any discrepancies.

### Study Risk of Bias Assessment

Quality assessment of each included study was performed independently by two authors, namely AW and PP. Depending on the study design, different tools were used, such as version 2 of the Cochrane risk-of-bias tool for parallel trials (RoB 2) [20], Cochrane risk-of-bias tool for crossover studies [21], National Institutes of Health (NIH) quality assessment tool for observational cohort, and cross-sectional studies [22], and NIH quality assessment tool for before-after (pre-post) studies [22]. Any discrepancies in the assessment were discussed between the authors (AW and PP) and a consensus was made.

### Data Synthesis

Quantitative analyses were performed for each drug if 2 or more food-effect studies with specified and comparable study designs were available, e.g. randomized and non-randomized studies were not synthesized in the same meta-analysis, as well as parallel and cross-over studies. The effect measures were mean differences (fed vs. fasted) of the three main outcomes: AUC, C_max_, and t_max_. If values of pharmacokinetic parameters were presented as geometric means with confidence intervals or the coefficient of variation, they were converted to arithmetic means and standard deviations using the method designed by Higgins et al. [23]. When median values and range or interquartile range were reported, the approach proposed by Wan et al. [24] was used to estimate arithmetic mean and standard deviation. For AUC, the adopted unit was ug·h/mL, for C_max_—ug/mL, and for t_max_—h. Results reported in other units have been transformed accordingly.

Meta-analyses were conducted in the Review Manager (RevMan) [Computer program] Version 5.4.1, The Cochrane Collaboration, 2020. As the heterogeneity of studies was predicted to be high, the random effects model with the inverse variance method was used for the calculation of study weights. The results of meta-analyses were visually displayed as forest plots. To identify and measure the heterogeneity of studies included in the meta-analyses, the I^2^ statistics and Chi^2^ tests were calculated. I^2^ < 25% together with the P-value from the Chi^2^ test < 0.1 indicated the low heterogeneity, 25% < I^2^ < 75%—the moderate heterogeneity, whereas I^2^ > 75% and P > 0.1—the high heterogeneity [25]. In cases of moderate or high heterogeneity, subgroup analyses were performed. Grouping variables were: the type of meal, drug formulation, the health state of the participants, or study risk of bias. The grouping variables differed between the meta-analyses, depending on the characteristics of the included studies. Due to the small number of studies available, we chose to conduct a subgroup analysis if at least two studies were included in each subgroup. Additionally, to assess the robustness of the synthesized results, sensitivity analyses were conducted by changing the analysis model. Since none of the meta-analyses included 10 or more studies, funnel plots were not generated.

For drugs, for which meta-analyses could not be performed due to the unknown/variable study designs or lack of pharmacokinetic data, the results of available studies were summarized and discussed.

## Results

### Eligible Studies

During an extensive databases search, 7814 records were identified in total: 3438 in Medline (via Pubmed) and 4376 in Embase. 4084 duplicate records were removed using the automation tool (Rayyan), and the other 1478 were deleted manually. Titles and abstracts of remained 2252 papers were meticulously screened. 2158 studies did not address the research question or met the exclusion criteria. Of 94 studies that were assessed for eligibility, 7 were excluded for reasons as follows: drug other than antiretroviral being assessed [26–28], a study design that does not allow to resolve the impact of food on an antiretroviral drug [29, 30], a study performed on hepatitis B patients [31], the impact of food being assessed for intravenously given antiretroviral drug [32].

Additional 349 records were identified during a search in other information sources such as Google Scholar (322), product characteristics (16), reference lists (4), and conference reports (8). 41 records were sought for retrieval, and 15 were not retrieved since we did not find either an abstract or full text available. Of 26 reports assessed for eligibility, 4 were found to be duplicates after reading the full text [33–36], and 23 remaining reports were included.

**Fig. 1:**
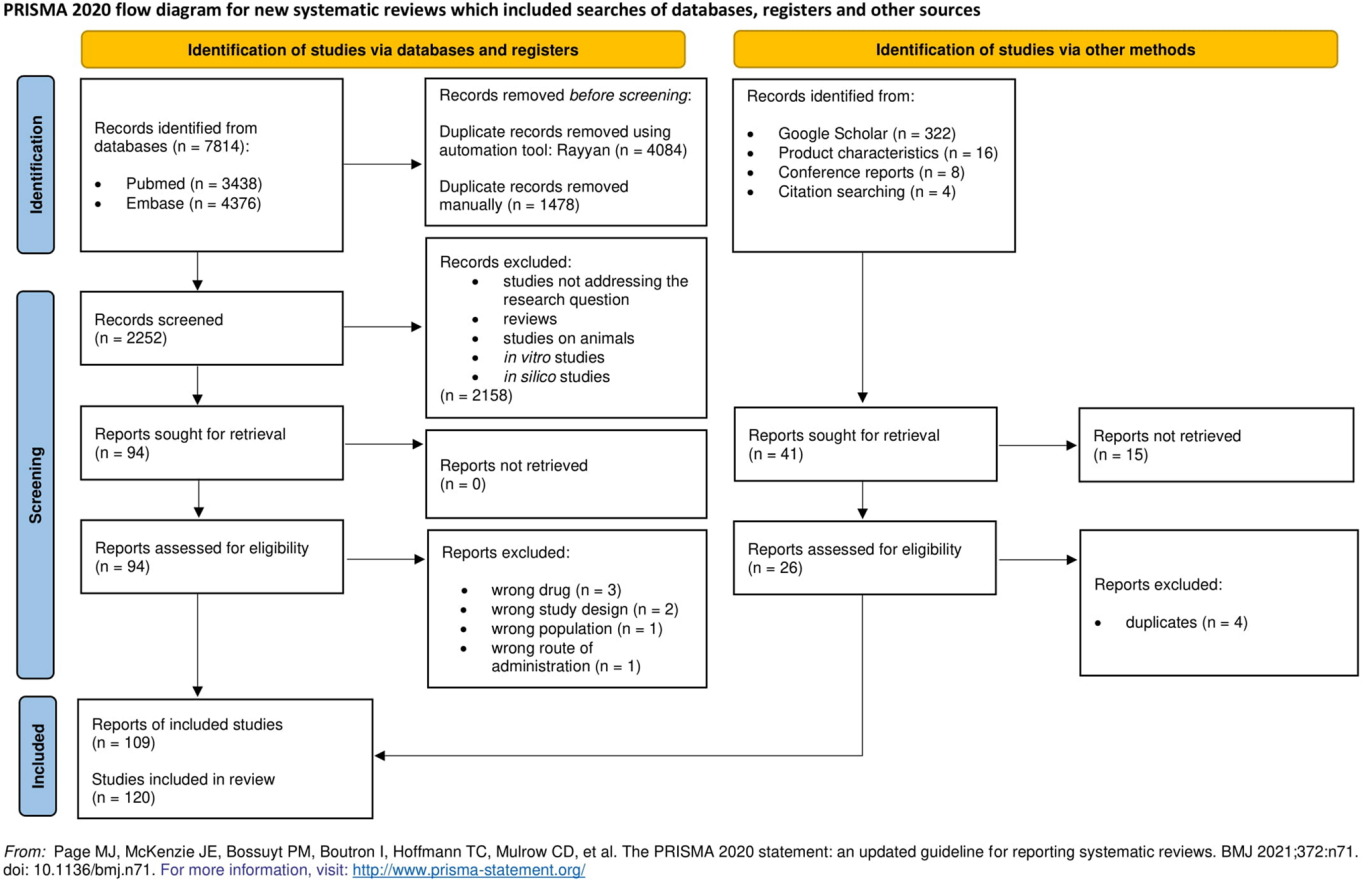
Flow diagram of the search strategy

Ultimately, in our systematic review, we included 109 reports of 120 studies. The flowchart of the search strategy is presented in Fig. 1.

### Study Characteristics

The majority of included studies were open-label, cross-over clinical trials, as this study design is recommended by FDA for assessing the food effect [37]. The list of studies included in the systematic review is presented in Table 2. Detailed study characteristics are available in Supplementary Material S2, in which studies were organized by pharmacological groups and pooled for each antiretroviral drug. Hence, studies in which the effect of food on the pharmacokinetic/pharmacodynamic parameters of several antiretroviral drugs was assessed, appeared more than once, with results collected for each drug. Additionally, studies investigating the impact of alcohol and juices were presented separately.

**Table 2:** List of studies included in the systematic review

StudyID	References	Investigated drugs	Randomized?	Study design	Source	Participants health state	Number of participants
Aarnoutse2003	[38]	Indinavir, ritonavir	Randomized clinical trial	Open-label, cross-over	Article	HIV (+)	9
Aarnoutse2003_2	[39]	Nelfinavir	Randomized clinical trial	Open-label, parallel	Article	Healthy	27
Anderson2014	[40]	Doravirine	Randomized clinical trial	Double-blind, parallel	Article	Healthy	48
Angel1993	[41]	Lamivudine	Randomized clinical trial	Open-label, cross-over	Article	HIV (+)	12
Behm2017-1	[42]	Lamivudine, doravirine, tenofovir disoproxil	Randomized clinical trial	Open-label, cross-over	Article	Healthy	14
Behm2017-2	[42]	Lamivudine, doravirine, tenofovir disoproxil	Randomized clinical trial	Open-label, cross-over	Article	Healthy	14
Brainard2011	[43]	Raltegravir	Randomized clinical trial	Open-label, cross-over	Article	Healthy	20
Brouwers2007	[44]	Amprenavir	Non-randomized clinical trial	Open-label, cross-over	Article	Healthy	5
Carver1999	[45]	Indinavir	Randomized clinical trial	Open-label, cross-over	Article	HIV (+)	9
Chittick1999	[46]	Abacavir	Randomized clinical trial	Open-label, cross-over	Article	HIV (+)	18
Cloarec2017	[47]	Darunavir	Not applicable	Case studies	Article	HIV (+)	2
Crauwels2013	[48]	Rilpivirine	Randomized clinical trial	Open-label, cross-over	Article	Healthy	20
Crauwels2016	[49]	Rilpivirine	Randomized clinical trial	Open-label, cross-over	Conference	Healthy	32
Crauwels2019	[50]	Emtricitabine, darunavir, tenofovir alafenamide	Randomized clinical trial	Open-label, cross-over	Article	Healthy	24
Custodio2013	[51]	Emtricitabine, rilpivirine, tenofovir disoproxil	Randomized clinical trial	Open-label, cross-over	Article	Healthy	24
Custodio2015-1	[52]	Tenofovir alafenamide	Randomized clinical trial	Open-label, cross-over	Conference	Healthy	39
Custodio2015-2	[52]	Tenofovir alafenamide	Randomized clinical trial	Open-label, cross-over	Conference	Healthy	42
Damle2002-1	[53]	Didanosine	Randomized clinical trial	Open-label, cross-over	Article	Healthy	20
Damle2002-2	[53]	Didanosine	Randomized clinical trial	Open-label, cross-over	Article	Healthy	25
Damle2002-3	[53]	Didanosine	Randomized clinical trial	Open-label, cross-over	Article	Healthy	29
Demarles2002	[54]	Amprenavir	Randomized clinical trial	Open-label, cross-over	Article	Healthy	12
Dumitrescu2020	[55]	Lamivudine, dolutegravir	Randomized clinical trial	Open-label, cross-over	Article	Healthy	16
Falcos2002	[56]	Amprenavir	Randomized clinical trial	Open-label, cross-over	Article	Healthy	24
Fätkenheuer2005-1	[57]	Maraviroc	Randomized clinical trial	Parallel, placebo-controlled	Article	HIV (+)	16
Fätkenheuer2005-2	[57]	Maraviroc	Randomized clinical trial	Parallel, placebo-controlled	Article	HIV (+)	
Gallicano2003	[58]	Ritonavir	Randomized clinical trial	Open-label, cross-over	Article	Healthy	10
Gruber2013	[59]	Maraviroc	Randomized clinical trial	Double-blind, parallel	Article	Healthy	10
Han2014	[60]	Emtricitabine	Randomized clinical trial	Open-label, parallel	Article	Healthy	60
Hernandez2008	[61]	Didanosine	Randomized clinical trial	Open-label, parallel	Article	HIV (+)	21
Holdich2008	[62]	Apricitabine	Randomized clinical trial	Open-label, cross-over	Article	Healthy	12
Hugen2002	[63]	Saquinavir	Non-randomized clinical trial	Longitudinal, uncontrolled	Article	HIV (+)	6
Jiang2013	[64]	Tenofovir disoproxil	No data	No data	Article	Healthy	12
Kaeser2005	[65]	Nelfinavir	Randomized clinical trial	Open-label, cross-over	Article	Healthy	12
Kakuda2014	[66]	Darunavir, ritonavir	Randomized clinical trial	Open-label, cross-over	Article	Healthy	17
Kakuda2014_2-1	[67]	Darunavir, ritonavir	Randomized clinical trial	Open-label, cross-over	Article	Healthy	32
Kakuda2014_2-2	[67]	Darunavir, ritonavir	Randomized clinical trial	Open-label, cross-over	Article	Healthy	128
Kakuda2014_3	[68]	Darunavir	Randomized clinical trial	Open-label, cross-over	Article	Healthy	19
Kanter2010	[69]	Lopinavir, ritonavir	Randomized clinical trial	Open-label, cross-over	Article	Healthy	12
Kaul1998	[70]	Stavudine	Randomized clinical trial	Open-label, cross-over	Article	HIV (+)	17
Kaul2010	[71]	Efavirenz	Randomized clinical trial	Open-label, cross-over	Article	Healthy	24
Kearney2005	[72]	Didanosine, tenofovir disoproxil	Non-randomized clinical trial	Open-label, cross-over	Article	Healthy	28
Kenyon1998	[73]	Saquinavir	Randomized clinical trial	Open-label, cross-over	Article	Healthy	8
Klein2007-1	[74]	Lopinavir, ritonavir	Randomized clinical trial	Open-label, cross-over	Article	Healthy	63
Klein2007-2	[74]	Lopinavir, ritonavir	Randomized clinical trial	Open-label, cross-over	Article	Healthy	48
Klein2007-3	[74]	Lopinavir, ritonavir	Randomized clinical trial	Open-label, cross-over	Article	Healthy	15
Knupp1993	[75]	Didanosine	Randomized clinical trial	Open-label, cross-over	Article	HIV (+)	10
Krishna2018	[76]	Raltegravir	Randomized clinical trial	Open-label, cross-over	Article	Healthy	17
Kupferschmidt2003	[77]	Saquinavir	Non-randomized clinical trial	Open-label, cross-over	Article	Healthy	8
Kurowski2002	[78]	Nelfinavir	Non-randomized clinical trial	Open-label, cross-over	Article	Healthy	24
Lamorde2012	[79]	Emtricitabine, efavirenz, tenofovir disoproxil	Non-randomized clinical trial	Open-label, cross-over	Article	HIV (+)	15
Lamorde2012_2	[80]	Lopinavir, ritonavir	Non-randomized clinical trial	Open-label, cross-over	Article	HIV (+)	12
Lamorde2015	[81]	Rilpivirine	Non-randomized clinical trial	Open-label, longitudinal	Article	HIV (+)	15
Li2021	[82]	Tenofovir alafenamide	Randomized clinical trial	Open-label, cross-over	Article	Healthy	73
Li2021_2	[83]	Tenofovir alafenamide	Randomized clinical trial	Open-label, cross-over	Article	Healthy	67
Li2021_3	[84]	Tenofovir alafenamide	Randomized clinical trial	Open-label, parallel	Article	Healthy	64
Lopez2006	[85]	Didanosine	Not applicable	Retrospective, cohort	Article	HIV (+)	668
Lotterer1991	[86]	Zidovudine	Randomized clinical trial	Open-label, cross-over	Article	HIV (+)	13
Lu2012	[87]	Tenofovir dipivoxil	Randomized clinical trial	Open-label, cross-over	Article	Healthy	12
Majeed2020	[88]	Bictegravir, emtricitabine, tenofovir alafenamide	Randomized clinical trial	Open-label, cross-over	Conference	Healthy	48
Marier2006	[89]	Abacavir	Randomized clinical trial	Open-label, cross-over	Article	Healthy	80
Mathias2018	[90]	Bictegravir	Non-randomized clinical trial	Open-label, parallel	Conference	Healthy	42
McCance-Katz2013	[91]	Efavirenz, ritonavir	Randomized clinical trial	Double-blind, parallel	Article	HIV (+)	10
McDowell2000	[92]	Abacavir	Randomized clinical trial	Open-label, cross-over	Article	HIV (+)	25
Mehta2020	[93]	Rilpivirine, dolutegravir	Randomized clinical trial	Open-label, cross-over	Article	healthy	24
Moore1999	[94]	Lamivudine, zidovudine	Randomized clinical trial	Open-label, cross-over	Article	healthy	24
Morse2003	[95]	Delavirdine	Randomized clinical trial	Open-label, cross-over	Article	HIV (+)	13
Nazareno1995	[96]	Zalcitabine	Randomized clinical trial	Open-label, cross-over	Article	HIV (+)	20
Ng2008	[97]	Ritonavir	Randomized clinical trial	Open-label, cross-over	Article	Healthy	25
None	[98]	Amprenavir	No data	No data	SmPC^a^	Healthy	12
None	[99]	Atazanavir	No data	No data	SmPC	Healthy	12
None	[100]	Efavirenz	No data	No data	SmPC	Healthy	12
None	[101]	Emtricitabine, bictegravir, tenofovir alafenamide	No data	No data	SmPC	Healthy	12
None	[102]	Fosamprenavir	No data	No data	SmPC	Healthy	12
None	[103]	Fostemsavir	No data	No data	SmPC	Healthy	12
None	[104]	Lamivudine	No data	No data	SmPC	Healthy	12
None	[105]	Lopinavir	No data	No data	SmPC	Healthy	12
None	[106]	Maraviroc	No data	No data	SmPC	Healthy	12
None	[107]	Nelfinavir	No data	No data	SmPC	Healthy	22
None	[108]	Nevirapine	No data	No data	SmPC	Healthy	24
None	[109]	Ritonavir	No data	No data	SmPC	Healthy	12
None	[110]	Saquinavir	No data	No data	SmPC	Healthy	6
None	[111]	Tenofovir disoproxil	No data	No data	SmPC	Healthy	12
None	[112]	Tipranavir	No data	No data	SmPC	Healthy	12
Oki2004	[113]	Lopinavir, ritonavir	Non-randomized clinical trial	Open-label, cross-over	Article	Healthy	8
Patel2018	[114]	Cabotegravir	Randomized clinical trial	Open-label, parallel	Unpublished study	Healthy	15
Patel2018_2	[115]	Cabotegravir	Randomized clinical trial	Open-label, cross-over	Unpublished study	Healthy	22
Patel2019	[116]	Cabotegravir	Randomized clinical trial	Open-label, cross-over	Article	Healthy	24
Penzak2002	[117]	Indinavir	Non-randomized clinical trial	Open-label, cross-over	Article	Healthy	13
Piscitelli2002	[118]	Saquinavir	Non-randomized clinical trial	Longitudinal, uncontrolled	Article	Healthy	10
Rhee2014	[119]	Raltegravir	Randomized clinical trial	Open-label, cross-over	Article	Healthy	12
Ruhnke1993	[120]	Zidovudine	Randomized clinical trial	Open-label, parallel	Article	HIV (+)	27
Saah2001	[121]	Indinavir, ritonavir	Randomized clinical trial	Double-blind, parallel	Article	Healthy	53
Sadler1999	[122]	Amprenavir	Randomized clinical trial	Open-label, cross-over	Article	HIV (+)	18
Sahai1992	[123]	Zidovudine	Randomized clinical trial	Open-label, cross-over	Article	HIV (+)	11
Salem2015-1	[124]	Ritonavir	Randomized clinical trial	Open-label, cross-over	Article	Healthy	48
Salem2015-2	[124]	Ritonavir	Randomized clinical trial	Open-label, cross-over	Article	Healthy	24
Sanchez2007	[125]	Didanosine	Not applicable	Prospective, cohort	Article	HIV (+)	103
Scholler2008	[126]	Etravirine	Randomized clinical trial	Open-label, cross-over	Article	Healthy	24
Sekar2007	[127]	Darunavir, ritonavir	Randomized clinical trial	Open-label, cross-over	Article	Healthy	24
Sevinsky2015	[128]	Atazanavir	Randomized clinical trial	Open-label, cross-over	Article	Healthy	64
Shelton1994	[129]	Zidovudine	Randomized clinical trial	Open-label, cross-over	Article	HIV (+)	18
Shelton2001	[130]	Indinavir	Randomized clinical trial	Open-label, cross-over	Article	HIV (+)	14
Shelton2003	[131]	Delavirdine	Randomized clinical trial	Open-label, cross-over	Article	HIV (+)	21
Shiomi2014	[132]	Emtricitabine, elvitegravir, tenofovir disoproxil	Randomized clinical trial	Open-label, cross-over	Article	Healthy	11
Shyu1991	[133]	Didanosine	Non-randomized clinical trial	Open-label, cross-over	Article	HIV (+)	8
Song2011	[134]	Dolutegravir	Randomized clinical trial	Open-label, cross-over	Article	Healthy	24
Song2015	[135]	Dolutegravir	Randomized clinical trial	Open-label, cross-over	Article	Healthy	12
Stevens2000	[136]	Didanosine	Randomized clinical trial	Longitudinal, uncontrolled	Article	HIV (+)	77
Unadkat1990	[137]	Zidovudine	Randomized clinical trial	Open-label, cross-over	Article	HIV (+)	6
Veldkamp2001	[138]	Saquinavir, ritonavir	Randomized clinical trial	Open-label, cross-over	Article	HIV (+)	6
Wang1995	[139]	Emtricitabine	No data	No data	Conference	HIV (+)	12
Weller2014	[140]	Abacavir, lamivudine, dolutegravir	Randomized clinical trial	Open-label, cross-over	Article	Healthy	12
Wenning2007	[141]	Raltegravir	Randomized clinical trial	Open-label, cross-over	Conference	Healthy	20
Yamada2018	[142]	Emtricitabine, elvitegravir, tenofovir alafenamide	Randomized clinical trial	Open-label, cross-over	Article	Healthy	12
Yee2020-1	[143]	Lamivudine, doravirine, tenofovir disoproxil	Randomized clinical trial	Open-label, cross-over	Article	Healthy	24
Yee2020-2	[143]	Lamivudine, doravirine, tenofovir disoproxil	Randomized clinical trial	Open-label, cross-over	Article	Healthy	24
Yeh1998-1	[144]	Indinavir	Randomized clinical trial	Open-label, cross-over	Article	Healthy	12
Yeh1998-2	[144]	Indinavir	Randomized clinical trial	Double-blind, placebo-controlled	Article	Healthy	8
Yonemura2018	[145]	Elvitegravir	Randomized clinical trial	Open-label, cross-over	Article	Healthy	12
Yuen2001	[146]	Abacavir	Randomized clinical trial	Open-label, cross-over	Article	Healthy	24

^a^Summary of product characteristics

### Risk of Bias Assessment

The risk of bias assessments carried out by AW and PP were generally consistent. For 12 studies, the assessment differed in one domain, however, the final assessments for all studies were the same. 32 studies (26%) were judged as having a high risk of bias, and the remaining were of moderate quality (at least one domain with some concerns). A detailed risk of bias assessment is presented in Supplementary Material S3.

### Quantitative Syntheses

For 16 antiretroviral drugs, quantitative syntheses were performed. Studies excluded from meta-analyses (with reasons) are listed in Supplementary material S4. Overall, 50 meta-analyses were conducted. In Table 3, the results of meta-analyses for individual antiretroviral drugs are presented. Forest plots of each meta-analysis are available in Supplementary material S5.

**Table 3:** Results of meta-analyses performed for individual antiretroviral drugs

	Drug	Outcome	Study designs	Number of studies	Number of participants	Mean difference [95% CI]	Test for overall effect	Interpretation of result	I^2^ (%)	P-value for Chi^2^ test	Judgment of heterogeneity	Subgroup analysis?
NRTIs	Didanosine	AUC_inf_	Randomized, cross-over	2	82	− 0.92 [− 1.19, − 0.64]	Z = 6.52 (P < 0.00001)	Higher AUC in fasted	0	0.49	Low	No
		AUC_inf_	Non-randomized, cross-over	2	36	− 1.19 [− 2.18, − 0.19]	Z = 2.34 (P = 0.02)	Slightly higher AUC in fasted	62	0.11	Moderate	No
		C_max_	Randomized, cross-over	2	82	− 0.51 [− 0.72, − 0.30]	Z = 4.84 (P < 0.00001)	Higher C_max_ in fasted	63	0.03	Moderate	Yes
		C_max_	Non-randomized, cross-over	2	36	− 0.85 [− 1.99, 0.30]	Z = 1.44 (P = 0.15)	No significant difference between C_max_ in fasted and fed states	86	0.007	High	No
		t_max_	Non-randomized, cross-over	2	35	0.96 [− 0.88, 2.80]	Z = 1.02 (P = 0.31)	No significant difference between t_max_ in fasted and fed states	95	< 0.00001	High	No
	Emtricitabine	AUC_inf_	Randomized, cross-over	4	47	− 0.65 [− 1.26, − 0.03]	Z = 2.07 (P = 0.04)	Slightly higher AUC in fasted	36	0.15	Low	No
		C_max_	Randomized, cross-over	4	47	0.17 [− 0.21, 0.55]	Z = 0.89 (P = 0.37)	No significant difference between C_max_ in fasted and fed states	0	0.99	Low	No
		t_max_	Randomized, cross-over	4	47	0.50 [0.32, 0.67]	Z = 5.54 (P < 0.00001)	Higher t_max_ in fed	28	0.22	Low	No
	Lamivudine	AUC_inf_	Randomized, cross-over	6	114	− 1.28 [− 2.04, − 0.51]	Z = 3.25 (P = 0.001)	Higher AUC in fasted	87	< 0.00001	High	Yes
		C_max_	Randomized, cross-over	6	114	− 0.47 [− 0.64, − 0.30]	Z = 5.51 (P < 0.00001)	Higher C_max_ in fasted	53	0.02	Moderate	Yes
		t_max_	Randomized, cross-over	6	114	0.84 [0.35, 1.33]	Z = 3.37 (P = 0.0007)	Higher t_max_ in fed	94	< 0.00001	High	Yes
	Tenofovir disoproxil	AUC_inf_	Randomized, cross-over	4	73	0.68 [0.52, 0.85]	Z = 7.92 (P < 0.00001)	Higher AUC in fed	26	0.21	Low	No
		C_max_	Randomized, cross-over	4	73	0.07 [0.04, 0.10]	Z = 4.67 (P < 0.00001)	Higher C_max_ in fed	36	0.13	Low	No
		t_max_	Randomized, cross-over	4	73	0.43 [0.14, 0.71]	Z = 2.94 (P = 0.003)	Higher t_max_ in fed	90	< 0.00001	High	Yes
	Tenofovir alafenamide	AUC_inf_	Randomized, cross-over	6	230	0.07 [0.04, 0.10]	Z = 4.11 (P < 0.0001)	Higher AUC in fed	93	< 0.00001	High	Yes
		C_max_	Randomized, cross-over	6	230	− 0.03 [− 0.07, 0.00]	Z = 1.70 (P = 0.09)	No significant difference between C_max_ in fasted and fed states	87	< 0.00001	High	Yes
		t_max_	Randomized, cross-over	4	149	0.60 [0.51, 0.68]	Z = 13.67 (P < 0.00001)	Higher t_max_ in fed	29	0.21	Low	No
	Zidovudine	AUC_inf_	Randomized, cross-over	4	72	− 0.17 [− 0.38, 0.04]	Z = 1.62 (P = 0.11)	No significant difference between AUC in fasted and fed states	4	0.37	Low	No
		C_max_	Randomized, cross-over	6	96	− 0.44 [− 0.79, − 0.09]	Z = 2.48 (P = 0.01)	Slightly higher C_max_ in fasted	85	< 0.00001	High	Yes
		t_max_	Randomized, cross-over	5	85	0.88 [0.63, 1.14]	Z = 6.74 (P < 0.00001)	Higher t_max_ in fed	48	0.1	Moderate	Yes
NNRTIs	Doravirine	AUC_inf_	Randomized, cross-over	2	38	1.78 [0.11, 3.44]	Z = 2.09 (P = 0.04)	Slightly higher AUC in fed	18	0.29	Low	No
		C_max_	Randomized, cross-over	2	38	0.13 [− 0.07, 0.33]	Z = 1.29 (P = 0.20)	No significant difference between C_max_ in fasted and fed states	92	< 0.00001	High	Yes
		t_max_	Randomized, cross-over	2	38	0.18 [− 0.60, 0.97]	Z = 0.46 (P = 0.65)	No significant difference between t_max_ in fasted and fed states	82	< 0.0001	High	Yes
	Rilpivirine	AUC	Randomized, cross-over	3	55	1.00 [0.15, 1.85]	Z = 2.31 (P = 0.02)	Slightly higher AUC in fed	75	0.0006	High	Yes
		C_max_	Randomized, cross-over	3	55	0.04 [0.02, 0.07]	Z = 3.18 (P = 0.001)	Higher C_max_ in fed	77	0.0002	High	Yes
		t_max_	Randomized, cross-over	3	55	0.15 [− 0.16, 0.46]	Z = 0.96 (P = 0.34)	No significant difference between t_max_ in fasted and fed states	0	0.79	Low	No
INSTIs	Dolutegravir	AUC_inf_	Randomized, cross-over	2	30	24.68 [19.04, 30.33]	Z = 8.58 (P < 0.00001)	Higher AUC in fed	5	0.38	Low	No
		C_max_	Randomized, cross-over	2	30	1.39 [1.14, 1.63]	Z = 10.93 (P < 0.00001)	Higher C_max_ in fed	0	0.55	Low	No
		t_max_	Randomized, cross-over	2	30	1.36 [0.84, 1.87]	Z = 5.16 (P < 0.00001)	Higher t_max_ in fed	47	0.11	Moderate	No
	Elvitegravir	AUC_inf_	Randomized, cross-over	2	23	15.39 [12.74, 18.05]	Z = 11.36 (P < 0.00001)	Higher AUC in fed	0	0.85	Low	No
		C_max_	Randomized, cross-over	2	23	1.41 [1.20, 1.61]	Z = 13.65 (P < 0.00001)	Higher C_max_ in fed	0	0.57	Low	No
		t_max_	Randomized, cross-over	2	23	− 0.30 [− 0.58, − 0.02]	Z = 2.10 (P = 0.04)	Slightly higher t_max_ in fasted	13	0.33	Low	No
	Raltegravir	AUC_inf_	Randomized, cross-over	2	37	− 5.74 [− 14.10, 2.63]	Z = 1.34 (P = 0.18)	No significant difference between AUC in fasted and fed states	85	< 0.0001	High	Yes
		C_max_	Randomized, cross-over	3	56	− 1.98 [− 3.57, − 0.38]	Z = 2.43 (P = 0.02)	Slightly higher C_max_ in fasted	82	< 0.00001	High	No*
		t_max_	Randomized, cross-over	2	36	1.09 [0.18, 1.99]	Z = 2.36 (P = 0.02)	Slightly higher t_max_ in fasted	65	0.009	High	No*
PIs	Amprenavir	AUC_inf_	Randomized, cross-over	2	42	− 3.06 [− 6.45, 0.32]	Z = 1.78 (P = 0.08)	No significant difference between AUC in fasted and fed states	29	0.24	Low	No
		C_max_	Randomized, cross-over	2	42	− 2.66 [− 5.03, − 0.29]	Z = 2.20 (P = 0.03)	Slightly higher C_max_ in fasted	84	0.01	High	No*
		t_max_	Randomized, cross-over	2	42	1.04 [0.70, 1.38]	Z = 6.02 (P < 0.00001)	Higher t_max_ in fed	46	0.18	Moderate	No*
	Darunavir	AUC_inf_	Randomized, cross-over	6	235	26.28 [19.83, 32.73]	Z = 7.99 (P < 0.00001)	Higher AUC in fed	56	0.007	Moderate	No*
		C_max_	Randomized, cross-over	6	235	2.38 [1.78, 2.98]	Z = 7.78 (P < 0.00001)	Higher C_max_ in fed	89	< 0.00001	High	No*
		t_max_	Randomized, cross-over	6	235	1.03 [0.82, 1.24]	Z = 9.68 (P < 0.00001)	Higher t_max_ in fed	37	0.09	Moderate	No*
	Indinavir	AUC_inf_	Randomized, cross-over	2	18	− 3.97 [− 6.95, − 0.99]	Z = 2.61 (P = 0.009)	Higher AUC in fasted	55	0.07	Moderate	Yes
		C_max_	Randomized, cross-over	3	27	− 3.25 [− 4.85, − 1.66]	Z = 4.00 (P < 0.0001)	Higher C_max_ in fasted	64	0.02	Moderate	Yes
		t_max_	Randomized, cross-over	3	27	1.28 [0.60, 1.96]	Z = 3.68 (P = 0.0002)	Higher t_max_ in fed	90	< 0.00001	High	Yes
	Lopinavir	AUC_inf_	Randomized, cross-over	2	24	27.91 [15.55, 40.28]	Z = 4.43 (P < 0.00001)	Higher AUC in fed	0	0.64	Low	No
		C_max_	Randomized, cross-over	2	24	1.68 [0.83, 2.53]	Z = 3.86 (P = 0.0001)	Higher C_max_ in fed	0	0.54	Low	No
		t_max_	Randomized, cross-over	2	24	1.89 [1.27, 2.52]	Z = 5.93 (P < 0.00001)	Higher t_max_ in fed	0	0.43	Low	No
	Ritonavir	AUC_inf_	Randomized, cross-over	5	153	− 0.94 [− 1.51, − 0.36]	Z = 3.21 (P = 0.001)	Higher AUC in fasted	18	0.29	Low	No
		C_max_	Randomized, cross-over	5	153	− 0.18 [− 0.32, − 0.03]	Z = 2.41 (P = 0.02)	Slightly higher C_max_ in fasted	91	< 0.00001	High	Yes
		t_max_	Randomized, cross-over	6	162	1.50 [1.31, 1.70]	Z = 15.19 (P < 0.00001)	Higher t_max_ in fed	7	0.38	Low	No

*subgroup analysis not applicable-less than two studies in each subgroup

#### Subgroup Analyses

In 29 (58%) of meta-analyses, moderate or high heterogeneity was revealed, and for those, subgroup analyses were conducted (when possible). In Table 4, we present the results of only those subgroup analyses where the grouping variables potentially explain the heterogeneity of the studies included in meta-analyses. Forest plots of all performed subgroup analyses are available in Supplementary material S5.

**Table 4:** Results of subgroup analyses for meta-analyses with moderate or high heterogeneity

Drug	Outcome	Overall	Subgroup analysis	Test for subgroup differences	Interpretation
Lamivudine	AUC_inf_	n = 114, MD [95% CI] = − 1.28 [− 2.04, − 0.51], I^2^ = 87%, P < 0.00001	Grouping variable—*drug formulation* Tablets: n = 28, MD [95% CI] = − 0.14 [− 0.42, 0.13], I^2^ = 0%, P = 0.5 Tablets (combined): n = 62, MD [95% CI] = − 0.33 [− 0.81, 0.16], I^2^ = 0%, P = 0.55 Coated granules: n = 24, MD [95% CI] = − 2.01 [− 2.81, − 1.21], I^2^ = 0%, P = 0.82 Uncoated granules: n = 24, MD [95% CI] = − 3.05 [− 3.80, − 2.30], I^2^ = 0%, P = 0.89	I^2^ = 95.3%, P < 0.00001	Heterogeneity can be explained by different drug formulations For tablets (single or combined)—no significant difference in AUC between fasted and fed states For coated and uncoated granules—significantly lower AUC in a fed state
	t_max_	n = 114, MD [95% CI] = 0.84 [0.35, 1.33], I^2^ = 94%, P < 0.00001	Grouping variable—*drug formulation* Tablets: n = 28, MD [95% CI] = 2.09 [1.67, 2.51], I^2^ = 0%, P = 0.64 Tablets (combined): n = 62, MD [95% CI] = 1.05 [0.79, 1.31], I^2^ = 0%, P = 0.63 Coated granules: n = 24, MD [95% CI] = 0.03 [− 0.19, 0.24], I^2^ = 0%, P = 0.89 Uncoated granules: n = 24, MD [95% CI] = − 0.22 [− 0.40, − 0.05], I^2^ = 0%, P = 0.37	I^2^ = 97.9%, P < 0.00001	Heterogeneity can be explained by different drug formulations For tablets (single or combined)—significantly higher t_max_ in a fed state For coated and uncoated granules—no significant difference in t_max_ between fasted and fed states
Tenofovir disoproxil	t_max_	n = 73, MD [95% CI] = 0.43 [0.14, 0.71], I^2^ = 90%, P < 0.00001	Grouping variable—*drug formulation* Tablets (combined): n = 49, MD [95% CI] = 0.79 [0.50, 1.09], I^2^ = 69%, P = 0.01 Coated granules: n = 24, MD [95% CI] = 0.01 [− 0.14, 0.15], I^2^ = 0%, P = 0.95 Uncoated granules: n = 24, MD [95% CI] = 0.02 [− 0.14, 0.17], I^2^ = 0%, P = 0.77	I^2^ = 91.7%, P < 0.00001	Heterogeneity can be partially explained by different drug formulations For tablets combined—significantly higher t_max_ in a fed state For coated and uncoated granules—no significant difference in t_max_ between fasted and fed states
Tenofovir alafenamide	AUC_inf_	n = 230, MD [95% CI] = 0.07 [0.04, 0.10], I^2^ = 93%, P < 0.00001	Grouping variable—*drug formulation* Tablets: n = 152, MD [95% CI] = 0.11 [0.09, 0.13], I^2^ = 57%, P = 0.05 Tablets (combined): n = 78, MD [95% CI] = 0.02 [0.01, 0.04], I^2^ = 0%, P = 0.59	I^2^ = 98.2%, P < 0.00001	Heterogeneity can be partially explained by different drug formulations For tablets combined—lower food effect on AUC than for tablets
Doravirine	C_max_	n = 38, MD [95% CI] = 0.13 [− 0.07, 0.33], I^2^ = 92%, P < 0.00001	Grouping variable—*type of meal* High-fat: n = 14, MD [95% CI] = − 0.03 [− 0.16, 0.09], I^2^ = 0%, P = 0.47 Pudding: n = 24, MD [95% CI] = − 0.02 [− 0.13, 0.08], I^2^ = 21%, P = 0.26 Apple sauce: n = 24, MD [95% CI] = 0.41 [0.33, 0.50], I^2^ = 0%, P = 0.41	I^2^ = 96.5%, P < 0.00001	Heterogeneity can be explained by different types of meals For high-fat meal and pudding—no significant difference in C_max_ between fasted and fed states For apple sauce—significantly higher C_max_ in a fed state
	t_max_	n = 38, MD [95% CI] = 0.18 [− 0.60, 0.97], I2 = 82%, P < 0.0001	Grouping variable—*drug formulation* Tablets (single or combined): n = 14, MD [95% CI] = 1.93 [0.95, 2.91], I^2^ = 0%, P = 0.92 Coated granules: n = 24, MD [95% CI] = − 0.08 [− 0.57, 0.42], I^2^ = 0%, P = 0.94 Uncoated granules: n = 24, MD [95% CI] = − 0.82 [− 1.35, − 0.29], I^2^ = 29%, P = 0.24	I^2^ = 91.5%, P < 0.00001	Heterogeneity can be explained by different drug formulations For tablets (single or combined)—significantly higher t_max_ in a fed state For coated granules—no significant difference in t_max_ between fasted and fed states For uncoated granules—significantly lower t_max_ in a fed state
Raltegravir	AUC_inf_	n = 37, MD [95% CI] = − 5.74 [− 14.10, 2.63], I^2^ = 85%, P < 0.0001	Grouping variable—*type of meal* High-fat: n = 37, MD [95% CI] = 0.96 [− 2.05, 3.97], I^2^ = 0%, P = 0.81 Low-fat: n = 17, MD [95% CI] = − 15.33 [− 20.80, − 9.86], I^2^ = 0%, P = 0.54	I^2^ = 96.2%, P < 0.00001	Heterogeneity can be explained by different types of meals For high-fat meals—no significant difference in AUC between fasted and fed states For low-fat meals—significantly lower C_max_ in a fed state
Indinavir	AUC_inf_	n = 18, MD [95% CI] = − 3.97 [− 6.95, − 0.99], I^2^ = 55%, P = 0.07	Grouping variables—*type of meal and health state* Low-fat and light meals, healthy: n = 11, MD [95% CI] = − 0.87 [− 3.93, 2.20], I^2^ = 0%, P = 0.86 Other meals, HIV (+): n = 7, MD [95% CI] = − 6.31 [− 8.93, − 3.68], I^2^ = 0%, P = 0.4	I^2^ = 85.7%, P = 0.008	Heterogeneity can be explained either by the type of meal or health state For low-fat and light meals and in healthy participants—no significant difference in AUC between fasted and fed states For other meals and HIV (+) patients—significantly lower AUC in a fed state
	C_max_	n = 27, MD [95% CI] = − 3.25 [− 4.85, − 1.66], I^2^ = 64%, P = 0.02	Grouping variables—*type of meal and health state* Low-fat and light meals, healthy: n = 11, MD [95% CI] = − 1.53 [− 2.75, − 0.32], I^2^ = 0%, P = 0.98 Other meals, HIV (+): n = 16, MD [95% CI] = − 4.69 [− 5.99, − 3.40], I^2^ = 0%, P = 0.67	I^2^ = 91.8%, P = 0.0005	Heterogeneity can be explained either by the type of meal or health state For low-fat and light meals and in healthy participants—a lower mean difference in C_max_ between fasted and fed states

#### Sensitivity Analyses

After changing the statistical model from the random effects model to the fixed effects model, no significant qualitative differences in overall effect were found for 45 of 50 meta-analyses, however, the fixed effects model generally produced higher values of overall effect and narrower confidence intervals of a mean difference than the random effects model. For the remaining 5 meta-analyses, differences between random and fixed effects models are listed in Table 5. Forest plots of these meta-analyses are provided in Supplementary material S5.

**Table 5:** Significant qualitative differences in the results of meta-analyses after changing statistical model from random to fixed effects model

Drug	Outcome	Random effects model	Interpretation of result	Fixed effects model	Interpretation of result
Didanosine	C_max_	Mean difference [95% CI] = − 0.85 [− 1.99, 0.30], Z = 1.44 (P = 0.15)	No significant difference between C_max_ in fasted and fed states	Mean difference [95% CI] = − 0.45 [− 0.72, − 0.18], Z = 3.30 (P = 0.0010)	Higher C_max_ in fasted
	t_max_	Mean difference [95% CI] = 0.96 [− 0.88, 2.80], Z = 1.02 (P = 0.31)	No significant difference between t_max_ in fasted and fed states	Mean difference [95% CI] = 1.34 [0.98, 1.70], Z = 7.36 (P < 0.00001)	Higher t_max_ in fed
Tenofovir alafenamide	C_max_	Mean difference [95% CI] = − 0.03 [− 0.07, 0.00], Z = 1.70 (P = 0.09)	No significant difference between C_max_ in fasted and fed states	Mean difference [95% CI] = − 0.06 [− 0.07, − 0.05], Z = 9.41 (P < 0.00001)	Higher C_max_ in fasted
Doravirine	C_max_	Mean difference [95% CI] = 0.13 [− 0.07, 0.33], Z = 1.29 (P = 0.20)	No significant difference between C_max_ in fasted and fed states	Mean difference [95% CI] = 0.17 [0.11, 0.22], Z = 5.97 (P < 0.00001)	Higher C_max_ in fed
Raltegravir	AUC	Mean difference [95% CI] = − 5.74 [− 14.10, 2.63], Z = 1.34 (P = 0.18)	No significant difference between C_max_ in fasted and fed states	Mean difference [95% CI] = − 2.83 [− 5.47, − 0.19], Z = 2.10 (P = 0.04)	Slightly higher AUC in fasted

### Qualitative Syntheses

For 17 antiretroviral drugs, conducting meta-analyses was not possible due to the insufficient number of studies eligible for quantitative synthesis. For those drugs, we summarized the available evidence in Table 6, whereas a detailed description of studies is provided in Supplementary material S2.

**Table 6:** The qualitative synthesis of evidence regarding the impact of food on antiretroviral drugs

Class	Drug	Outcome	Number of studies	Number of participants	Overall effect
NRTIs	Abacavir	AUC	4	134	No significant difference between fasted and fed states
		C_max_	4	134	↓ by 23–32% in a fed state
		t_max_	2	42	↑ by 0.5–1 h in a fed state
	Apricitabine	AUC	1	12	No significant difference between fasted and fed states
		C_max_	1	12	No significant difference between fasted and fed states
		t_max_	1	12	↑ by 1.3 h in a fed state
	Stavudine	AUC	1	17	No significant difference between fasted and fed states
		C_max_	1	17	↓ by 47% in a fed state
		t_max_	1	17	↑ by 1.1 h in a fed state
	Tenofovir dipivoxil	AUC	1	12	↑ by 23% in a fed state
		C_max_	1	12	No significant difference between fasted and fed states
		t_max_	1	12	↑ by 0.5 h in a fed state
	Zalcitabine	AUC	1	20	No significant difference between fasted and fed states
		C_max_	1	20	↓ by 39% in a fed state
		t_max_	1	20	↑ by 0.8 h in a fed state
NNRTIs	Delavirdine	AUC	1	13	No significant difference between fasted and fed states
		C_max_	1	13	↓ by 21% in a fed state
		t_max_	1	13	No significant difference between fasted and fed states
	Efavirenz	AUC	3	51	↑ by 13–28% in a fed state Light meals: no significant difference between fasted and fed states
		C_max_	3	51	↑ by 39–79% in a fed state Light meals: no significant difference between fasted and fed states
	Etravirine	AUC	1	24	↑ by 29–54% in a fed state High-fiber meals: no significant difference between fasted and fed states
		C_max_	1	24	↑ by 44–46% in a fed state High-fiber meals: no significant difference between fasted and fed states
		t_max_	1	24	↑ by 1–2 h
	Nevirapine	AUC	1	24	No significant difference between fasted and fed states
		C_max_	1	24	No significant difference between fasted and fed states
		t_max_	1	24	No significant difference between fasted and fed states
INSTIs	Bictegravir	AUC	2	64	No significant difference between fasted and fed states
		C_max_	2	64	No significant difference between fasted and fed states
	Cabotegravir	AUC	3	61	No significant difference between fasted and fed states
		C_max_	3	61	No significant difference between fasted and fed states
		t_max_			
PIs	Atazanavir	AUC	2	76	High-fat meals: from no significant difference to ↑ by 35% in fed state Light meals: ↑ by 28–70% in a fed state
		C_max_	2	76	High-fat meals: no significant difference between fasted and fed states Light meals: ↑ by 40–57% in a fed state
		t_max_	2	76	High-fat meals: ↑ by 1.5–2.5 h in a fed state Light meals: ↑ by 0.5 h in a fed state
	Fosamprenavir	AUC	2	17	↓ by 28–49% in a fed state
		C_max_	2	17	↓ by 46–50% in a fed state
		t_max_	2	17	↑ by 0.7–2 h in a fed state
	Nelfinavir	AUC	2	73	High- and medium-fat meals: ↑ by 510–520% in a fed state Low- and very low-fat meals: ↑ by 220–310% in a fed state
		C_max_	2	73	High- and medium-fat meals: ↑ by 330–380% in a fed state Low- and very low-fat meals: ↑ by 200–230% in a fed state
		t_max_	1	23	↑ by 1–2 h in a fed state
	Saquinavir	AUC	2	14	↑ by 571–625% in a fed state
		C_max_	1	8	↑ by 435% in a fed state
		t_max_	1	8	↑ by 3.25 h in a fed state
	Tipranavir	AUC	1	12	No significant difference between fasted and fed states
		C_max_	1	12	No significant difference between fasted and fed states
Fusion inhibitors	Maraviroc	AUC	3	28	↓ by 33–73% in a fed state
		C_max_	3	28	↓ by 33–60% in a fed state
		t_max_	2	16	No significant difference between fasted and fed states
	Fostemsavir	AUC	1	12	No significant difference between fasted and fed states
		C_max_	1	12	No significant difference between fasted and fed states

#### Interactions with Dietary Supplements

We found limited evidence for interactions between several INSTIs and mineral supplements. Dolutegravir and bictegravir AUC and C_max_ both decreased by 33–42% in the presence of calcium carbonate. Ferrous fumarate analogically caused a decrease of AUC and C_max_ by 55–58% for dolutegravir, and by 63–70% for bictegravir [90, 135]. Interestingly, when dolutegravir and bictegravir were given with both supplements and a moderate-fat meal, no significant changes in pharmacokinetic parameters occurred. Another solution proposed to avoid malabsorption was maintaining the 2 h break between dolutegravir or bictegravir and calcium or iron supplements intake [90, 135].

HIV-positive patients often use garlic supplements due to their immunomodulating and cholesterol-lowering effects. Garlic products may either induce or inhibit CYP enzymes and thus the potential risk of pharmacokinetic interaction with several protease inhibitors exists. Garlic supplements were found to interfere with saquinavir absorption by decreasing AUC and C_max_ by 51% and 54%, respectively [118]. However, individual concentration–time profiles revealed that in 3 patients AUC was slightly increased during garlic supplement consumption. Interestingly, after 10 days of the washing period, AUC values did not bounce back to the baseline level.

Regarding darunavir, we found case studies of 2 patients with substantial garlic consumption, in whom subtherapeutic C_through_ darunavir concentrations were revealed. After garlic eviction, darunavir concentrations normalized within 1 month [47].

After ritonavir co-intake with a garlic-containing dietary supplement, no significant changes in AUC and C_max_ occurred. However, these results cannot be extrapolated to steady-state conditions [58]. Moreover, regular garlic consumption during the treatment with ritonavir may exacerbate gastrointestinal adverse effects [58].

#### Interactions with Juices

Ingredients of grapefruit and Seville orange juices act as inhibitors of intestinal CYP enzymes (especially CYP3A4). Among antiretroviral drugs, protease inhibitors present the highest potential to interact with juices, due to being extensively metabolized by CYP enzymes.

Amprenavir acts both as a substrate and inhibitor of CYP3A4. Juice ingredients can reduce amprenavir first-pass metabolism by inhibiting intestinal CYP enzymes. Nevertheless, co-administration with 200 mL of grapefruit juice only slightly decreased amprenavir C_max_ (by 22%) and delayed t_max_ (by 0.4 h), without significantly affecting AUC [54]. These results indicate that the gut metabolism of amprenavir is low.

For indinavir, the mean AUC and C_max_ values remained unchanged after co-intake with grapefruit juice [117, 130]. However, in one study, individual changes in indinavir AUC ranged from a 25% decrease to even a 25% increase [117]. Such a high variability may negatively impact the treatment, by either causing its ineffectiveness or increasing the risk of adverse drug reactions.

After indinavir administration with Seville orange juice, no significant changes in AUC and C_max_ occurred [117]. However, t_max_ was slightly longer (by 0.6 h), since a high amount of carbohydrates (especially pectins) in juice may delay gastric emptying [117].

In a study of saquinavir, AUC and C_max_ significantly increased (by 50% and 93%, respectively) after co-intake with grapefruit juice [77]. By inhibiting intestinal CYP enzymes, juice ingredients reduced the extensive first-pass metabolism of saquinavir. However, that effect is variable and should not be considered as therapeutic guideline to improve low saquinavir bioavailability [77].

In patients with gastric hypoacidity, delavirdine absorption can be even 50% lower [131]. Co-intake with acidic beverages, such as orange juice, may improve delavirdine bioavailability. Regular orange juice ingredients do not inhibit CYP enzymes but may lower gastric acidity. In a single study, the elevations of delavirdine AUC and C_max_ were observed (by 57% and 53%, respectively) after co-administration with regular orange juice. Improved AUC and higher C_max_, however, were only visible in patients with gastric pH ≥ 3 [131].

#### Interactions with Alcohol

The pooled prevalence of alcohol use disorders among people living with HIV/AIDS is almost 30% [147]. Excessive alcohol consumption not only impairs a patient's immunity but also may cause liver damage that contributes to the disease progression [147]. There are concerns that alcohol may alter the effectiveness and safety of antiretroviral therapy due to the shared metabolism pathways with antiretroviral drugs. On the other hand, when the recommendation to avoid co-intake of alcohol and antiretroviral drugs is made, patients often miss doses of their medications if they plan on consuming alcohol. Such non-adherence can lead to the development of resistance to therapy.

We found only several studies addressing the issue of interactions between antiretroviral drugs and alcohol. Co-intake of maraviroc with ethanol did not produce any significant changes in maraviroc pharmacokinetics. Contrastingly, ethanol AUC increased slightly but significantly (by 12%), indicating that maraviroc can potentially enhance alcohol concentration and toxicity [59].

In a study of abacavir, a single dose did not significantly affect ethanol pharmacokinetics, however, abacavir AUC and t_1/2_ significantly increased (by 41% and 26%, respectively) [92]. Both abacavir and ethanol are metabolized by alcohol dehydrogenase and hence may compete in the metabolism phase [92].

After the co-intake of efavirenz and ritonavir with alcohol, no significant changes occurred in the pharmacokinetic parameters of these drugs [91]. However, in the presence of both efavirenz and ritonavir, ethanol blood concentration slightly decreased (AUC by 14% and C_max_ by 12%) [91].

## Discussion

### Findings and Interpretations

Results of our review indicate that food may have a diverse and significant impact on the pharmacokinetic and pharmacodynamic parameters of antiretroviral drugs. The overall high heterogeneity of investigated studies suggests that the basis of antiretroviral drug-food interactions is multifactorial. Based on quantitative and qualitative syntheses, we pointed out several factors that may influence the magnitude and clinical relevance of antiretroviral drug-food interactions.

#### Physicochemical Properties of a Drug

Physicochemical drug properties rather than belonging to the pharmacological group may explain the impact of food. The majority of NRTIs are hydrophilic drugs, with log P values < 1 (Table 1), which makes them highly soluble in water. Hydrophilic compounds dissolute slower in the presence of fat, and gastric emptying is prolonged after a meal as well. It may explain significantly higher postprandial values of t_max_ reported for emtricitabine, lamivudine, stavudine, zalcitabine, and zidovudine. On contrary, tenofovir disoproxil and alafenamide are lipophilic drugs (with log P values between 1 and 3), which may explain better absorption in the presence of meals rich in fat.

Our review indicates that food has a positive or neutral impact on the bioavailability of NNRTIs. Drugs from this group belong mostly to the 2nd BCS class (Table 1). They exert low solubility in water and are highly permeable through the intestinal membrane. Except for delavirdine and nevirapine, all NNRTIs are lipophilic, with log P > 3 (Table 1). Fat from food induces bile acids and pancreatic juice secretion and thus promotes the solubilization of lipophilic NNRTIs. High-fat meals delay gastric emptying so it takes longer for NNRTIs to dissolve and their absorption is better.

Food has shown a positive or neutral impact on INSTIs absorption. Most of INSTIs are members of the 2nd BCS class (Table 1) with low solubility in water and high intestinal permeability. Only raltegravir belongs to the 3rd BCS class, hence it is highly soluble and hardly permeable. INSTIs vary in terms of lipophilicity—from the most lipophilic elvitegravir (with log P > 3) to the most hydrophilic raltegravir (with log P < 0).

The influence of food differed among drugs that belong to the PIs group. PIs are heterogeneous in terms of BCS classification (class 1st, 2nd, and 4th), lipophilicity (log P from 1.8 to 6.29), and solubility in water (Table 1). For lipophilic drugs, such as atazanavir, lopinavir, nelfinavir, ritonavir, and saquinavir, our review has confirmed more efficient dissolution and absorption in the presence of food rich in fat.

#### Type of Meal

For several drugs, the impact of food on pharmacokinetics differed depending on the type of meal. Didanosine co-administration with a high-fat meal or a standard meal produced more distinct changes than with low-fat and light meals such as yogurt or apple [53, 72, 75]. It may be explained by delayed gastric emptying in the presence of food rich in fat. Furthermore, gastric juice secretion is increased after a meal, resulting in lower pH values. In an acidic environment, didanosine is unstable due to hydrolysis [53].

For rilpivirine, the bioavailability was improved in the presence of a high-fat meal, whereas the impact of a standard or moderate-fat meal differed from neutral to beneficial [48, 51, 81, 93]. However, no positive effects were observed after the co-administration of rilpivirine and the protein-rich cocktail. It can be explained by the slower tablet disintegration and drug liberation in the presence of nutritional drinks [48].

Also, raltegravir and nelfinavir absorption depended on the type of meal and improved with the increasing content of fat in food [39, 43, 76, 107, 148]. The opposite pattern was observed for atazanavir—light meals improved bioavailability, whereas high-fat meals exhibited a neutral effect [99, 128].

The impact of a meal type was revealed for indinavir as well. Due to its basic chemical character, indinavir is mostly absorbed in the upper small intestine. Meals rich in fat delay gastric emptying, neutralize acidic pH, and may cause indinavir precipitation. Consequently, less drug can reach the absorption site, and the overall bioavailability is lower [144]. A negative food impact was not observed for low-fat and low-calorie meals [38, 144].

#### Drug Formulation

The formulation of an antiretroviral drug could be another factor determining the onset and magnitude of interaction with food. For didanosine, in studies of enteric-coated capsules instead of tablets, less distinct postprandial changes in pharmacokinetic parameters occurred [53, 61]. Moreover, for enteric-coated capsules taken with or without food, similar antiretroviral activity was reported after 28 days [61]. The enteric-coated formulation may be preferable over tablets, as it protects didanosine from hydrolysis in the presence of gastric acid and improves its AUC as well [53].

Lamivudine, tenofovir disoproxil, and doravirine tablets (single or combined with other antiretroviral agents) were absorbed more slowly after the meal, whereas no significant postprandial changes in t_max_ were observed for coated and uncoated granules [42, 143]. Moreover, our findings suggest that ingesting lamivudine granules in the fasting state may slightly improve its bioavailability, while the impact of food on the AUC of lamivudine tablets remains neutral.

For tenofovir alafenamide and rilpivirine, the food effect was lower when both drugs were given with other antiretroviral agents as a combined tablet.

In the case of darunavir, a positive food influence on bioavailability was observed only for tablets. No significant deviations were reported when the oral suspension was administered with a standard meal, except for the delay of t_max_ [66]. For lopinavir tablets, contrastingly, the food effect was diminished, possibly due to the presence of hydrophilic excipients in a formulation [74].

For ritonavir, the bioavailability of an oral solution and capsules remained unchanged in the presence of meals rich in fat, whereas tablets and oral powder had slightly lower values of AUC and C_max_ when taken with a high-fat meal [67, 97, 109, 124].

#### Patient’s Age

Age-related differences in pharmacokinetics between children and adults may influence the food effect. For example, unlike in adults, in HIV-positive children, co-intake of didanosine with a standard meal did not produce significant changes in AUC but delayed the absorption [136].

#### Autoinhibition and Autoinduction

Autoinduction and autoinhibition can modulate the effect of food on antiretroviral drugs and vice versa, dietary interventions may potentially compensate for changes in drug concentrations caused by influence on its own metabolism.

For example, delavirdine can inhibit its own metabolism, so with increasing drug doses, plasma concentrations higher than proportional can be observed [149]. This effect may partially compensate for the potentially negative food impact on pharmacokinetic parameters.

Contrastingly, efavirenz induces CYP enzymes and thereby accelerates its own metabolism. The autoinduction effect varies based on treatment duration—the longer the treatment is, the higher enzyme induction could be. Autoinduction can cause suboptimal therapy effectiveness, so the need for administering an increased efavirenz dose to maintain the optimal clinical response. Moreover, autoinduction contributes to drug resistance development [10]. Autoinduction is the most prevalent among CYP2B6 extensive metabolizers. In CYP2B6 slow metabolizers, the presence of a CYP3A5 genotype allele can be associated with a greater impact of efavirenz autoinduction on plasma drug concentrations [150]. Efavirenz's co-intake with food may potentially mitigate aberrations caused by autoinduction. Increased etravirine bioavailability after co-intake with some types of food may compensate for the metabolic autoinduction effect as well [151]. However, close monitoring of a drug concentration is recommended during the intentional nutritional intervention.

Also ritonavir, as a potent CYP3A4 inhibitor, can induce its own metabolism, causing instability in plasma concentrations during the first 2 weeks of therapy [152]. Irregular drug intake with regard to food may additionally worsen these fluctuations.

#### Inter-individual and Inter-ethnical Variability

When discussing antiretroviral drug-food interactions, the aspect of inter-individual and inter-ethnical variability cannot be overlooked. For example, efavirenz is metabolized primarily by the CYP2B6 enzyme that is characterized by high genetic polymorphism, e.g. the presence of *CYP2B6 516T* allele leads to the 50–75% reduction of enzyme activity, whereas *CYP2B6 785 G* is associated with the increased activity [153]. A meta-analysis from 2019 confirmed that homozygous individuals with the T allele have substantially higher efavirenz plasma concentrations than those with the G allele [154]. *CYP2B6 516G* > *T* polymorphism is more frequent in African-Americans compared to Hispanic, Caucasian, and Asian populations (46%, 27%, 21%, and 17% respectively) [155, 156]. Moreover, approximately 4–12% of African-Americans, are the rare carriers of the CYP2B6*18 variant that inhibits functional protein expression [157]. Therefore, in African-American poor metabolizers, reduced efavirenz clearance is relatively frequent. In consequence, serum drug concentrations are higher, and the risk of side effects increases. The genotype-based efavirenz dose adjustment strategy might be a resolution for the problem of CYP2B6 polymorphism.

Similar to efavirenz, inter-individual variability in nevirapine plasma levels can be observed. The *CYP2B6 516 TT* genotype is associated with increased nevirapine concentrations if compared to the *516TG* and *516GG* genotypes [158]. During the first month of the therapy, nevirapine pharmacokinetic parameters can be unstable due to the autoinduction of CYP3A4 and CYP2B6 enzymes [159]. Fluctuations in plasma drug levels can be partially compensated by administering nevirapine in a constant relationship to food.

In reference to etravirine metabolism, CYP2C19 enzyme genetic polymorphism may occur. In patients with the CYP2C19*2 gene variant, etravirine clearance can be reduced by 23%, leading to a higher drug concentration.

Interestingly, the impact of a low-fat meal on lopinavir absorption depended on the race. In Asian patients, no significant changes in AUC and C_max_ were observed, whereas in Caucasian patients AUC increased by 72%, and C_max_ by 38% [113]. Inter-racial diversity in lopinavir metabolism is obvious in this case. Single nucleotide polymorphism in the CYP3A4*22 gene is responsible for decreased clearance and higher plasma concentrations of lopinavir. This gene variant is more frequent in Caucasians and virtually absent within the East Asian population [12].

### Limitations of Studies Included in the Review

The majority of investigated food-effect studies were randomized, open-label, cross-over clinical trials, as recommended by Food and Drug Administration (FDA). Unfortunately, in almost all cases the randomization process was not described in detail and information was insufficient to judge whether the allocation sequence was truly random and concealed. Additionally, we identified several issues regarding the cross-over design, such as a lack of reporting data from each period of a trial separately, a lack of information on the number of participants allocated to study sequences, or too short washout period. For 15 studies, the design was not specified at all, and 8 were non-randomized clinical trials. To obtain possibly the most complete evidence, we decided to include all food-effect studies (from the 90 s and 00 s as well). In older studies, the methodology was usually described very basically, making it difficult to assess the risk of bias.

Regarding study participants, their number was relatively small, as almost half of the studies (47%) involved only 15 participants or less. However, according to FDA guidelines, the minimum number of participants in a food-effect study should be 12. The apparent limitation is that 73% of studies involved healthy volunteers. Moreover, in almost half of the studies the gender and/or race of participants were not specified, and in the majority of the remaining studies, African-American race was generally the least represented. The results of such studies can be difficult to translate into clinical practice, given the already discussed inter-ethnical variability in response to antiretroviral drugs.

Our findings indicate that drug formulation and type of meal could be potential factors influencing the magnitude of food effects on certain antiretroviral drugs. However, for many of investigated drugs, not all available formulations were tested in the presence of food, and in several studies, it was not even mentioned which drug formulation is being investigated.

Regarding meal composition, in half of the studies, the quantitative or qualitative meal composition was not specified. Another frequent problem was that the same types of meals (e.g. high-fat, high-protein, low-fat, etc.) substantially differed in terms of their qualitative and quantitative composition, and in several studies the patient’s typical diet instead of the standardized meal was tested.

Overall, given all the abovementioned limitations, studies included in this review were judged as having a moderate or high risk of bias (see Supplementary material S3 for details).

### Limitations of the Review

The apparent limitation of our review is the unproportionate evidence of antiretroviral drug-food interactions. Quantitative syntheses were only possible for 16 of 33 antiretroviral drugs since for the remaining, the data on food impact was usually limited to one study, often mentioned only in product characteristics.

Due to the overall high heterogeneity of studies included in this systematic review, a substantial number were not eligible for inclusion in meta-analyses (see Supplementary material S4). The most frequent reasons for exclusion were: inappropriate or unknown study design or effect measure, and missing data of investigated outcomes. According to Cochrane guidelines, the minimal number of studies for subgroup analysis and testing for funnel plot asymmetry should be more than 10 [25]. In this review, the average number of studies in a single meta-analysis was 3–4, and the maximum was 6. Nevertheless, we have performed subgroup analyses to find possible reasons for high heterogeneity, however, their results should be interpreted with caution. The reporting bias cannot be excluded, but we did not investigate it due to the limitations outlined above.

### Final Recommendations

**Fig. 2:**
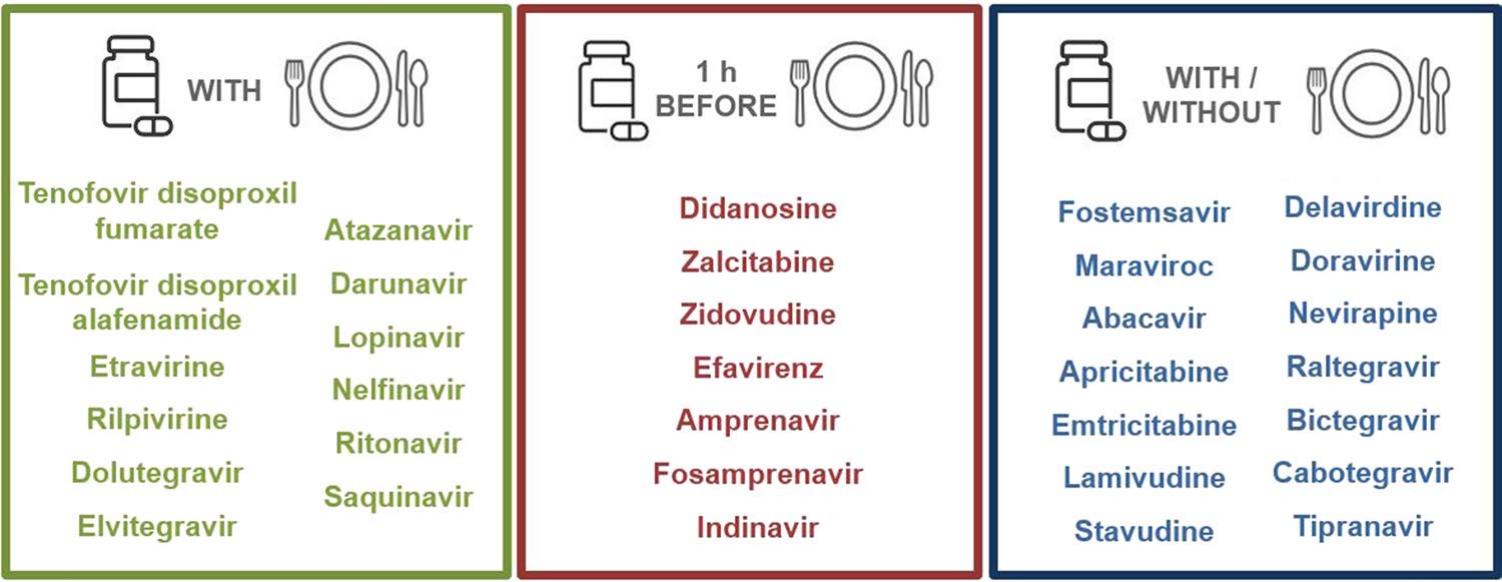
Summary of recommendations for the optimal intake of antiretroviral drugs with regard to meals

The summary of recommendations for the optimal intake of antiretroviral drugs with regard to meals is presented in Fig. 2.

For the drugs included in the qualitative syntheses, the strength of the recommendations is very low, whereas for drugs included in quantitative syntheses is low to moderate, depending on the drug.

The data on the interactions of antiretroviral drugs with dietary supplements, juice, and alcohol is scarce and limited to individual drugs, hence we were not able to outline even general recommendations.

## Summary

In this comprehensive, substantive systematic review, we found evidence for clinically significant interactions with food for more than half of 33 investigated antiretroviral drugs. It is a clear indication that further education about drug-food interactions is necessary. Raising awareness about the proper intake of antiretroviral agents with food should be a priority to optimize HIV patients' cART. Figure 2 shows an overview of recommendations for the optimal intake of antiretroviral drugs with regard to meals.

Our review revealed existing gaps in the knowledge of interactions between antiretroviral drugs and dietary supplements, juice, and alcohol. In our opinion, further in-depth studies are urgently needed. New evidence could be used in the future as the cornerstone of the informed decision-making process regarding HIV therapy.

## Supplementary Information

Below is the link to the electronic supplementary material.

Supplementary file1 (DOCX 2004 KB)

## Data Availability

Not applicable.
