# Supplementary material for: Interactions of Antiretroviral Drugs with Food, Beverages, Dietary Supplements, and Alcohol: A Systematic Review and Meta-analyses
Source: AIDS Behav. 2022 Nov 1;27(5):1441–68. doi: 10.1007/s10461-022-03880-6 (PMC10129904; doi:10.1007/s10461-022-03880-6)
Supplement: Supplementary file 1 — Supplementary file1 (DOCX 2004 KB) [file 10461_2022_3880_MOESM1_ESM.docx]

**Supplementary material S1**

**A detailed searching strategy**

*drug_name****:***

“abacavir”, “apricitabine”, “didanosine”,” emtricitabine”, “lamivudine”, “stavudine”, “tenofovir disoproxil”, “tenofovir alafenamide”, “zalcitabine”, “delavirdine”, “doravirine”, “efavirenz”, “etravirine”, “nevirapine”, “rilpivirine”, “zidovudine”, “dolutegravir”, “elvitegravir”, “raltegravir”, “bictegravir”, “cabotegravir”, “amprenavir”, “atazanavir”, “darunavir”, “fosamprenavir”, “indinavir”, “lopinavir”, “nelfinavir”, “ritonavir”, “saquinavir”, “tipranavir”, “maraviroc”, “fostemsavir”

**Searching strategy in Medline (via Pubmed)**

((*drug_name*[Title/Abstract]) OR (*drug_name*[MeSH Terms])) AND ((food[Title/Abstract])) OR (""food-drug interaction*""[Title/Abstract])) OR (""drug-food interaction*""[Title/Abstract])) OR (meal[Title/Abstract])) OR (diet[Title/Abstract])) OR (breakfast[Title/Abstract])) OR (""dietary supplement*""[Title/Abstract])) OR (alcohol[Title/Abstract])) OR (juice*[Title/Abstract]))

**Searching strategy in Embase**

'*drug_name*':ti,ab AND ('food':ti,ab OR 'food drug interaction':ti,ab OR 'meal':ti,ab OR 'diet':ti,ab OR breakfast:ti,ab OR 'dietary supplement':ti,ab OR 'alcohol':ti,ab OR juice:ti,ab)

**Searching strategy in Google Scholar**

allintitle: *drug_name* food OR diet OR meal OR breakfast OR "dietary supplement" OR "food-drug interaction" OR "drug-food interaction" OR alcohol OR juice

**Supplementary material S2**

| Table S1. A detailed description of studies investigating interactions between NRTIs and food. | | | | | | | | |
| --- | --- | --- | --- | --- | --- | --- | --- | --- |
| Drug | Food type | Study details | Participants | Drug dose [mg] | Drug formulation | Observed effect | Quantitative meal composition | Qualitative meal composition |
| Abacavir | High-fat meal | Chittick et al. [1]  randomized, open-label, cross-over clinical trial | 18, HIV (+)  11 males, 7 females  2 Caucasian, 8 African-American, 8 Hispanic | 300 | tablets | no significant changes in AUC, C_max_ ↓ 26%, t_max_ ↑ 0.5 h | 1000 kcal, 67 g of fat, 58 g of carbohydrates, and 33 g of protein | 2 slices of toasted white bread with butter, 2 eggs fried in butter, 2 slices of bacon (15 g), 2 oz (57 g) hash-browned potatoes, and 8 oz (237 mL) whole milk |
|  | High-fat meal | Yuen et al. [2]  randomized, open-label, cross-over clinical trial | 24, healthy  15 males, 9 females  13 Caucasian, 9 Hispanic, 1 African-American, 1 Asian | 300 | tablets | no significant changes in AUC, C_max_ ↓ 32%, t_max_ ↑ 1 h | 1000 kcal, 67 g of fat, 58 g of carbohydrates, and 33 g of protein | 2 slices of toasted white bread with butter, 2 eggs fried in butter, 2 slices of bacon (15 g), 2 oz (57 g) hash-browned potatoes, and 8 oz (237 mL) whole milk |
|  | High-fat meal | Weller et al. [3]  randomized, open-label, cross-over clinical trial | 12, healthy  8 males, 4 females  7 Caucasian, 5 African-American | 300 | tablets | no significant changes in AUC, C_max_ ↓ 23%, t_max_ not specified | 869 kcal, 51.2 g of fat | 2 eggs fried in butter, 2 strips of bacon, 2 slices of toast with butter, 4 oz (114 g) of hash brown potatoes, and 8 oz (237 mL) of whole milk |
|  | Not specified | Marier et al. [4]  randomized, cross-over clinical trial | 80, healthy Gender and race not specified | 300 | tablets | AUC ↓ 19% - considered clinically insignificant, C_max_ ↓ 28%, t_max_ not specified | not specified | not specified |
| Apricitabine | High-fat meal | Holdich et al.[5]  randomized, open-label, cross-over clinical trial | 12, healthy  All males  Race not specified | 1200 | capsules | no significant changes in AUC and C_max_, t_max_ ↑ 1.3 h | not specified | not specified |
| Didanosine | High-fat meal | Knupp et al. [6]  randomized, open-label, cross-over clinical trial | 10, HIV (+)  All males  Race not specified | 300 | tablets | AUC ↓ 55%, C_max_ ↓ 60%, no significant changes in t_max_ | 802 kcal, 40.3 g of fat, 71.4 g of carbohydrates, and 26.2 g of protein | 1 slice of white toast with a pat of butter and jelly, 2 soft or hardboiled eggs, 4 oz (114g) of hash-browned potatoes, 2 strips of bacon (30g), 8 oz (237 mL) of whole milk |
|  | High-fat meal | Damle et al. [7]  randomized, open-label, cross-over clinical trial | 20, healthy  Males and females  Race not specified | 400 | enteric-coated capsules | AUC ↓ 19%, C_max_ ↓ 46%, t_max_ ↑ 3 h | 757 kcal, 41.2 g of fat, 66.2 g of carbohydrates, and 30.3 g of protein | 2 slices of white toasted bread, 2 eggs, 2 teaspoons of butter, 1 tablespoon of jelly, 2 strips of bacon, 4 oz (114 g) of hash brown potatoes, and 8 oz (237 mL) of whole milk |
|  | High-fat meal | Hernández-Novoa et al. [8]  randomized, open-label trial | 21, HIV (+) 16 males, 5 females Race not specified | 250-400 (adjusted to b.w.) | enteric-coated capsules | no significant changes in antiretroviral activity after 28 days | 350 kcal | not specified |
|  | Standard meal | Shyu et al. [9]  non-randomized, open-label, cross-over clinical trial | 8, HIV (+)  All males  Race not specified | 375 | tablets | AUC ↓ 47%, C_max_ ↓ 54%, no significant changes in t_max_ | not specified | 1 slice of toast with a pat of butter and jelly, 2 eggs, 2 slices of bacon (30 g), 4 oz (114 g) hash-browned potatoes, and 8 oz (237 mL) whole milk |
|  | Standard meal | Kearney et al. [10]  non-randomized, open-label, cross-over clinical trial | 28, healthy  17 males, 11 females  24 Caucasian, 4 non-Caucasian | 250 | enteric-coated capsules | AUC ↓ 20%, C_max_ ↓ 25%, t_max_ ↑ 1.5 h | 373 kcal, contained 8.3 g of fat | 2 pieces of toast with butter and jelly, 8 oz (237 mL) of orange juice, and 8 oz (237 mL) of low-fat milk |
|  | Standard meal | Stevens et al. [11]  randomized clinical trial | 77, HIV (+) children  40 males, 37 females  23 Caucasian, 24 African-American, 28 Hispanic, 2 Other | 100-300/m^2^ | powder to prepare the oral solution | no significant changes in AUC, C_max_ not specified, t_max_ ↑ 0.3 h, t_1/2_ ↑ 0.5 h | Not specified | Patient’s normal diet |
|  | Low-fat meal | Damle et al. [7]  randomized, open-label, cross-over clinical trial | 25, healthy  Males and females  Race not specified | 400 | enteric-coated capsules | AUC ↓ 27%, C_max_ ↓ 22%, t_max_ ↑ 2.5 h | 373 kcal, contained 8.3 g of fat, 63.4 g of carbohydrates, and 11.2 g of protein | 2 slices of white toasted bread, 1 teaspoon of low-fat margarine, 1 tablespoon of jelly, 5 oz (148 mL) of orange juice, 5 oz (148 mL) of skim milk |
|  | Yoghurt | Damle et al. [7]  randomized, open-label, cross-over clinical trial | 28, healthy  Males and females  Race not specified | 400 | enteric-coated capsule contents | AUC ↓ 20%, C_max_ ↓ 30%, no significant changes in t_max_ | 69 kcal, 0.27 g of fat | 2 tablespoons of Dannon plain yogurt |
|  | Apple mouse | Damle et al. [7]  randomized, open-label, cross-over clinical trial | 28, healthy  Males and females  Race not specified | 400 | enteric-coated capsule contents | AUC ↓ 18%, C_max_ ↓ 24%, no significant changes in t_max_ | 25 kcal | 2 tablespoons of Mott’s apple sauce |
|  | Not specified | Lopez et al. [12]  retrospective, cohort study | 668, HIV (+)  523 males, 145 females  Race not specified | not specified | enteric-coated capsules | in patients with poor adherence to ART (<80%): HR of virological failure – 8.32 when didanosine is taken with food  in patients with good adherence to ART (>80%): HR of virological failure not significantly increased when didanosine is taken with food | not specified | not specified |
|  | Not specified | Sánchez et al. [13]  prospective cohort trial | 103, HIV (+)  71 males, 32 females  Race not specified | 400, 4x day, for 48 weeks | enteric-coated capsules | comparable mean plasma didanosine concentrations  no significant differences in plasma HIV-RNA count, mean CD4 gain, and frequency of virological failure | not specified | not specified |
| Emtricitabine | High-fat meal | Wang et al. [14]  study design not specified | 12, HIV (+)  Gender and race not specified | 400 | tablets | no significant changes in AUC, C_max_, t_max_ | not specified | not specified |
|  | High-fat meal | Crauwels et al. [15]  randomized, open-label, cross-over clinical trial | 24, healthy  12 males, 12 females  All Caucasian | 200 | tablets | no significant changes in AUC, C_max_, t_max_ | 928 kcal, 56 g of fat | 2 slices of white bread with butter, 2 eggs fried in butter, 2 strips of bacon, 1 croissant with 1 slice of cheese, and 8 oz (240 mL) of whole milk |
|  | High-fat meal | Bictarvy prescribing information [16]  study design not specified | 12, healthy  Gender and race not specified | 200 | tablets | no significant changes in AUC, C_max_, t_max_ | 800 kcal, 44.4 g of fat | not specified |
|  | High-fat meal | Han et al. [17]  randomized, open-label clinical trial | 60, healthy  All males  All Asian | 200 | capsules | no significant changes In AUC, C_max_, t_max_, and t_1/2_ after single and multiple doses | 45% of fat | not specified |
|  | High-fat meal | Majeed et al. [18]  randomized, open-label, cross-over clinical trial | 52, healthy  Gender and race not specified | 120 | tablets | no significant changes in AUC and C_max_, t_max_ not specified | 1000 kcal, 50% of fat | not specified |
|  | High-protein meal | Shiomi et al. [19]  randomized, open-label, cross-over clinical trial | 11, healthy  All males  All Asian | 200 | tablets | no significant changes in AUC, C_max_, t_max_ | 250 kcal, 8.8 g of fat, 34.3 g of carbohydrates, and 8.8 g of protein | 250 mL of Ensure protein-rich drink |
|  | High-protein meal | Yamada et al. [20]  randomized, open-label, cross-over clinical trial | 12, healthy  All males  All Asian | 200 | tablets | no significant changes in AUC, C_max_, t_max_ | 250 kcal, 8.8 g of fat, 34.3 g of carbohydrates, and 8.8 g of protein | 250 mL of Ensure protein-rich drink |
|  | Standard meal | Lamorde et al. [21]  non-randomized, open-label, cross-over clinical trial | 15, HIV (+)  11 males, 4 females  All African-American | 200 | tablets | no clinically significant changes in AUC, C_max_, t_max_ | 650 kcal, 19 g of fat | local bananas, cooked vegetables, oil and meat, and tea with milk |
|  | Standard meal | Custodio et al. [22]  randomized, open-label, cross-over clinical trial | 24, healthy  12 males, 12 females  22 Caucasian, 2 African-American | 200 | tablets | no clinically significant changes in AUC, C_max_, t_max_ | 540 kcal, 21 g of fat | 2 slices of white bread, 1 pat of butter, 1 slice of cheese, 2 slices of ham, 1 packet of jelly, 1 cup of decaffeinated coffee, 1 packet of sugar, and 1 cup of 2 % milk |
|  | Standard meal | Shiomi et al. [19]  randomized, open-label, cross-over clinical trial | 11, healthy  All males  All Asian | 200 | tablets | no significant changes in AUC, C_max_, t_max_ | 413 kcal, 9.6 g of fat, 72.2 g of carbohydrates, and 11.4 g of protein | 1 tuna, lettuce, and tomato sandwich, 1 ham, cheese, and egg sandwich, 1 cup of white peach jelly, and 160 g of apple juice |
|  | Standard meal | Yamada et al. [20]  randomized, open-label, cross-over clinical trial | 12, healthy  All males  All Asian | 200 | tablets | no significant changes in AUC, C_max_, t_max_ | 413 kcal, 9.6 g of fat, 72.2 g of carbohydrates, and 11.4 g of protein | 2 slices of bread with strawberry jam, 1 boiled egg, and 160 g of grape juice |
|  | Low-fat meal [22] | Custodio et al. [22]  randomized, open-label, cross-over clinical trial | 24, healthy  12 males, 12 females  22 Caucasian, 2 African-American | 200 | tablets | no clinically significant changes in AUC, C_max_, t_max_ | 390 kcal, 12 g of fat | 2 slices of wheat bread, 1 tablespoon of Benecol Light Spread, 8 oz (237 mL) of 2 % milk |
| Lamivudine | High-fat meal | Yuen et al. [2]  randomized, open-label, cross-over clinical trial | 24, healthy  15 males, 9 females  13 Caucasian, 9 Hispanic, 1 African-American, 1 Asian | 150 | tablets | no significant changes in AUC, C_max_, t_max_ | 1000 kcal, 67 g of fat, 58 g of carbohydrates, and 33 g of protein | 2 slices of toasted white bread with butter, 2 eggs fried in butter, 2 slices of bacon (15 g), 2 oz (57 g) hash-browned potatoes, and 8 oz (237 mL) whole milk |
|  | High-fat meal | Moore et al. [23]  randomized, open-label, cross-over clinical trial | 24, healthy  12 males, 12 females  16 Caucasian, 5 Hispanic, 2 African-American, 1 Oriental | 150 | tablets | no significant changes in AUC and C_max_, t_max_ ↑ 1 h | 1000 kcal, 67 g of fat, 58 g of carbohydrates, and 33 g of protein | 2 slices of toasted white bread with butter, 2 eggs fried in butter, 2 slices of bacon (15 g), 2 oz (57 g) hash-browned potatoes, and 8 oz (237 mL) whole milk |
|  | High-fat meal | Dumitrescu et al. [24]  randomized, open-label, cross-over clinical trial | 16, healthy  Gender and race not specified | 300 | tablets (2 formulations compared) | no significant changes in AUC, C_max_ ↓ 22-32%, t_max_ ↑ 1-75-2.5 h – both probably clinically insignificant | 1000 kcal, 67 g of fat, 58 g of carbohydrates, and 33 g of protein | 2 slices of toasted white bread with butter, 2 eggs fried in butter, 2 slices of bacon (15 g), 2 oz (57 g) hash-browned potatoes, and 8 oz (237 mL) whole milk |
|  | High-fat meal | Behm et al. [25]  randomized, open-label, cross-over clinical trial | 14, healthy  9 males, 5 females  8 Caucasian, 4 African-American, 1 Multi-racial, 1 Native Hawaiian | 300 | tablets | no significant changes in AUC, C_max_ ↓ 19%, t_max_ ↑ 2 h – both considered clinically irrelevant | 900 kcal, 55.6 g of fat, 62.5 g of carbohydrates, and 37.5 g of protein | not specified |
|  | High-fat meal | Weller et al. [3]  randomized, open-label, cross-over clinical trial | 12, healthy  8 males, 4 females  7 Caucasian, 5 African-American | 300 | tablets | no significant changes in AUC and C_max_, t_max_ not specified | 869 kcal, 51.2 g of fat | 2 eggs fried in butter, 2 strips of bacon, 2 slices of toast with butter, 4 oz (114 g) of hash brown potatoes, and 8 oz (237 mL) of whole milk |
|  | High-fat meal | Epivir prescribing information [26]  study design not specified | 12, HIV (+)  Gender and race not specified | 25 | not specified | no significant changes in AUC, C_max_ ↓ 40%, t_max_ ↓ 2.3 h | 1099 kcal, 75 g of fat, 72 g of carbohydrates, 34 g of protein | not specified |
|  | Standard meal | Angel et al. [27]  randomized, cross-over clinical trial | 12, HIV (+)  All males  Race not specified | not specified | not specified | no significant changes in AUC, C_max_ ↓ 47%, t_max_ ↑ 2.3 h | not specified | not specified |
|  | Pudding | Yee et al. [28]  randomized, open-label, cross-over clinical trial | 12 of all 24, healthy  13 males, 11 females  13 Caucasian, 7 African-American, 4 Asian | 300 | uncoated granules | AUC ↓ 21%, C_max_ ↓ 19% - considered as clinically insignificant, t_max_ unchanged | not specified | 2 oz (57 g) |
|  | Pudding | Yee et al. [28]  randomized, open-label, cross-over clinical trial | 10 of all 24, healthy  13 males, 11 females  13 Caucasian, 7 African-American, 4 Asian | 300 | coated granules | AUC↓ 15%, C_max_ ↓ 15% - considered as clinically insignificant, t_max_ unchanged | not specified | 2 oz (57 g) |
|  | Apple sauce | Yee et al. [28]  randomized, open-label, cross-over clinical trial | 12 of all 24, healthy  13 males, 11 females  13 Caucasian, 7 African-American, 4 Asian | 300 | uncoated granules | AUC↓ 22%, C_max_ ↓ 25% - considered as clinically insignificant, t_max_ unchanged | not specified | 2 oz (57 g) |
|  | Apple sauce | Yee et al. [28]  randomized, open-label, cross-over clinical trial | 10 of all 24, healthy  13 males, 11 females  13 Caucasian, 7 African-American, 4 Asian | 300 | coated granules | AUC↓ 15%, C_max_ ↓ 15% - considered as clinically insignificant, t_max_ unchanged | not specified | 2 oz (57 g) |
| Stavudine | High-fat meal | Kaul et al. [29]  randomized, open-label, cross-over clinical trial | 17, HIV (+)  13 males, 4 females  9 Caucasian, 3 African-American, 5 Hispanic | 70 | capsules | no significant changes in AUC, C_max_ ↓ 47%, t_max_ ↑ 1.1 h | 773 kcal, 45.5 g of fat, 61.8 g of carbohydrates, and 29 g of protein | 1 slice of toast with a pat of butter and jelly, 2 boiled eggs, 2 slices of bacon, 4 oz (114 g) hash-browned potatoes, and 8 oz (237 mL) whole milk |
| Tenofovir disoproxil | High-fat meal | Viread prescribing information [30]  study design not specified | 12, healthy  Gender and race not specified | 300 | tablets | AUC ↑ 40%, C_max_ ↑ 14%, t_max_ ↑ 1 h | 850 kcal, 42.5 g of fat | not specified |
|  | High-fat meal | Behm et al. [25]  randomized, open-label, cross-over clinical trial | 14, healthy  9 males, 5 females  8 Caucasian, 4 African-American, 1 Multi-racial, 1 Native Hawaiian | 300 | tablets | AUC ↑ 26%, unchanged C_max_, t_max_ ↑ 2 h – all considered clinically insignificant | 900 kcal, 55.6 g of fat, 62.5 g of carbohydrates, and 37.5 g of protein | not specified |
|  | High-protein meal | Shiomi et al. [19]  randomized, open-label, cross-over clinical trial | 11, healthy  All males  All Asian | 300 | tablets | AUC ↑ 24%, C_max_ ↑ 23%, no significant changes in t_max_ | 250 kcal, 8.8 g of fat, 34.3 g of carbohydrates, and 8.8 g of protein | 250 mL of Ensure protein-rich drink |
|  | Standard meal | Lamorde et al. [21]  non-randomized, open-label, cross-over clinical trial | 15, HIV (+)  11 males, 4 females  All African | 300 | tablets | no clinically significant changes in AUC, C_max,_ and t_max_ | 650 kcal, 19 g of fat | local bananas, cooked vegetables, oil and meat, and tea with milk |
|  | Standard meal | Custodio et al. [22]  randomized, open-label, cross-over clinical trial | 24, healthy  12 males, 12 females  22 Caucasian, 2 African-American | 300 | tablets | AUC ↑ 37%, C_max_ ↑ 32%, no significant changes in t_max_ | 540 kcal, 21 g of fat | 2 slices of white bread, 1 pat of butter, 1 slice of cheese, 2 slices of ham, 1 packet of jelly, 1 cup of decaffeinated coffee, 1 packet of sugar, and 1 cup of 2 % milk |
|  | Standard meal | Shiomi et al. [19]  randomized, open-label, cross-over clinical trial | 11, healthy  All males  All Asian | 300 | tablets | AUC ↑ 37%, C_max_ ↑ 36%, no significant changes in t_max_ | 413 kcal, 9.6 g of fat, 72.2 g of carbohydrates, and 11.4 g of protein | 1 tuna, lettuce, and tomato sandwich, 1 ham, cheese, and egg sandwich, 1 cup of white peach jelly, and 160 g of apple juice |
|  | Low-fat meal | Custodio et al. [22]  randomized, open-label, cross-over clinical trial | 24, healthy  12 males, 12 females  22 Caucasian, 2 African-American | 300 | tablets | AUC ↑ 28%, C_max_ ↑ 14%, no significant changes in t_max_ | 390 kcal, 12 g of fat | 2 slices of wheat bread, 1 tablespoon of Benecol Light Spread, 8 oz (237 mL) of 2% milk |
|  | Pudding | Yee et al. [28]  randomized, open-label, cross-over clinical trial | 12 of all 24, healthy  13 males, 11 females  13 Caucasian, 7 African-American, 4 Asian | 300 | uncoated granules | AUC ↑ 13%, C_max_ ↑ 22% - considered as clinically insignificant, t_max_ unchanged | not specified | 2 oz (57 g) |
|  | Pudding | Yee et al. [28]  randomized, open-label, cross-over clinical trial | 10 of all 24, healthy  13 males, 11 females  13 Caucasian, 7 African-American, 4 Asian | 300 | coated granules | AUC ↑ 18%, C_max_ ↑ 23% - considered as clinically insignificant, t_max_ unchanged | not specified | 2 oz (57 g) |
|  | Apple sauce | Yee et al. [28]  randomized, open-label, cross-over clinical trial | 12 of all 24, healthy  13 males, 11 females  13 Caucasian, 7 African-American, 4 Asian | 300 | uncoated granules | AUC ↑ 20%, C_max_ ↑ 23% - considered as clinically insignificant, t_max_ unchanged | not specified | 2 oz (57 g) |
|  | Apple sauce | Yee et al. [28]  randomized, open-label, cross-over clinical trial | 10 of all 24, healthy  13 males, 11 females  13 Caucasian, 7 African-American, 4 Asian | 300 | coated granules | AUC ↑ 15%, C_max_ ↑ 20% - considered as clinically insignificant, t_max_ unchanged | not specified | 2 oz (57 g) |
| Tenofovir dipivoxil | High-fat meal | Lu et al. [31]  randomized, open-label, cross-over clinical trial | 12, healthy  6 males, 6 females  All Asian | 300 | tablets | AUC ↑ 23%, no significant changes in C_max_, t_max_ ↑ 0.5 h | 900 kcal, 55.6 g of fat, 62.5 g of carbohydrates, and 37.5 g of protein | 2 pieces of butter bread, 2 slices of hams, 2 fried eggs, a potato salad, and 250 mL of whole milk |
| Tenofovir alafenamide | High-fat meal | Crauwels et al. [15]  randomized, open-label, cross-over clinical trial | 24, healthy  12 males, 12 females  All Caucasian | 10 | tablets | AUC ↑ 15%, C_max_ ↓ 40%, t_max_ ↑ 25 min. | 928 kcal, 56 g of fat | 2 slices of white bread with butter, 2 eggs fried in butter, 2 strips of bacon, 1 croissant with 1 slice of cheese, and 8 oz (237 mL) of whole milk |
|  | High-fat meal | Bictarvy prescribing information [16]  study design not specified | 12, healthy  Gender and race not mentioned | 25 | tablets | AUC ↑ 63%, C_max_ ↓ 8%, t_max_ not specified | 800 kcal, 50% of fat | not specified |
|  | High-fat meal | Custodio et al. [32]  randomized, open-label, cross-over clinical trial | 40, healthy  24 males, 16 females  29 Caucasian,  11 non-Caucasian | 25 | tablets | AUC ↑ 82%, no significant changes in C_max_, t_max_ not specified | 800 kcal, 50% of fat | not specified |
|  | High-fat meal | Custodio et al. [32]  randomized, open-label, cross-over clinical trial | 43, healthy  30 males, 13 females  22 Caucasian,  21 non-Caucasian | 10 | tablets | AUC ↑ 14%, no significant changes in C_max_, t_max_ not specified | 800 kcal, 50% of fat | not specified |
|  | High-fat meal | Li et al. [33]  randomized, open-label, cross-over clinical trial | 73, healthy  51 males, 22 females  All Asian | 25 | tablets (2 formulations compared) | AUC ↑ 87-98%, no significant changes in C_max_, t_max_ ↑ 0.5 h | 900 kcal, 55.6 g of fat, 62.5 g of carbohydrates, and 37.5 g of protein | not specified |
|  | High-fat meal | Li et al. [34]  randomized, open-label, cross-over clinical trial | 67, healthy  42 males, 25 females  All Asian | 25 | tablets (2 formulations compared) | AUC ↑ 56%, no significant changes in C_max_, t_max_ ↑ 1.2-1.5 h | 900 kcal, 55.6 g of fat, 62.5 g of carbohydrates, and 37.5 g of protein | not specified |
|  | High-fat meal | Li et al. [35]  randomized, open-label, parallel clinical trial | 64, healthy  52 males, 12 females  All Asian | 25 | tablets | AUC ↑ 60%, C_max_ ↓ 21%, t_max_ ↑ 0.7 h | 800-1000 kcal | not specified |
|  | High-fat meal | Majeed et al. [18]  randomized, open-label, cross-over clinical trial | 52, healthy  Gender and race not specified | 15 | tablets | AUC ↑ 42%, C_max_ ↓ 44%, t_max_ not specified | 1000 kcal, 50% of fat | not specified |
|  | High-protein meal | Yamada et al. [20]  randomized, open-label, cross-over clinical trial | 12, healthy  All males  All Asian | 10 | tablets | no significant changes in AUC and C_max_, t_max_ ↑ 0.5 h | 250 kcal, 8.8 g of fat, 34.3 g of carbohydrates, and 8.8 g of protein | 250 mL of Ensure protein-rich drink |
|  | Standard meal | Yamada et al. [20]  randomized, open-label, cross-over clinical trial | 12, healthy  All males  All Asian | 10 | tablets | no significant changes in AUC and C_max_, t_max_ ↑ 0.5 h | 413 kcal, 9.6 g of fat, 72.2 g of carbohydrates, and 11.4 g of protein | 2 slices of bread with strawberry jam, 1 boiled egg, and 160 g of grape juice |
| Zalcitabine | Standard meal | Nazareno et al. [36]  randomized, open-label, cross-over clinical trial | 20, HIV (+)  18 males, 2 females  Race not mentioned | 1,5 | tablets | no significant changes in AUC, C_max_ ↓ 39%, t_max_ ↑ 0.8 h | not specified | 2 slices of white toast, 3 teaspoons of butter/margarine, 2 strips of bacon, 2 fried eggs, 4 oz (114 g) of hush-brown potatoes, 8 oz (237 mL) of whole milk |
| Zidovudine | High-fat meal | Unadkat et al. [37]  randomized, cross-over clinical trial | 6, HIV (+)  All males  Race not mentioned | 100 | tablets | AUC not specified, C_max_ ↓ 50%, t_max_ ↑ 1.3 h | 945 kcal, 52.5 g of fat, 52 g of carbohydrates, 66 g of protein | not specified |
|  | High-fat meal | Yuen et al. [2]  randomized, open-label, cross-over clinical trial | 24, healthy  15 males, 9 females  13 Caucasian, 9 American-Hispanic, 1 African-American, 1 Asian | 300 | tablets | no significant changes in AUC and C_max_, t_max_ ↑ 1 h | 1000 kcal, 67 g of fat, 58 g of carbohydrates, and 33 g of protein | 2 slices of toasted white bread with butter, 2 eggs fried in butter, 2 slices of bacon (15 g), 2 oz (57 g) hash-browned potatoes, and 8 oz (237 mL) whole milk |
|  | High-fat meal | Moore et al. [23]  randomized, open-label, cross-over clinical trial | 24, healthy  12 males, 12 females  16 Caucasian, 5 Hispanic, 2 African-American, 1 Oriental | 300 | tablets | no significant changes in AUC, C_max_ ↓ 45%, t_max_ ↑ 0.5 h | 1000 kcal, 67 g of fat, 58 g of carbohydrates, and 33 g of protein | 2 slices of toasted white bread with butter, 2 eggs fried in butter, 2 slices of bacon (15 g), 2 oz (57 g) hash-browned potatoes, and 8 oz (237 mL) whole milk |
|  | High-fat meal | Lotterer et al. [38]  randomized, cross-over clinical trial | 13, HIV (+)  10 males, 3 females  Race not specified | 250 | capsules | no significant changes in AUC, C_max_ ↓ 64%, t_max_ ↑ 1 h | 600 kcal, 40 g of fat | not specified |
|  | High-fat meal | Shelton et al. [39]  randomized, cross-over clinical trial | 18, HIV (+)  12 males, 6 females  Race not specified | 100 | not specified | no significant changes in AUC, C_max_ ↓ 58%, t_max_ ↑ 1 h | Not specified | Not specified |
|  | High-protein meal | Sahai et al. [40]  randomized, cross-over clinical trial | 11, HIV (+)  All males  Race not specified | 200 | capsules | no significant changes in AUC and t_max_, C_max_ ↓ 32% | 132 kcal, 25 g of protein | 33g of protein supplement in 220 mL of orange juice |
|  | Standard meal | Ruhnke et al. [41]  randomized clinical trial | 12 of all 27, HIV (+)  24 males, 3 females  Race not specified | 100 | capsules | AUC↓ 33%, C_max_ ↓ 37%, no significant changes in t_max_ | 600 kcal | 2 slices of white bread, jam, butter, sliced Swiss cheese or ham, black tea or coffee |
|  | Standard meal | Ruhnke et al. [41]  randomized clinical trial | 15 of all 27, HIV (+)  24 males, 3 females  Race not specified | 250 | capsules | AUC↓ 13%, C_max_ ↓ 73%, t_max_ ↑ 1.2 h | 600 kcal | 2 slices of white bread, jam, butter, sliced Swiss cheese or ham, black tea or coffee |
|  |  |  |  |  |  |  |  |  |

**Table S2. A detailed description of studies investigating interactions between NNRTIs and food.**

| **Drug** | **Food type** | **Study details** | **Participants** | **Drug dose [mg]** | **Drug formulation** | **Observed effect** | **Quantitative meal composition** | **Qualitative meal composition** |
| --- | --- | --- | --- | --- | --- | --- | --- | --- |
| **Delavirdine** | Standard meal | Morse et al. [42]  randomized, open-label, cross-over clinical trial | 13, HIV (+)  11 males, 2 females  8 Caucasian, 5 Hispanic | 400 | tablets | no significant changes in AUC, C_min,_ and t_max_, C_max_ ↓ 21% | not specified | patient's standard diet |
| **Doravirine** | High-fat meal | Behm et al. [25]  randomized, open-label, cross-over clinical trial | 14, healthy  7 males, 7 females  7 Caucasian, 6 African-American, 1 Multi-racial | 100 | tablets | no significant changes in AUC and C_max_, t_max_ ↑ 1.5 h | 900 kcal, 55.6 g of fat, 62.5 g of carbohydrates, and 37.5 g of protein | not specified |
|  | High-fat meal | Behm et al. [25]  randomized, open-label, cross-over clinical trial | 14, healthy  9 males, 5 females  8 Caucasian, 4 African-American, 1 Multi-racial, 1 Native Hawaiian | 100 | tablets | no significant changes in AUC and C_max_, t_max_ ↑ 3 h | 900 kcal, 55.6 g of fat, 62.5 g of carbohydrates, and 37.5 g of protein | not specified |
|  | High-fat meal | Anderson et al. [43]  randomized, double-blind, placebo controlled, parallel clinical trial | 8 of 50, healthy  All males  46 Caucasian, 3 African-American, 1 Asian | 50 | tablets | AUC ↑ 33%, no significant changes in Cmax, t_max_ ↑ 4 h | 744 kcal, 55.6 g of fat, 55 g of carbohydrates, and 6 g of protein | not specified |
|  | Pudding | Yee et al. [28]  randomized, open-label, cross-over clinical trial | 12 of all 24, healthy  13 males, 11 females 13 Caucasian, 7 African-American, 4 Asian | 100 | uncoated granules | no significant changes in AUC, C_max_, and t_max_ | not specified | 2 oz (57 g) |
|  | Pudding | Yee et al. [28]  randomized, open-label, cross-over clinical trial | 10 of all 24, healthy 13 males, 11 females 13 Caucasian, 7 African-American, 4 Asian | 100 | coated granules | no significant changes in AUC, C_max_, and t_max_ | not specified | 2 oz (57 g) |
|  | Apple sauce | Yee et al. [28]  randomized, open-label, cross-over clinical trial | 12 of all 24, healthy 13 males, 11 females 13 Caucasian, 7 African-American, 4 Asian | 100 | uncoated granules | AUC ↑ 29%, C_max_ ↑ 56% - clinically irrelevant, no significant changes in t_max_ | not specified | 2 oz (57 g) |
|  | Apple sauce | Yee et al. [28]  randomized, open-label, cross-over clinical trial | 11 of all 24, healthy 13 males, 11 females 13 Caucasian, 7 African-American, 4 Asian | 100 | coated granules | AUC ↑ 26%, C_max_ ↑ 59% - clinically irrelevant, no significant changes in t_max_ | not specified | 2 oz (57 g) |
| **Efavirenz** | High-fat meal | Sustiva prescribing information [44]  study design not specified | 12, healthy Gender, and race not specified | 600 | capsules | AUC ↑ 22%, C_max_ ↑ 39%, t_max_ not specified | 894 kcal, 54 g of fat | not specified |
|  | High-fat meal | Sustiva prescribing information [44]  study design not specified | 12, healthy Gender and race not specified | 600 | tablets | AUC ↑ 28%, C_max_ ↑ 79%, t_max_ not specified | 1000 kcal, 61 g of fat | not specified |
|  | Standard meal | Lamorde et al. [21]  non-randomized, open-label, cross-over clinical trial | 15, HIV (+)  11 males, 4 females All African | 600 | tablets | AUC ↑ 13%, C_max_ ↑ 47%, t_max_ not specified | 650 kcal, 19 g of fat | local bananas, cooked vegetables, oil and meat, and tea with milk |
|  | Low-fat meal | Sustiva prescribing information [44]  study design not specified | 12, healthy Gender and race not specified | 600 | capsules | AUC ↑ 17%, C_max_ ↑ 51%, t_max_ not specified | 440 kcal, 2 g of fat | not specified |
|  | Apple mouse | Kaul et al. [45]  randomized, open-label, cross-over clinical trial | 12 of all 24, healthy  23 males, 1 female  12 Caucasian, 12 African-American | 600 | crushed | no significant changes in AUC and C_max_, t_max_ not specified | 4 kcal | 2 teaspoons |
|  | Jelly | Kaul et al. [45]  randomized, open-label, cross-over clinical trial | 12 of all 24, healthy  23 males, 1 female  12 Caucasian, 12 African-American | 600 | crushed | no significant changes in AUC and C_max,_  t_max_ not specified | 33 kcal | 2 teaspoons |
|  | Yoghurt | Kaul et al. [45]  randomized, open-label, cross-over clinical trial | 12 of all 24, healthy  23 males, 1 female  12 Caucasian, 12 African-American | 600 | crushed | no significant changes in AUC and C_max,_  t_max_ not specified | 7.5 kcal | 2 teaspoons |
|  | Infant formula | Kaul et al. [45]  randomized, open-label, cross-over clinical trial | 12 of all 24, healthy  23 males, 1 female  12 Caucasian, 12 African-American | 600 | crushed | no significant changes in AUC and C_max,_  t_max_ not specified | 7 kcal | 2 teaspoons |
| **Etravirine** | High-fat meal | Schöller-Gyüre et al. [46]  randomized, open-label, cross-over clinical trial | 12 of all 24, healthy  All males  23 Caucasian, 1 African-American | 100 | tablets | AUC ↑ 31%, C_max_ ↑ 46%, t_max_ ↑ 2 h | 1160 kcal, 70.3 g of fat, 91.3 g of carbohydrates, and 40.4 g of protein, 2.2 g of fiber | 2 fried eggs, 2 slices of fried bacon, 1 croissant, 2 slices of bread, butter, 30 g of chocolate, 1 cup tea or coffee |
|  | High-fiber meal | Schöller-Gyüre et al. [46]  randomized, open-label, cross-over clinical trial | 12 of all 24, healthy  All males  23 Caucasian, 1 African-American | 100 | tablets | no significant changes in AUC and C_max_, t_max_ ↑ 1 h | 685 kcal, 3.1 g of fat, 151.2 g of carbohydrates, and 13.4 g of protein, 16.4 g of fiber | 80 g each of grapes, pineapple, pears, fiber, and strawberries; 1 banana, 2 slices of bread, jam, 1 glass of orange juice |
|  | Standard meal | Schöller-Gyüre et al. [46]  randomized, open-label, cross-over clinical trial | 12 of all 24, healthy  All males  23 Caucasian, 1 African-American | 100 | tablets | AUC ↑ 54%, C_max_ ↑ 45%, t_max_ ↑ 2 h | 561 kcal, 15.3 g of fat, 83.9 g of carbohydrates, and 21.9 g of protein, 8.1 g of fiber | 4 slices of bread, 2 slices of ham or cheese, butter, jam, 2 cups of tea or coffee |
|  | Low-fat meal | Schöller-Gyüre et al. [46]  randomized, open-label, cross-over clinical trial | 12 of all 24, healthy  All males  23 Caucasian, 1 African-American | 100 | tablets | AUC ↑ 29%, C_max_ ↑ 44%, t_max_ ↑ 1 h | 345 kcal, 17.4 g of fat, 41.4 g of carbohydrates, and 5.2 g of protein, 1.3 g of fiber | 1 croissant, butter, jam, 1 cup of tea or coffee |
| **Nevirapine** | High-fat meal | Viramune prescribing information [47]  study design not specified | 24, healthy  12 males, 12 females  Race not specified | 200 | tablets | no significant changes in AUC, C_max,_ and t_max_ not specified | 857 kcal, 50 g of fat | not specified |
| **Rilpivirine** | High-fat meal | Crauwels et al. [48]  randomized, open-label, cross-over clinical trial | 20, healthy  18 males, 2 females  15 Caucasian, 1 African-American, 1 Asian, 3 Other | 75 | tablets | AUC ↑ 48%, C_max_ ↑ 65%, no significant changes in t_max_ | 928 kcal, 56 g of fat, 65 g of carbohydrates, and 42 g of protein | not specified |
|  | High-fat meal | Mehta et al. [49]  randomized, open-label, cross-over clinical trial | 24, healthy  Gender and race not specified | 25 | tablets | AUC ↑ 67%, C_max_ ↑ 114%, no significant changes in t_max_ | 900 kcal, 56 g of fat, 63 g of carbohydrates, and 38.3 g of protein | not specified |
|  | High-protein meal | Crauwels et al. [48]  randomized, open-label, cross-over clinical trial | 20, healthy  18 males, 2 females  15 Caucasian, 1 African-American, 1 Asian, 3 Other | 75 | tablets | AUC ↓ 16%, no significant changes in C_max_ and t_max_ | 300 kcal, 7.9 g of fat, 38.3 g of carbohydrates, and 18.8 g of protein | 240 mL of protein-rich drink |
|  | Standard meal | Crauwels et al. [48]  randomized, open-label, cross-over clinical trial | 20, healthy  18 males, 2 females  15 Caucasian, 1 African-American, 1 Asian, 3 Other | 75 | tablets | AUC ↑ 59%, C_max_ ↑ 74%, no significant changes in t_max_ | 533 kcal, 21 g of fat, 66.6 g of carbohydrates, and 20 g of protein | not specified |
|  | Standard meal | Custodio et al. [22]  randomized, open-label, cross-over clinical trial | 24, healthy  12 males, 12 females  22 Caucasian, 2 African-American | 25 | tablets | no significant changes in AUC and t_max_, C_max_ ↑ 20% | 540 kcal, 21 g of fat | 2 slices of white bread, 1 pat of butter, 1 slice of cheese, 2 slices of ham, 1 packet of jelly, 1 cup of decaffeinated coffee, 1 packet of sugar, and 1 cup of 2% milk |
|  | Standard meal | Mehta et al. [49]  randomized, open-label, cross-over clinical trial | 24, healthy  Gender and race not specified | 25 | tablets | AUC ↑ 57%, C_max_ ↑ 89%, no significant changes in t_max_ | 625 kcal, 22.2 g of fat, 75 g of carbohydrates, and 31.3 g of protein | not specified |
|  | Standard meal | Crauwels et al. [50]  randomized, open-label, cross-over clinical trial | 32, healthy  Gender and race not specified | 25 | dispersible tablets | AUC ↑ 45%, C_max_ ↑ 52%, t_max_ not specified | not specified | not specified |
|  | Moderate-fat meal | Lamorde et al. [51]  non-randomized, open-label, longitudinal clinical trial | 15, HIV (+)  Males and females  All Ugandan | 25 | tablets | AUC ↑ 19%, no significant changes in C_max_, t_max_ not specified | 589 kcal, 19 g of fat | a local Ugandan banana staple (matooke), tomatoes  and onions |
|  | Low-fat meal | Custodio et al. [22]  randomized, open-label, cross-over clinical trial | 24, healthy  12 males, 12 females  22 Caucasian, 2 African-American | 25 | tablets | no significant changes in AUC and t_max_, C_max_ ↑ 25% | 390 kcal, 12 g of fat | 2 slices of wheat bread, 1 tablespoon of Benecol Light Spread, 8 oz (237 mL) of 2% milk, and 4 oz (118 mL) of orange juice |
|  | Low-fat meal | Lamorde et al. [51]  non-randomized, open-label, longitudinal clinical trial | 15, HIV (+)  Males and females  All Ugandan | 25 | tablets | no significant changes in AUC and C_max_, t_max_ not specified | 353 kcal, 11 g of fat | a local Ugandan banana staple (matooke), tomatoes  and onions |

**Table S3. A detailed description of studies investigating interactions between INSTIs and food.**

| **Drug** | **Food type** | **Study details** | **Participants** | **Drug dose [mg]** | **Drug formulation** | **Observed effect** | **Quantitative meal composition** | **Qualitative meal composition** |
| --- | --- | --- | --- | --- | --- | --- | --- | --- |
| **Dolutegravir** | High-fat meal | Song et al. [52]  randomized, open-label, cross-over clinical trial | 24, healthy  10 males, 14 females  22 Caucasian, 1 African-American, 1 Arabic | 50 | tablets | AUC ↑ 66%, C_max_ ↑ 67%, t_max_ ↑ 3 h - all clinically irrelevant | 870 kcal, 51.2 g of fat | not specified |
|  | High-fat meal | Mehta et al. [49]  randomized, open-label, cross-over clinical trial | 24, healthy  Gender and race not specified | 50 | tablets | AUC ↑ 75%, C_max_ ↑ 73%, t_max_ ↑ 1.75 h | 900 kcal, 56 g of fat, 63 g of carbohydrates, and 38.3 g of protein | not specified |
|  | High-fat meal | Weller et al. [3]  randomized, open-label, cross-over clinical trial | 12, healthy  8 males, 4 females  7 Caucasian, 5 African-American | 50 | tablets | AUC ↑ 48%, C_max_ ↑ 37%, t_max_ not specified | 869 kcal, 51.2 g of fat | 2 eggs fried in butter, 2 strips of bacon, 2 slices of toast with butter, 4 oz (114 g) of hash brown potatoes, and 8 oz (237 mL) of whole milk |
|  | Standard meal | Song et al. [52]  randomized, open-label, cross-over clinical trial | 24, healthy  10 males, 14 females  22 Caucasian, 1 African-American, 1 Arabic | 50 | tablets | AUC ↑ 41%, C_max_ ↑ 52%, t_max_ ↑ 2 h - all clinically irrelevant | 600 kcal, 20 g of fat | not specified |
|  | Standard meal | Mehta et al. [49]  randomized, open-label, cross-over clinical trial | 24, healthy  Gender and race not specified | 50 | tablets | AUC ↑ 87%, C_max_ ↑ 75%, t_max_ ↑ 1.25 h | 625 kcal, 22.2 g of fat, 75 g of carbohydrates, and 31.3 g of protein | not specified |
|  | Low-fat meal | Song et al. [52]  randomized, open-label, cross-over clinical trial | 24, healthy  10 males, 14 females  22 Caucasian, 1 African-American, 1 Arabic | 50 | tablets | AUC ↑ 33%, C_max_ ↑ 46%, t_max_ ↑ 1 h - all clinically irrelevant | 300 kcal, 2.3 g of fat | not specified |
|  | Calcium carbonate | Song et al. [53]  randomized, open-label, cross-over clinical trial | 12, healthy  7 males, 5 females  8 Caucasian, 4 Other | 50 | tablets | AUC ↓ 39%, C_max_ ↓ 37%, t_max_ ↓ 1 h | 1200 mg of calcium carbonate, 480 mg of elemental calcium | not specified |
|  | Ferrous fumarate | Song et al. [53]  randomized, open-label, cross-over clinical trial | 11, healthy  6 males, 5 females  8 Caucasian, 3 Other | 50 | tablets | AUC ↓ 55%, C_max_ ↓ 58%, t_max_ ↑ 1 h | 324 mf of ferrous fumarate, 107 mg of elemental iron | not specified |
| **Elvitegravir** | High-protein meal | Shiomi et al. [19]  randomized, open-label, cross-over clinical trial | 11, healthy  All males  All Asian | 150 | tablets | AUC ↑116%, C_max_ ↑ 139%, no significant changes in t_max_ | 250 kcal, 8.8 g of fat, 34.3 g of carbohydrates, and 8.8 g of protein | 250 mL of Ensure protein-rich drink |
|  | High-protein meal | Yamada et al. [20]  randomized, open-label, cross-over clinical trial | 12, healthy  All males  All Asian | 150 | tablets | AUC ↑ 93%, C_max_ ↑ 142%, no significant changes in t_max_ | 250 kcal, 8.8 g of fat, 34.3 g of carbohydrates, and 8.8 g of protein | 250 mL of Ensure protein-rich drink |
|  | Standard meal | Shiomi et al. [19]  randomized, open-label, cross-over clinical trial | 11, healthy  All males  All Asian | 150 | tablets | AUC ↑ 94%, C_max_ ↑ 116%, no significant changes in t_max_ | 413 kcal, 9.6 g of fat, 72.2 g of carbohydrates, and 11.4 g of protein | 1 tuna, lettuce, and tomato sandwich, 1 ham, cheese, and egg sandwich, 1 cup of white peach jelly, and 160 g of apple juice |
|  | Standard meal | Yamada et al. [20]  randomized, open-label, cross-over clinical trial | 12, healthy  All males  All Asian | 150 | tablets | AUC ↑ 89%, C_max_ ↑ 113%, no significant changes in t_max_ | 413 kcal, 9.6 g of fat, 72.2 g of carbohydrates, and 11.4 g of protein | 2 slices of bread with strawberry jam, 1 boiled egg, and 160 g of grape juice |
|  | Milk (1) and apple juice (2) vs. high-protein meal (3) | Yonemura et al. [54]  randomized, open-label, cross-over clinical trial | 12, healthy  All males  All Asian | 150 | tablets | (1) compared to (3):  no significant changes AUC C_max_, and t_max_  (2) compared to (3):  AUC ↓ 61%, C_max_ ↓ 67%, no significant changes in t_max_ | (1): 137 kcal, 7.8 g of fat, 9.9 g of carbohydrates, and 6.8 g of protein  (2): 89 kcal, no fat and protein, 22.2 g of carbohydrates  (3): 250 kcal, 8.8 g of fat, 34.3 g of carbohydrates, and 8.8 g of protein | (1): 200 mL of milk  (2) 200 mL of apple juice  (3): 250 mL of Ensure protein-rich drink |
| **Raltegravir** | High-fat meal | Brainard et al. [55]  randomized, open-label, cross-over clinical trial | 20, healthy  10 males, 10 females  11 Caucasian, 6 Hispanic, 3 African-American | 800 | tablets | AUC ↑ 112%, C_max_ ↑ 96%, no significant changes in t_max_ | 825 kcal, 52 g of fat | 2 eggs, 2 strips of bacon, 4 oz hash browns, 2 slices of bread, 2 teaspoons of butter, 8 oz of whole milk |
|  | High-fat meal | Krishna et al. [56]  randomized, open-label, cross-over clinical trial | 17, healthy  Gender and race not specified | 1200 (2 x 600 mg) | tablets | no significant changes in AUC, C_max_ ↓ 28%, t_max_ ↑ 1.5 h | 997 kcal, 56 g of fat | 2 eggs, 20 g of butter, 2 strips of bacon, 113 g of hash brown potatoes, 2 slices of bread, 250 mL of whole milk |
|  | High-fat meal | Krishna et al. [56]  randomized, open-label, cross-over clinical trial | 17, healthy  Gender and race not specified | 1200 (3 x 400 mg) | tablets | AUC ↑ 39%, C_max_ ↓ 23%, no significant changes in t_max_ | 997 kcal, 56 g of fat | 2 eggs, 20 g of butter, 2 strips of bacon, 113 g of hash brown potatoes, 2 slices of bread, 250 mL of whole milk |
|  | High-fat meal | Wenning et al. [57]  randomized, open-label, cross-over clinical trial | 20, healthy  13 males, 7 females  Race not specified | 400 | tablets | no significant changes in AUC, C_max_ ↓ 35%, t_max_ ↑ 7.3 h | not specified | not specified |
|  | High-fat meal | Rhee et al. [58]  randomized, open-label, cross-over clinical trial | 12, healthy  9 males, 3 females  Race not specified | 400 | chewable tablets | no significant changes in AUC, C_max_ ↓ 62%, t_max_ ↑ 0.5 h | 825 kcal, 52 g of fat | not specified |
|  | Standard meal | Brainard et al. [55]  randomized, open-label, cross-over clinical trial | 20, healthy  10 males, 10 females  11 Caucasian, 6 Hispanic, 3 African-American | 800 | tablets | no significant changes in AUC, C_max,_ and t_max_ | 600 kcal, 21 g of fat | 4 slices of bread, 2 slices of American cheese, 2 slices of low‐fat ham, 8 oz (237 mL) of skim milk |
|  | Low-fat meal | Brainard et al. [55]  randomized, open-label, cross-over clinical trial | 20, healthy  10 males, 10 females  11 Caucasian, 6 Hispanic, 3 African-American | 800 | tablets | AUC ↓ 46%, C_max_ ↓ 52%, no significant changes in t_max_ | 300 kcal, 2.5 g of fat | 2 slices of bread, 2 packets of jelly, 8 oz (237 mL) of skim milk |
|  | Low-fat meal | Krishna et al. [56]  randomized, open-label, cross-over clinical trial | 18, healthy  Gender and race not specified | 1200 (2 x 600 mg) | tablets | AUC ↓ 41%, C_max_ ↓ 52%, no significant changes in t_max_ | 389 kcal, 27 g of fat | 2 slices of bread, 2 packets of strawberry jam, and 250 mL of skim milk |
|  | Low-fat meal | Krishna et al. [56]  randomized, open-label, cross-over clinical trial | 17, healthy  Gender and race not specified | 1200 (3 x 400 mg) | tablets | AUC ↓ 73%, C_max_ ↓ 75%, no significant changes in t_max_ | 389 kcal, 27 g of fat | 2 slices of bread, 2 packets of strawberry jam, and 250 mL of skim milk |
| **Bictegravir** | High-fat meal | Bictarvy prescribing information [16]  study design not specified | 12, healthy Gender and race not specified | 50 | tablets | AUC ↑ 24%, C_max_ ↑ 13% - all clinically irrelevant, t_max_ not specified | 800 kcal, 44.4 g of fat, 62.5 g of carbohydrates, and 37.5 g of protein | not specified |
|  | High-fat meal | Majeed et al. [18]  randomized, open-label, cross-over clinical trial | 52, healthy  Gender and race not specified | 30 | tablets | no significant changes in AUC and C_max_, t_max_ not specified | 1000 kcal, 50% of fat | not specified |
|  | Aluminum / magnesium hydroxide | Mathias et al. [59] | 14 of 42, healthy  10 males, 4 females  8 Caucasian, 6 African-American | 50 | tablets | AUC ↓ 79%, C_max_ ↓ 80%, t_max_ not specified | 1600 mg of aluminum hydroxide, 1600 mg of magnesium hydroxide | not applicable |
|  | Calcium carbonate | Mathias et al. [59] | 14 of 42, healthy  10 males, 4 females  8 Caucasian, 6 African-American | 50 | tablets | AUC ↓ 33%, C_max_ ↓ 42%, t_max_ not specified | 1200 mg of calcium carbonate | not applicable |
|  | Ferrous fumarate | Mathias et al. [59] | 14 of 42, healthy  10 males, 4 females  8 Caucasian, 6 African-American | 50 | tablets | AUC ↓ 63%, C_max_ ↓ 70%, t_max_ not specified | 324 mg of ferrous fumarate | not applicable |
| **Cabotegravir** | High-fat meal | Patel et al. [60]  randomized, open-label, cross-over clinical trial | 21 of 24, healthy  16 males, 8 females  16 Caucasian | 30 | tablets | no significant changes in AUC, C_max_, and t_max_ | 870 kcal, 51.2 g of fat | not specified |
|  | Not specified | Patel et al. [61]  randomized, open-label, parallel clinical trial | 15, healthy  Gender and race not specified | 10 | tablets | no significant changes in AUC and C_max_, t_max_ ↑ 1.5 h | not specified | not specified |
|  | Standard meal | Patel et al. [62]  randomized, open-label, cross-over clinical trial | 22, healthy  Gender and race not specified | 30 | tablets | no significant changes in AUC, C_max_, t_max_ not specified | 670 kcal, 22.3 g of fat | not specified |

**Table S4. A detailed description of studies investigating interactions between PIs and food.**

| **Drug** | **Food type** | **Study details** | **Participants** | **Drug dose [mg]** | **Drug formulation** | **Observed effect** | **Quantitative meal composition** | **Qualitative meal composition** |
| --- | --- | --- | --- | --- | --- | --- | --- | --- |
| **Amprenavir** | High-fat meal | Agenerase prescribing information [63]  study design not specified | 12, healthy  Gender and race not specified | 200 | capsules | AUC ↓ 21%, C_max_ ↓ 36%, t_max_ ↑ 0.5 h | 967 kcal, 67 g of fat, 58 g of carbohydrates, and 33 g of protein | not specified |
|  | High-fat meal | Sadler et al. [64]  randomized, open-label, cross-over clinical trial | 18, HIV (+)  15 males, 3 females  10 Caucasian, 7 African-American, 1 Other | 600 | capsules | AUC ↓ 14%, C_max_ ↓ 33%, t_max_ ↑ 0.75 h | 1000 kcal, 67 g of fat, 58 g of carbohydrates, and 33 g of protein | 2 slices of toasted white bread with butter, 2 eggs fried in butter, 2 slices of bacon, 2 oz (57 g) hash-browned potatoes, and a glass (237 mL) of whole milk |
|  | High-fat meal | Brouwers et al. [65]  non-randomized, cross-over clinical trial | 5, healthy  2 males, 3 females  Race not specified | 700 | tablets | AUC ↓ 60%, C_max_ ↓ 40%, t_max_ ↑ 2.5 h – all measured in duodenum | 600 kcal, 30.7 g of fat, 69 g of carbohydrates, and 12 g of protein | not specified |
|  | Low-fat meal | Falcoz et al. [66]  randomized, open-label, cross-over clinical trial | 24, healthy  All males  Race not specified | 1200 | capsules | AUC ↓ 23%, C_max_ ↓ 46%, t_max_ ↑ 1.25 h | 400 kcal, 11 g of fat, 69.3 g of carbohydrates, and 9.9 g of protein | cornflakes, semi-skimmed milk, and 2 slices of toasted white bread with margarine and marmalade |
| **Atazanavir** | High-fat meal | Reyataz prescribing information [67]  study design not specified | 12, healthy  Gender and race not specified | 400 | capsules | AUC ↑ 35%, no significant changes in C_max_ and t_max_ | 721 kcal, 37.3 g of fat, 29.4 g protein | not specified |
|  | High-fat meal | Reyataz prescribing information [67]  study design not specified | 12, healthy  Gender and race not specified | 300 | capsules | no significant changes in AUC, C_max_ ↑ 11%, t_max_ ↑ 2.5 h | 951 kcal, 54.7 g of fat, 35.9 g of protein | not specified |
|  | High-fat meal | Sevinsky et al. [68]  randomized, open-label, cross-over clinical trial | 64, healthy  40 males, 24 females  29 Caucasian, 33 African-American, 2 Asian | 300 | tablets | no significant changes in AUC and C_max_, t_max_ ↑ 1.5 h | 1038 kcal, 59 g of fat | 2 slices of white bread, butter, jam, 2 fried eggs, 3 strips of bacon, 113 g of hashed brown potatoes, and 237 mL of whole milk |
|  | Light meal | Reyataz prescribing information [67]  study design not specified | 12, healthy  Gender and race not specified | 400 | capsules | AUC ↑ 70%, C_max_ ↑ 57%, t_max_ not specified | 357 kcal, 8.2 g of fat, 10.6 g of carbohydrates, and 37.3 g of protein | not specified |
|  | Light meal | Reyataz prescribing information [67]  study design not specified | 12, healthy  Gender and race not specified | 300 | capsules | AUC ↑ 33%, C_max_ ↑ 40%, t_max_ not specified | 336 kcal, 5.1 g of fat, 9.3 g of protein | not specified |
|  | Light meal | Sevinsky et al. [68]  randomized, open-label, cross-over clinical trial | 64, healthy  40 males, 24 females  29 Caucasian, 33 African-American, 2 Asian |  | tablets | AUC ↑ 28%, C_max_ ↑ 42%, t_max_ ↑ 0.5 h | 336 kcal, 5.1 g of fat | 2 slices of white bread, margarine, jam, 148 mL of apple juice, 148 mL of skimmed milk |
| **Darunavir** | High-fat meal | Sekar et al. [69]  randomized, open-label, cross-over clinical trial | 24, healthy  12 males, 12 females  All Caucasian | 400 | tablets | AUC ↑ 47%, C_max_ ↑ 64%, t_max_ ↑ 1.5 h | 928 kcal, 56 g of fat, 65 g of carbohydrates, 41 g of protein | 2 eggs fried in butter, 2 strips of bacon, 2 slices of white bread with butter, 1 croissant with 1 slice of cheese, and 240 mL of whole milk |
|  | High-fat meal | Crauwels et al. [15]  randomized, open-label, cross-over clinical trial | 24, healthy  12 males, 12 females  All Caucasian | 800 | tablets | AUC ↑ 31%, C_max_ ↑ 62%, t_max_ ↑ 2 h | 928 kcal, 56 g of fat, 65 g of carbohydrates, 41 g of protein | 2 eggs fried in butter, 2 strips of bacon, 2 slices of white bread with butter, 1 croissant with 1 slice of cheese, and 240 mL of whole milk |
|  | High-fat meal | Kakuda et al. [70]  randomized, open-label, cross-over clinical trial | 19, healthy  11 males, 8 females  18 Caucasian, 1 Other | 800 | tablets | AUC ↑ 73%, C_max_ ↑ 122%, t_max_ ↑ 1.5 h | 928 kcal, 56 g of fat, 65 g of carbohydrates, 41 g of protein | 2 scrambled eggs, 114 g of hashed brown potatoes, 2 strips of bacon, 2 slices of bread, butter, jelly, and 240 mL of whole milk |
|  | High-protein meal | Sekar et al. [69]  randomized, open-label, cross-over clinical trial | 24, healthy  12 males, 12 females  All Caucasian | 400 | tablets | AUC ↑ 72%, C_max_ ↑ 54%, t_max_ ↑ 1.5 h | 250 kcal, 8.4 g of fat, 33.4 g of carbohydrates, 10.5 g of protein | Ensure 250 mL chocolate or vanilla |
|  | Standard meal | Sekar et al. [69]  randomized, open-label, cross-over clinical trial | 24, healthy  12 males, 12 females  All Caucasian | 400 | tablets | AUC ↑ 54%, C_max_ ↑ 48%, t_max_ ↑ 1.5 h | 533 kcal, 21 g of fat, 67 g of carbohydrates, 19 g of protein | 4 slices of bread, 1 slice of ham, 1 slice of cheese, butter, jelly, and 2 cups of coffee/tea with milk and/or sugar |
|  | Standard meal | Kakuda et al. [71]  randomized, open-label, cross-over clinical trial | 32, healthy  22 males, 10 females  All Caucasian | 800 (2 formulations compared) | tablets | AUC ↑ 53-91%, C_max_ ↑ 32-58%, t_max_ ↑ 1-1.5 h | 533 kcal, 21 g of fat, 67 g of carbohydrates, 19 g of protein | 4 slices of bread, 2 slices of ham and/or cheese, butter, jelly, and 2 cups of decaffeinated coffee or tea with milk and/or sugar |
|  | Standard meal | Kakuda et al. [71]  randomized, open-label, cross-over clinical trial | 128, healthy  82 males, 46 females  127 Caucasian, 1 African-American | 800 (2 formulations compared) | tablets | no significant changes in AUC, C_max_ ↑ 38-45%, t_max_ ↑ 1 h | 533 kcal, 21 g of fat, 67 g of carbohydrates, 19 g of protein | 4 slices of bread, 2 slices of ham and/or cheese, butter, jelly, and 2 cups of decaffeinated coffee or tea with milk and/or sugar |
|  | Standard meal | Kakuda et al. [70]  randomized, open-label, cross-over clinical trial | 114, healthy  63 males, 51 females  113 Caucasian, 1 Other | 800 | tablets (2 formulations compared) | AUC ↑ 67-70%, C_max_ ↑ 119-123%, t_max_ ↑ 1 h | 533 kcal, 21 g of fat, 67 g of carbohydrates, 19 g of protein | 4 slices of bread, 2 slices of ham and/or cheese, butter, jelly, and 2 cups (up to 480 mL) of decaffeinated coffee or tea with milk and/or sugar |
|  | Standard meal | Kakuda et al. [72]  randomized, open-label, cross-over clinical trial | 17, healthy  Race and gender not specified | 600 | oral suspension | no significant changes in AUC and C_max_, t_max_ ↑ 2 h | 533 kcal, 21 g of fat, 67 g of carbohydrates, 19 g of protein | 4 slices of bread, 2 slices of ham and/or cheese, butter, jelly, and 2 cups (up to 480 mL) of decaffeinated coffee or tea with milk and/or sugar |
|  | Low-fat meal | Sekar et al. [69]  randomized, open-label, cross-over clinical trial | 24, healthy  12 males, 12 females  All Caucasian | 400 | tablets | AUC ↑ 64%, C_max_ ↑ 49%, t_max_ ↑ 1.5 h | 240 kcal, 12 g of fat, 28 g of carbohydrates, 5 g of protein | croissant with coffee |
|  | Garlic | Cloarec et al. [73]  case studies | 2, HIV (+)  1 male  1 female | 800, 4x day  600, 2x day | tablets  tablets | subtherapeutic C_throuh_ (after garlic eviction - C_throuh_ ↑ 3 times higher)  subtherapeutic C_throuh_ (after garlic eviction - C_throuh_ ↑ 2 times higher) | not specified  not specified | 15 cloves of garlic per week  not specified |
| **Fosamprenavir** | High-fat meal | Brouwers et al. [65]  non-randomized, cross-over clinical trial | 5, healthy  2 males, 3 females  Race not specified | 700 | tablets | In stomach: AUC ↓ 49%, C_max_ ↓ 50%, t_max_ ↑ 2 h  In duodenum: AUC ↓ 33%, C_max_ ↓ 26%, t_max_ ↑ 2 h | 600 kcal, 30.7 g of fat, 69 g of carbohydrates, 12 g of protein | 300 mL of nutritional drink (Scandishake Mix) |
|  | High-fat meal | Lexiva prescribing information [74]  study design not specified | 12, healthy  Gender and race not specified | 1400 | tablets | no significant changes in AUC, C_max_, and t_max_ of amprenavir | 967 kcal, 67 g of fat, 58 g of carbohydrates, 33 g of protein | not specified |
|  | High-fat meal | Lexiva prescribing information [74]  study design not specified | 12, healthy  Gender and race not specified | 1400 | oral suspension | AUC ↓ 28%, C_max_ ↓ 46%, t_max_ ↑ 0.72 h | 967 kcal, 67 g of fat, 58 g of carbohydrates, 33 g of protein | not specified |
| **Indinavir** | High-fat high-protein meal | Yeh et al. [75]  randomized, double-blind, placebo-controlled clinical trial | 12, healthy  All males  Race not specified | 400 | capsules | AUC ↓ 78%, C_max_ ↓ 86%, t_max_ ↑ 1.3 h | 784 kcal, 48.6 g of fat, 57.2 g of carbohydrates, 31.3 g of protein | 2 slices of toast, 2 scrambled eggs, 2 pats of butter, 2 strips of bacon, 4 oz (114 g) of hash brown potatoes, 8 oz (237 mL) of whole milk |
|  | High-fat meal | Carver et al. [76]  randomized, cross-over clinical trial | 7, HIV (+)  All males  5 Caucasian, 2 African-American | 600 | capsules | AUC ↓ 33%, C_max_ ↓ 49%, t_max_ ↑ 1 h | 680 kcal, 75.6 g of fat | 500 mL of Microlipi |
|  | High-protein meal | Carver et al. [76]  randomized, cross-over clinical trial | 7, HIV (+)  All males  5 Caucasian, 2 African-American | 600 | capsules | AUC ↓ 68%, C_max_ ↓ 76%, t_max_ ↑ 2.75 h | 680 kcal, 95.2 g of carbohydrates, 68 g of protein | 500 mL of Promod |
|  | High-carbohydrate meal | Carver et al. [76]  randomized, cross-over clinical trial | 7, HIV (+)  All males  5 Caucasian, 2 African-American | 600 | capsules | AUC ↓ 45%, C_max_ ↓ 62%, t_max_ ↑ 2.5 h | 680 kcal, 181.3 g of carbohydrates | 500 mL of Moducal |
|  | Low-fat meal | Yeh et al. [75]  randomized, open-label, cross-over clinical trial | 12, healthy  8 males, 4 females  Race not specified | 800 | capsules | no significant changes in AUC, C_max_, and t_max_ | 292 kcal, 2.1 g of fat, 63.5 g of carbohydrates, 5.4 g of protein | 2 slices of toast, 2 tablespoons of jelly, 6 oz (178 mL) of apple juice, 1 cup of coffee, 2 tablespoons of skim milk, 2 tablespoons of sugar |
|  | Low-calorie meal | Yeh et al. [75]  randomized, open-label, cross-over clinical trial | 12, healthy  8 males, 4 females  Race not specified | 800 | capsules | no significant changes in AUC, C_max_, and t_max_ | 141 kcal, 1 g of fat, 28.8 g of carbohydrates, 5.7 g of protein | cornflakes (21.1 g), 1 tablespoon of sugar, 0.5 cup of skim milk |
|  | Low-calorie meal | Aarnoutse et al. [77]  randomized, open-label, cross-over clinical trial | 9, HIV (+)  All males  Race not specified | 800 | capsules | AUC ↓ 12%, C_max_ ↓ 19%, t_max_ ↑ 0.5 h | 339 kcal, 12.1 g of fat, 45.8 g of carbohydrates, 11.9 g of protein | 2 filled bread  rolls and 130 mL of water, coffee, or tea |
|  | High-fat meal (1) vs. low-fat meal (2) | Saah et al. [78]  randomized, double blind, placebo controlled, parallel clinical trial | 10 of 53, healthy  Gender not specified  33 Caucasian, 19 African-American, 1 Asian | 800-1600 | capsules | (1) compared to (2):  AUC ↓ 16% or less, C_max_ ↓ 23% or less, t_max_ not specified | not specified | (1): 2 scrambled eggs, 2 strips of bacon, 2 slices of toast, 2 pats of butter, 4 oz. of hash brown potatoes, and 8 oz. of whole milk  (2): 2 slices of toast, 2 teaspoons of jelly, 6 oz (178 mL) of apple juice, 1 cup of coffee, 2 tablespoons of skim milk, 2 teaspoons of sugar |
| **Lopinavir** | High-fat meal | Kaletra prescribing information [79]  study design not specified | 12, healthy  Gender and race not specified | 400 | capsules | AUC ↑ 97%, C_max_ ↑ 43%, t_max_ not specified | 872 kcal, 54.3 g of fat | not specified |
|  | High-fat meal | Kaletra prescribing information [79]  study design not specified | 12, healthy  Gender and race not specified | 400 | oral solution | AUC ↑ 130%, C_max_ ↑ 56%, t_max_ not specified | 872 kcal, 54.3 g of fat | not specified |
|  | High-fat meal | Klein et al. [80]  randomized, open-label, cross-over clinical trial | 126, healthy  92 males, 34 females  94 Caucasian, 19 African-American, 14 Hispanic | 400 | tablets | AUC ↑ 19%, no significant changes in C_max_, t_max_ not specified | 1000 kcal, 55.6 g of fat | not specified |
|  | High-fat meal | Lamorde et al. [81]  non-randomized, open-label, cross-over clinical trial | 12, HIV (+)  All males  All African-American | 400 | tablets | AUC ↓ 14%, C_max_ ↓ 14%, t_max_ not specified | 840 kcal, 36 g of fat | bread, margarine, sausages and tea with milk |
|  | Medium-fat meal | Kaletra prescribing information [79]  study design not specified | 12, healthy  Gender and race not specified | 400 | capsules | AUC ↑ 48%, C_max_ ↑ 23%, t_max_ not specified | 591 kcal, 15.8 g of fat | not specified |
|  | Medium-fat meal | Klein et al. [80]  randomized, open-label, cross-over clinical trial | 126, healthy  92 males, 34 females  94 Caucasian, 19 African-American, 14 Hispanic | 400 | tablets and capsules | tablets: AUC ↑ 27%, Cmax ↑ 18%, t_max_ not specified  capsules: AUC ↑ 62%, C_max_ ↑ 32%, t_max_ not specified | 550 kcal, 15.3 g of fat | not specified |
|  | Medium-fat meal | Kaletra prescribing information [79]  study design not specified | 12, healthy  Gender and race not specified | 400 | oral solution | AUC ↑ 80%, C_max_ ↑ 54%, t_max_ not specified | 591 kcal, 15.8 g of fat | not specified |
|  | Moderate-fat meal | Lamorde et al. [81]  non-randomized, open-label, cross-over clinical trial | 12, HIV (+)  All males  All African-American | 400 | tablets | no significant changes in AUC and C_max_, t_max_ not specified | 840 kcal, 36 g of fat | bread, margarine, sausages and tea with milk |
|  | Low-fat meal | Oki et al. [82]  non-randomized, cross-over clinical trial | 8, healthy  6 males, 2 females  All Asian | 400 | capsules | no significant changes in AUC and C_max_, t_max_ ↑ 2.4 h | 530 kcal, 20 g of fat | not specified |
|  | Low-fat meal | Oki et al. [82]  non-randomized, cross-over clinical trial | 12, healthy  Gender not specified  All Caucasian | 400 | capsules | AUC ↑ 72%, C_max_ ↑ 38%, t_max_ ↑ 2.1 h | 530 kcal, 20 g of fat | not specified |
|  | Not specified | Kanter et al. [83]  randomized, open-label, cross-over clinical trial | 12, healthy  8 males, 4 females  All Caucasian | 400 | tablets and granules | tablets: no significant changes in AUC and Cmax, t_max_ ↑ 1 h  granules: AUC ↑ 51%, C_max_ ↑ 39%, t_max_ ↑ 3 h | not specified | not specified |
| **Nelfinavir** | High-fat meal | Viracept prescribing information [84] study design not specified | 23, healthy  Gender and race not specified | 1250 | tablets | AUC ↑ 520%, C_max_ ↑ 330%, t_max_ ↑ 2 h | 1000 kcal, 55.6 g of fat, 78.1 g of carbohydrates, 46.9 g of protein | not specified |
|  | Standard meal | Viracept prescribing information [84] study design not specified | 22, healthy  Gender and race not specified | 1250 | tablets | AUC ↑ 510%, C_max_ ↑ 380%, t_max_ ↑ 2.1 h | 500 kcal, 27.8 g of fat | not specified |
|  | Standard meal | Kaeser et al. [85]  randomized, cross-over clinical trial | 50, healthy  All males  Race not specified | 1250 | tablets (2 formulations compared) | AUC ↑ 600-800%, C_max_ and t_max_ not specified | 820 kcal | not specified |
|  | Low-fat meal | Viracept prescribing information [84] study design not specified | 22, healthy  Gender and race not specified | 1250 | tablets | AUC ↑ 310%, C_max_ ↑ 230%, t_max_ ↑ 2 h | 500 kcal, 11.1 g of fat | not specified |
|  | Very low-fat meal | Viracept prescribing information [84] study design not specified | 21, healthy  Gender and race not specified | 1250 | tablets | AUC ↑ 220%, C_max_ ↑ 200%, t_max_ ↑ 1 h | 125 kcal, 3.5 g of fat | not specified |
|  | Low-fat meal (1) vs. high-fat meal (2) | Aarnoutse et al. [86]  randomized, open-label, parallel clinical trial | 20 of 27, healthy  15 males, 12 females  All Caucasian | 2000-2500 | tablets | (1) compared to (2):  AUC ↓ 25-47%, C_max_ ↓ 14-43% | (1) 271 kcal, 11 g of fat, 26.4 g of carbohydrates, 16 g of protein  (2) 610 kcal, 22.4 g of fat, 77.8 g of carbohydrates, 24.4 g of protein | (1) 1 slice of bread with butter and cheese, and 130 mL of  semi-skimmed milk  (2) 4 slices of bread, filled with butter and cheese, ham, paste  or jam, 130 mL of water |
|  | Standard meal (1) vs. low-fat meal (2) | Kurowski et al. [87]  non-randomized, open-label, cross-over clinical trial | 24, healthy  All males  Race not specified | 2500 | tablets | (1) compared to (2):  AUC ↓ 13%, no significant changes in C_max_, t_max_ not specified | (1): 800 kcal  (2): 350 kcal | not specified  not specified |
| **Ritonavir** | High-fat meal | Ng et al. [88]  randomized, open-label, cross-over clinical trial | 25, healthy  Gender and race not specified | 100 | tablets | AUC ↓ 24%, C_max_ ↓ 26%, t_max_ ↑ 1.5 h | 50% of fat | not specified |
|  | High-fat meal | Norvir prescribing information [89]  study design not specified | 12, healthy  Gender, and race not specified | 100 | tablets | AUC ↓ 22%, C_max_ ↓ 22%, t_max_ not specified | 917 kcal, 61.1 g of fat | not specified |
|  | High-fat meal | Klein et al. [80]  randomized, open-label, cross-over clinical trial | 126, healthy  92 males, 34 females  94 Caucasian, 19 African-American, 14 Hispanic | 100 | tablets | AUC ↑ 24%, C_max_ ↑ 10%, t_max_ not specified | 1000 kcal, 55.6 g of fat | not specified |
|  | High-fat meal | Lamorde et al. [81]  non-randomized, open-label, cross-over clinical trial | 12, HIV (+)  All males  All African-American | 100 | tablets | AUC ↓ 29%, C_max_ ↓ 29%, t_max_ not specified | 840 kcal, 36 g of fat | bread, margarine, sausages and tea with milk |
|  | High-fat meal | Norvir prescribing information [89]  study design not specified | 12, healthy  Gender and race not specified | 100 | oral powder | AUC ↓ 36%, C_max_ ↓ 36%, t_max_ not specified | 917 kcal, 61.1 g of fat | not specified |
|  | High-fat meal | Salem et al. [90]  randomized, open-label, cross-over clinical trial | 48, healthy  30 males, 18 females  30 Caucasian, 13 African-American, 2 Asian, 3 Other | 100 | oral powder | AUC ↓ 32%, C_max_ ↓ 49%, t_max_ not specified | 900 kcal, 60 g of fat | not specified |
|  | Standard meal | Norvir prescribing information [89]  study design not specified | 12, healthy  Gender and race not specified | 600 | oral solution | no significant changes in AUC, C_max_ ↓ 23%, t_max_ not specified | 514 kcal, 5.7 g of fat | not specified |
|  | Standard meal | Norvir prescribing information [89]  study design not specified | 12, healthy  Gender and race not specified | 600 | capsules | no significant changes in AUC and C_max_, t_max_ not specified | 615 kcal, 9.9 g of fat, 116.9 g of carbohydrates, 13.8 g of protein | not specified |
|  | Standard meal | Norvir prescribing information [89]  study design not specified | 12, healthy  Gender, and race not specified | 100 | tablets | AUC ↓ 22%, C_max_ ↓ 22%, t_max_ not specified | 857 kcal, 28.6 g of fat | not specified |
|  | Standard meal | Kakuda et al. [71]  randomized, open-label, cross-over clinical trial | 32, healthy  22 males, 10 females  All Caucasian | 100 (2 formulations compared) | tablets | AUC ↓ 24-28%, C_max_ ↓ 44%, t_max_ ↑ 1.25-2.25 h | 533 kcal, 21 g of fat, 67 g of carbohydrates, 19 g of protein | 4 slices of bread, 2 slices of ham and/or cheese, butter, jelly, and 2 cups of decaffeinated coffee or tea with milk and/or sugar |
|  | Standard meal | Kakuda et al. [71]  randomized, open-label, cross-over clinical trial | 128, healthy  82 males, 46 females  127 Caucasian, 1 African-American | 100 (2 formulations compared) | tablets | AUC ↓ 10-18%, C_max_ ↓ 22%, t_max_ ↑ 1.5 h | 533 kcal, 21 g of fat, 67 g of carbohydrates, 19 g of protein | 4 slices of bread, 2 slices of ham and/or cheese, butter, jelly, and 2 cups of decaffeinated coffee or tea with milk and/or sugar |
|  | Standard meal | Norvir prescribing information [89]  study design not specified | 12, healthy  Gender, and race not specified | 100 | oral powder | AUC ↓ 36%, C_max_ ↓ 36%, t_max_ not specified | 617 kcal, 19.9 g of fat | not specified |
|  | Medium-fat meal | Ng et al. [88]  randomized, open-label, cross-over clinical trial | 26, healthy  Gender and race not specified | 100 | tablets | AUC ↓ 17%, C_max_ ↓ 22%, t_max_ ↑ 1 h | 25% of fat | not specified |
|  | Medium-fat meal | Klein et al. [80]  randomized, open-label, cross-over clinical trial | 126, healthy  92 males, 34 females  94 Caucasian, 19 African-American, 14 Hispanic | 100 | tablets and capsules | tablets: AUC ↑ 15%, no significant changes in C_max_, t_max_ not specified  capsules: AUC ↑ 40%, C_max_ ↑ 28%, t_max_ not specified | 550 kcal, 15.3 g of fat | not specified |
|  | Medium-fat meal | Lamorde et al. [81]  non-randomized, open-label, cross-over clinical trial | 12, HIV (+)  All males  All African-American | 100 | tablets | no significant changes in AUC and C_max_, t_max_ not specified | 840 kcal, 36 g of fat | bread, margarine, sausages and tea with milk |
|  | Medium-fat meal | Salem et al. [90]  randomized, open-label, cross-over clinical trial | 48, healthy  30 males, 18 females  30 Caucasian, 13 African-American, 2 Asian, 3 Other | 100 | oral powder | AUC ↓ 23%, C_max_ ↓ 39%, t_max_ not specified | 600 kcal, 20 g of fat | not specified |
|  | High-fat meal (1) vs. standard meal (2) | Veldkamp et al. [91]  randomized, open-label, cross-over clinical trial | 6, HIV (+)  All males  Race not specified | 200 | soft gel capsules | (1) compared to (2):  no significant changes in AUC and C_max,_  t_max_ not specified | (1): 931 kcal, 50 g of fat  (2): 528 kcal, 21 g of fat | not specified |
|  | High-fat meal (1) vs. low-fat meal (2) | Saah et al. [78]  randomized, double blind, placebo controlled, parallel clinical trial | 10 of 53, healthy  Gender not specified  33 Caucasian, 19 African-American, 1 Asian | 200-800 | capsules | (1) compared to (2):  AUC ↓ 32% or less, C_max_ ↓ 21-43%, t_max_ not specified | not specified | (1): 2 scrambled eggs, 2 strips of bacon, 2 slices of toast, 2 pats of butter, 4 oz (57 g) of hash brown potatoes, and 8 oz (237 mL) of whole milk  (2): 2 slices of toast, 2 teaspoons of jelly, 6 oz (178 mL) of apple juice, 1 cup of coffee, 2 tablespoons of skim milk, 2 teaspoons of sugar |
|  | Low-calorie meal | Aarnoutse et al. [77]  randomized, open-label, cross-over clinical trial | 9, HIV (+)  All males  Race not specified | 100 | capsules | AUC ↓ 17%, C_max_ ↓ 20%, t_max_ ↑ 0.5 h | 339 kcal, 12.1 g of fat, 45.8 g of carbohydrates, 11.9 g of protein | 2 filled bread rolls and 130 mL of water, coffee, or tea |
|  | Low-fat meal (1) vs. high-fat meal (2) | Aarnoutse et al. [86]  randomized, open-label, parallel clinical trial | 20 of 27,  15 males, 12 females  All Caucasian | 200-400 | capsules | (1) compared to (2):  AUC ↓ 8-48%, C_max_ ↓ 5-51% | (1) 271 kcal, 11 g of fat, 26.4 g of carbohydrates, 16 g of protein  (2) 610 kcal, 22.4 g of fat, 77.8 g of carbohydrates, 24.4 g of protein | (1) 1 slice of bread with butter and cheese, and 130 mL of  semi-skimmed milk  (2) 4 slices of bread, filled with butter and cheese, ham, paste  or jam, 130 mL of water |
|  | Pudding | Salem et al. [90]  randomized, open-label, cross-over clinical trial | 48, healthy  30 males, 18 females  30 Caucasian, 13 African-American, 2 Asian, 3 Other | 100 | oral powder | no significant changes in AUC and C_max_, t_max_ not specified | not specified | not specified |
|  | Infant formula | Salem et al. [90]  randomized, open-label, cross-over clinical trial | 24, healthy  15 males, 9 females  19 Caucasian, 5 African-American | 100 | oral powder | no significant changes in AUC and C_max_, t_max_ not specified | not specified | not specified |
|  | Apple sauce | Salem et al. [90]  randomized, open-label, cross-over clinical trial | 24, healthy  15 males, 9 females  19 Caucasian, 5 African-American | 100 | oral powder | no significant changes in AUC and C_max_, t_max_ not specified | not specified | not specified |
|  | Garlic | Gallicano et al. [92]  randomized, open-label, cross-over clinical trial | 10, healthy  5 males, 5 females  All Caucasian | 400 | capsules | no significant changes in AUC, C_max_, and t_max_ | 20 mg daily - equivalent to 2 g of fresh garlic | dietary supplement Odourless Garlic by Life Brand |
|  | Not specified | Kanter et al. [83]  randomized, open-label, cross-over clinical trial | 12, healthy  8 males, 4 females  All Caucasian | 100 | tablets and granules | tablets: no significant changes in AUC, C_max_, and t_max_  granules: AUC ↑ 54%, C_max_ ↑ 100%, t_max_ ↑ 3 h | not specified | not specified |
| **Saquinavir** | High-fat meal | Invirase prescribing information [93]  study design not specified | 6, healthy  Gender and race not specified | 600 | not specified | AUC ↑ 571%, C_max_ and t_max_ not specified | 1006 kcal, 57 g of fat, 60 g of carbohydrates, 48 g of protein | not specified |
|  | High-fat meal | Kenyon et al. [94]  randomized, open-label, cross-over clinical trial | 8, healthy  4 males, 4 females  Race not specified | 600 | capsules | AUC ↑ 625%, C_max_ ↑ 435%, t_max_ ↑ 3.25 h | 1300 kcal | 1 bowl of cornflakes with 100 mL of whole milk, 2 rashers of lean bacon, 2 fried eggs, 2 slices of toast with butter, 150 mL of decaffeinated tea or coffee |
|  | High-fat meal (1) vs. standard meal (2) | Hugen et al. [95]  non-randomized clinical trial | 6, HIV (+)  All males  Race not specified | 1200 | soft gel capsules | (1) compared to (2): AUC ↑ 93%, C_max_ ↑ 80%, no significant changes in t_max_ | (1): 1040 kcal,  62 g of fat, 80.6 g of carbohydrates, 39 g of protein  (2): 600 kcal, 22 g of fat, 76.5 g of carbohydrates, 24 g of protein | (1): 1 slice of white bread, 1 slice of brown bread, 1 croissant, 3 pats of margarine, 15 g of jam, 1 slice of Gouda cheese, 1 fried egg, 2 strips of bacon and 260 mL of whole milk  (2): 2 slices of white bread, 2 slices of brown bread, 4 pats of low-fat margarine, 15 g of jam, 15 g of liver sausage, 1 slice of Gouda cheese, 1 slice of ham and 130 mL of tea or coffee |
|  | High-fat meal (1) vs. standard meal (2) | Veldkamp et al. [91]  randomized, open-label, cross-over clinical trial | 6, HIV (+)  All males  Race not specified | 2000 | soft gel capsules | (1) compared to (2): no significant changes in AUC and C_max,_  t_max_ not specified | (1): 931 kcal, 50 g of fat  (2): 528 kcal, 21 g of fat | not specified |
|  | Garlic | Piscitelli et al. [96]  non-randomized, longitudinal clinical trial | 10, healthy  4 males, 6 females  Race not specified | 1200 | capsules | AUC ↓ 51%, C_max_ ↓ 54%, t_max_ ↑ 1 h | 2 capsules daily - 1 g of garlic extract, 1.5 mg of allicin | dietary supplement GarliPure |
| **Tipranavir** | Standard meal | Aptivus prescribing information [97]  study design not specified | 12, healthy  Gender and race not specified | not specified | capsules | no significant changes in AUC and C_max_, t_max_ not specified | 591 kcal, 16 g of fat | not specified |
|  | Standard meal | Aptivus prescribing information [97]  study design not specified | 12, healthy  Gender and race not specified | not specified | oral solution | no significant changes in AUC and C_max_, t_max_ not specified | 591 kcal, 16 g of fat | not specified |

**Table S5. A detailed description of studies investigating the effect of food on maraviroc and fostemsavir bioavailability.**

| Drug | Food type | Study details | Participants | Drug dose [mg] | Drug formulation | Observed effect | Quantitative meal composition | Qualitative meal composition |
| --- | --- | --- | --- | --- | --- | --- | --- | --- |
| Maraviroc | High-fat meal | Selzentry prescribing information [98]  study design not specified | 12, healthy  gender and race not specified | 300 | tablets | AUC ↓ 33%, C_max_ ↓ 33%, t_max_ not specified | 900 kcal, 55.6 g of fat, 62.5 g of carbohydrates, and 37.5 g of protein | not specified |
|  | High-fat meal | Selzentry prescribing information [98]  study design not specified | 12, healthy  gender and race not specified | 75 | solution | AUC ↓ 73%, C_max,_ and t_max_ not specified | 900 kcal, 55.6 g of fat, 62.5 g of carbohydrates, and 37.5 g of protein | not specified |
|  | Not specified | Fätkenheuer et al. [99]  randomized, placebo-controled clinical trial | 16, HIV (+)  gender and race not  specified | 300 | tablets | AUC ↓ 50%, C_max_ ↓ 60%, no significant changes in t_max_ | not specified | not specified |
| Fostemsavir | High-fat meal | Rukobia prescribing information [100]  study design not specified | 12, healthy  gender and race not specified | not specified | extended-release tablets | AUC ↑ 81% - considered clinically insignificant, no significant changes in C_max_, t_max_ not specified | 985 kcal, 66 g of fat | not specified |
|  | Standard meal | Rukobia prescribing information [100]  study design not specified | 12, healthy  gender and race not specified | not specified | extended-release tablets | no significant changes in AUC and C_max_, t_max_ not specified | 423 kcal, 17 g of fat | not specified |

**Table S6. A detailed description of studies investigating interactions between antiretroviral drugs and juices.**

| **Drug** | **Juice type** | **Study details** | **Juice volume [mL]** | **Participants** | **Drug dose [mg]** | **Drug formulation** | **Observed effect** |
| --- | --- | --- | --- | --- | --- | --- | --- |
| **Delavirdine** | regular orange juice | Shelton et al. [101]  randomized, open-label, cross-over clinical trial | 177 (6 oz) | 21, HIV (+), 11 with gastric hypoacidity  19 males, 2 females  2 Caucasian, 8 African-American, 11 Hispanic | 400 | tablets | in patients without gastric hypoactivity:  AUC ↑ 14%, no significant changes in C_max_  in patients with gastric hypoactivity:  AUC ↑ 57%, C_max_ ↑ 53% |
| **Amprenavir** | grapefruit juice | Demarles et al. [102]  randomized, open-label, cross-over clinical trial | 200 | 12, healthy  6 males, 6 females  Race not specified | 1200 | capsules | no significant changes in AUC, C_max_ ↓ 22%, t_max_ delayed by 0.4 h |
| **Indinavir** | grapefruit juice | Shelton et al. [103]  randomized, open-label, cross-over clinical trial | 180 | 14, HIV (+)  10 males, 4 females  7 Caucasian, 5 African-American, 2 Hispanic | 800 | capsules | no significant changes in AUC, C_max_, and t_max_ |
|  | grapefruit juice | Penzak et al. [104]  non-randomized, open-label, cross-over clinical trial | 227,3 | 13, healthy  12 males, 1 female  Race not specified | 800 | capsules | no significant changes in AUC, C_max_, and t_max_ |
|  | Seville orange juice | Penzak et al. [104]  non-randomized, open-label, cross-over clinical trial | 227,3 | 13, healthy,  12 males, 1 female  Race not specified | 800 | capsules | no significant changes in AUC and C_max_, t_max_ delayed by 0.6 h |
| **Saquinavir** | grapefruit juice | Kupferschmidt et al. [105]  non-randomized, open-label, cross-over clinical trial | 200 | 8, healthy  All males  Race not specified | 600 | capsules | AUC ↑ 50%, C_max_ ↑ 93%, no significant changes in t_max_ |
|  |  |  |  |  |  |  |  |

**Table S7. A detailed description of studies assessing interactions between antiretroviral drugs and alcohol.**

| **Drug** | **Study details** | **Participants** | **Drug dose [mg]** | **Drug formulation** | **Alcohol dose [g/kg b.w.]** | **The effect on drug pharmacokinetics** | **The effect on ethanol pharmacokinetics** |
| --- | --- | --- | --- | --- | --- | --- | --- |
| **Maraviroc** | Gruber et al. [106]  randomized, double-blind, placebo-controlled clinical trial | 10, healthy  8 males, 2 females  9 Caucasian, 1 African-American | 300 | not specified | 1 | no significant changes in AUC, C_max_, t_max,_ and t_1/2_ | AUC ↑ 12%, no significant changes in C_max_ and t_max_ |
| **Abacavir** | McDowell et al. [107]  randomized, open-label, cross-over clinical trial | 25, HIV (+)  All males  19 Caucasian, 5 African-American, 1 Other | 600 | tablets | 0.7 | AUC ↑ 41%, C_max_ ↑ 15%, t_1/2_ ↑ 26%, t_max_ not specified | no significant changes in AUC and C_max_, t_max_ not specified |
| **Efavirenz** | McCance-Katz et al. [108]  randomized, double-blind, placebo-controlled clinical trial | 10, HIV (+)  9 males, 1 female  6 Caucasian, 4 African-American | not specified | tablets | 1 | no significant changes in AUC, C_max_ and t_max_ | AUC ↓ 14%, C_max_ ↓ 12%, t_max_ ↑ 50% |
| **Ritonavir** | McCance-Katz et al. [108]  randomized, double-blind, placebo-controlled clinical trial | 10, HIV (+)  9 males, 1 female  4 Caucasian, 2 African-American, 4 Hispanic | not specified | tablets | 1 | no significant changes in AUC, C_max_, t_max_ and t_1/2_ | AUC ↓ 15%, C_max_ ↓ 12%, t_max_ ↑ 13% |

**Supplementary material S3**

**Cochrane risk-of-bias tool for cross-over trials (RoB 2)**

| **Study ID** | **D1** | **DS** | **D2** | **D3** | **D4** | **D5** | **Overall** |  |  |
| --- | --- | --- | --- | --- | --- | --- | --- | --- | --- |
| Aarnoutse2003 |  |  |  |  |  |  |  |  | Low risk |
| Angel1993 |  |  |  |  |  |  |  |  | Some concerns |
| Behm2017-1 |  |  |  |  |  |  |  |  | High risk |
| Behm2017-2 |  |  |  |  |  |  |  |  |  |
| Brainard2011 |  |  |  |  |  |  |  | **D1** | Randomisation process |
| Brouwers2007 |  |  |  |  |  |  |  | **DS** | Bias arising from period and carryover effects |
| Carver1999 |  |  |  |  |  |  |  | **D2** | Deviations from the intended interventions |
| Chittick1999 |  |  |  |  |  |  |  | **D3** | Missing outcome data |
| Crauwels2013 |  |  |  |  |  |  |  | **D4** | Measurement of the outcome |
| Crauwels2016 |  |  |  |  |  |  |  | **D5** | Selection of the reported result |
| Crauwels2019 |  |  |  |  |  |  |  |  |  |
| Custodio2013 |  |  |  |  |  |  |  |  |  |
| Custodio2015-1 |  |  |  |  |  |  |  |  |  |
| Custodio2015-2 |  |  |  |  |  |  |  |  |  |
| Damle2002-1 |  |  |  |  |  |  |  |  |  |
| Damle2002-2 |  |  |  |  |  |  |  |  |  |
| Damle2002-3 |  |  |  |  |  |  |  |  |  |
| Demarles2002 |  |  |  |  |  |  |  |  |  |
| Dumitrescu2020 |  |  |  |  |  |  |  |  |  |
| Falcoz2002 |  |  |  |  |  |  |  |  |  |
| Gallicano2003 |  |  |  |  |  |  |  |  |  |
| Holdich2008 |  |  |  |  |  |  |  |  |  |
| Kaeser2005 |  |  |  |  |  |  |  |  |  |
| Kakuda2014 |  |  |  |  |  |  |  |  |  |
| Kakuda2014_2-1 |  |  |  |  |  |  |  |  |  |
| Kakuda2014_2-2 |  |  |  |  |  |  |  |  |  |
| Kakuda2014_3 |  |  |  |  |  |  |  |  |  |
| Kanter2010 |  |  |  |  |  |  |  |  |  |
| Kaul1998 |  |  |  |  |  |  |  |  |  |
| Kaul2010 |  |  |  |  |  |  |  |  |  |
| Kearney2005 |  |  |  |  |  |  |  |  |  |
| Kenyon1998 |  |  |  |  |  |  |  |  |  |
| Klein2007-1 |  |  |  |  |  |  |  |  |  |
| Klein2007-2 |  |  |  |  |  |  |  |  |  |
| Klein2007-3 |  |  |  |  |  |  |  |  |  |
| Knupp1993 |  |  |  |  |  |  |  |  |  |
| Krishna2018 |  |  |  |  |  |  |  |  |  |
| Kupferschmidt2020 |  |  |  |  |  |  |  |  |  |
| Kurowski2002 |  |  |  |  |  |  |  |  |  |
| Lamorde2012 |  |  |  |  |  |  |  |  |  |
| Lamorde2012_2 |  |  |  |  |  |  |  |  |  |
| Li2021 |  |  |  |  |  |  |  |  |  |
| Li2021_2 |  |  |  |  |  |  |  |  |  |
| Lotterer1991 |  |  |  |  |  |  |  |  |  |
| Lu2012 |  |  |  |  |  |  |  |  |  |
| Majeed2020 |  |  |  |  |  |  |  |  |  |
| Marier2006 |  |  |  |  |  |  |  |  |  |
| McDowell2000 |  |  |  |  |  |  |  |  |  |
| Mehta2020 |  |  |  |  |  |  |  |  |  |
| Moore1999 |  |  |  |  |  |  |  |  |  |
| Morse2003 |  |  |  |  |  |  |  |  |  |
| Nazareno1995 |  |  |  |  |  |  |  |  |  |
| Ng2008 |  |  |  |  |  |  |  |  |  |
| Oki2004 |  |  |  |  |  |  |  |  |  |
| Patel2018 |  |  |  |  |  |  |  |  |  |
| Patel2019 |  |  |  |  |  |  |  |  |  |
| Penzak2002 |  |  |  |  |  |  |  |  |  |
| Rhee2014 |  |  |  |  |  |  |  |  |  |
| Sadler1999 |  |  |  |  |  |  |  |  |  |
| Sahai1992 |  |  |  |  |  |  |  |  |  |
| Salem2015-1 |  |  |  |  |  |  |  |  |  |
| Salem2015-2 |  |  |  |  |  |  |  |  |  |
| Scholler2008 |  |  |  |  |  |  |  |  |  |
| Sekar2007 |  |  |  |  |  |  |  |  |  |
| Sevinsky2014 |  |  |  |  |  |  |  |  |  |
| Shelton1994 |  |  |  |  |  |  |  |  |  |
| Shelton2001 |  |  |  |  |  |  |  |  |  |
| Shelton2003 |  |  |  |  |  |  |  |  |  |
| Shiomi2014 |  |  |  |  |  |  |  |  |  |
| Shyu1991 |  |  |  |  |  |  |  |  |  |
| Song2011 |  |  |  |  |  |  |  |  |  |
| Song2015 |  |  |  |  |  |  |  |  |  |
| Unadkat1990 |  |  |  |  |  |  |  |  |  |
| Veldkamp2001 |  |  |  |  |  |  |  |  |  |
| Weller2014 |  |  |  |  |  |  |  |  |  |
| Wenning2007 |  |  |  |  |  |  |  |  |  |
| Yamada2018 |  |  |  |  |  |  |  |  |  |
| Yee2020-1 |  |  |  |  |  |  |  |  |  |
| Yee2020-2 |  |  |  |  |  |  |  |  |  |
| Yeh1998-1 |  |  |  |  |  |  |  |  |  |
| Yonemura2018 |  |  |  |  |  |  |  |  |  |
| Yuen2001 |  |  |  |  |  |  |  |  |  |

| **Study ID** | **D1** | **D2** | **D3** | **D4** | **D5** | **Overall** |  |  |  |
| --- | --- | --- | --- | --- | --- | --- | --- | --- | --- |
| Aarnoutse2003_2 |  |  |  |  |  |  |  |  | Low risk |
| Anderson2014 |  |  |  |  |  |  |  |  | Some concerns |
| Fatkenheuer2005 |  |  |  |  |  |  |  |  | High risk |
| Gruber2013 |  |  |  |  |  |  |  |  |  |
| Han2014 |  |  |  |  |  |  |  | **D1** | Randomisation process |
| Hernandez2008 |  |  |  |  |  |  |  | **D2** | Deviations from the intended interventions |
| Li2021_3 |  |  |  |  |  |  |  | **D3** | Missing outcome data |
| Mathias2018 |  |  |  |  |  |  |  | **D4** | Measurement of the outcome |
| Mccance-Katz2013 |  |  |  |  |  |  |  | **D5** | Selection of the reported result |
| Patel2018_2 |  |  |  |  |  |  |  |  |  |
| Ruhnke1993 |  |  |  |  |  |  |  |  |  |
| Saah2001 |  |  |  |  |  |  |  |  |  |
| Yeh1998-2 |  |  |  |  |  |  |  |  |  |

**Cochrane risk-of-bias tool for parallel trials (RoB 2)**

**NIH Quality Assessment Tool for Observational Cohort Studies**

| **Criteria** | **Lopez2006** | | | **Sanchez2007** | | |
| --- | --- | --- | --- | --- | --- | --- |
|  | **Yes** | **No** | **Other***  **(CD, NA, NR)** | **Yes** | **No** | **Other***  **(CD, NA, NR)** |
| 1. Was the research question or objective in this paper clearly stated? | X |  |  | X |  |  |
| 2. Was the study population clearly specified and defined? | X |  |  | X |  |  |
| 3. Was the participation rate of eligible persons at least 50%? | X |  |  | X |  |  |
| 4. Were all the subjects selected or recruited from the same or similar populations (including the same time period)? Were inclusion and exclusion criteria for being in the study prespecified and applied uniformly to all participants? | X |  |  | X |  |  |
| 5. Was a sample size justification, power description, or variance and effect estimates provided? |  | X |  |  | X |  |
| 6. For the analyses in this paper, were the exposure(s) of interest measured prior to the outcome(s) being measured? | X |  |  | X |  |  |
| 7. Was the timeframe sufficient so that one could reasonably expect to see an association between exposure and outcome if it existed? | X |  |  | X |  |  |
| 8. For exposures that can vary in amount or level, did the study examine different levels of the exposure as related to the outcome (e.g., categories of exposure, or exposure measured as continuous variable)? |  |  | NA |  |  | NA |
| 9. Were the exposure measures (independent variables) clearly defined, valid, reliable, and implemented consistently across all study participants? | X |  |  | X |  |  |
| 10. Was the exposure(s) assessed more than once over time? |  |  | NA | X |  |  |
| 11. Were the outcome measures (dependent variables) clearly defined, valid, reliable, and implemented consistently across all study participants? | X |  |  | X |  |  |
| 12. Were the outcome assessors blinded to the exposure status of participants? |  |  | NA |  | X |  |
| 13. Was loss to follow-up after baseline 20% or less? |  | X |  | X |  |  |
| 14. Were key potential confounding variables measured and adjusted statistically for their impact on the relationship between exposure(s) and outcome(s)? | X |  |  |  | X |  |
| **Overall quality rating** | **fair** | | | **poor** | | |

* CD – cannot determine, NA – not applicable, NR – not reported

**NIH Quality Assessment Tool for Before-After (Pre-Post) Studies With No Separate Control Group**

| **Criteria** | **Hugen2002** | | | **Lamorde2015** | | | **Stevens2000** | | | **Piscitelli2002** | | |
| --- | --- | --- | --- | --- | --- | --- | --- | --- | --- | --- | --- | --- |
|  | **Yes** | **No** | **Other**  **(CD, NA, NR)** | **Yes** | **No** | **Other**  **(CD, NA, NR)** | **Yes** | **No** | **Other**  **(CD, NA, NR)** | **Yes** | **No** | **Other**  **(CD, NA, NR)** |
| 1. Was the study question or objective clearly stated? | X |  |  | X |  |  | X |  |  | X |  |  |
| 2. Were eligibility/selection criteria for the study population prespecified and clearly described? |  | X |  | X |  |  | X |  |  | X |  |  |
| 3. Were the participants in the study representative of those who would be eligible for the test/service/intervention in the general or clinical population of interest? |  |  | CD |  |  | CD | X |  |  |  | X |  |
| 4. Were all eligible participants that met the prespecified entry criteria enrolled? |  |  | NR |  |  | NR |  |  | NR |  |  | NR |
| 5. Was the sample size sufficiently large to provide confidence in the findings? |  | X |  | X |  |  | X |  |  |  |  | CD |
| 6. Was the test/service/intervention clearly described and delivered consistently across the study population? | X |  |  | X |  |  | X |  |  | X |  |  |
| 7. Were the outcome measures prespecified, clearly defined, valid, reliable, and assessed consistently across all study participants? | X |  |  | X |  |  | X |  |  | X |  |  |
| 8. Were the people assessing the outcomes blinded to the participants' exposures/interventions? |  |  | NR |  | X |  |  |  | NR |  |  | NR |
| 9. Was the loss to follow-up after baseline 20% or less? Were those lost to follow-up accounted for in the analysis? |  |  | NR | X |  |  |  | X |  | X |  |  |
| 10. Did the statistical methods examine changes in outcome measures from before to after the intervention? Were statistical tests done that provided p values for the pre-to-post changes? | X |  |  | X |  |  | X |  |  | X |  |  |
| 11. Were outcome measures of interest taken multiple times before the intervention and multiple times after the intervention (i.e., did they use an interrupted time-series design)? | X |  |  | X |  |  | X |  |  | X |  |  |
| 12. If the intervention was conducted at a group level (e.g., a whole hospital, a community, etc.) did the statistical analysis take into account the use of individual-level data to determine effects at the group level? |  |  | NA |  |  | NA |  |  | NA |  |  | NA |
| **Overall quality rating** | poor | | | fair | | | fair | | | fair | | |

**Supplementary material S4**

**List of studies excluded from meta-analyses**

| **StudyID** | **Reason for excluding** |
| --- | --- |
| Aarnoutse2003 | The study was excluded from a meta-analysis of AUC_inf_, because only AUC_0-12h_ was reported. |
| Aarnoutse2003_2 | The study was parallel, whereas all the remaining studies in a group were cross-over. |
| Anderson2014 | The study was parallel, whereas all the remaining studies in a group were cross-over. |
| Brainard2011 | The study was excluded from a meta-analysis of AUC_inf_, because only AUC_0-12h_ was reported. |
| Brouwers2007 | The study was non-randomized, whereas all the remaining studies in a group were randomized. |
| Chittick1999 | Values of pharmacokinetic parameters were reported as geometric least squares mean. |
| Cloarec2017 | We did not include case studies in the meta-analyses. |
| Crauwels2016 | Only the geometric least square means ratios of pharmacokinetic parameters were reported. |
| Demarles2002 | The study assessed the interaction with grapefruit juice and we included only food-effect studies in the meta-analyses. |
| Gruber2003 | The study assessed the interaction with alcohol and we included only food-effect studies in the meta-analyses. |
| Han2014 | The study was parallel, whereas all the remaining studies in a group were cross-over. |
| Hernandez2008 | No values of pharmacokinetic parameters were reported. |
| Hugen2002 | The study was longitudinal and non-randomized, whereas all the remaining studies in a group were cross-over and randomized. Additionally, a standard meal was used as a control (and not a fasting state). |
| Jiang2013 | No values of pharmacokinetic parameters were reported. |
| Kaeser2005 | Only the ratios of pharmacokinetic parameters were reported. |
| Kaul2010 | Values of pharmacokinetic parameters were reported without 95% confidence intervals. |
| Klein2007-1 | Only the geometric least square means ratios of pharmacokinetic parameters were reported. |
| Klein2007-2 | Only the geometric least square means ratios of pharmacokinetic parameters were reported. |
| Klein2007-3 | Only the geometric least square means ratios of pharmacokinetic parameters were reported. |
| Kupferschmidt1998 | The study assessed the interaction with grapefruit juice and we included only food-effect studies in the meta-analyses. |
| Kurowski2002 | Only percentage changes in pharmacokinetic parameters were reported. |
| Lamorde2015 | The study was longitudinal and non-randomized, whereas all the remaining studies in a group were cross-over and randomized. |
| Lamorde2012 | The study was non-randomized, whereas all the remaining studies in a group were randomized. |
| Lamorde2012_2 | The study was non-randomized, whereas all the remaining studies in a group were randomized. |
| Li2021_3 | The study was parallel, whereas all the remaining studies in a group were cross-over. |
| Lopez2006 | We did not include cohort studies in the meta-analyses. |
| Majeed2020 | Values of pharmacokinetic parameters were reported without 95% confidence intervals. |
| Marier2006 | Values of pharmacokinetic parameters were reported without standard deviation. |
| Mathias2018 | The study assessed the interaction with dietary supplements and we included only food-effect studies in the meta-analyses. |
| McCance-Katz2013 | The study assessed the interaction with alcohol and we included only food-effect studies in the meta-analyses. |
| McDowell2000 | The study assessed the interaction with alcohol and we included only food-effect studies in the meta-analyses. |
| Patel2018 | The study was parallel, whereas all the remaining studies in a group were cross-over. |
| Patel2018_2 | No values of pharmacokinetic parameters were reported. |
| Piscitelli2002 | The study assessed the interaction with garlic supplements and we included only food-effect studies in the meta-analyses. |
| Rhee2014 | Values of pharmacokinetic parameters were reported without 95% confidence intervals. |
| Ruhnke1993 | The study was parallel, whereas all the remaining studies in a group were cross-over. |
| Saah2001 | The low-fat meal was used as a control (and not a fasting state). |
| Salem2008-1 | Only the geometric least square means ratios of pharmacokinetic parameters were reported. |
| Salem2008-2 | Only the geometric least square means ratios of pharmacokinetic parameters were reported. |
| Sanchez2007 | We did not include cohort studies in the meta-analyses. |
| Sevinsky2014 | Values of pharmacokinetic parameters were reported without 95% confidence intervals. |
| Song2015 | The study assessed the interaction with dietary supplements and we included only food-effect studies in the meta-analyses. |
| Stevens2000 | The study was longitudinal, whereas all the remaining studies in a group were cross-over. |
| Veldkamp2001 | A standard meal was used as a control (and not a fasting state). |
| Weller2014 | Values of pharmacokinetic parameters were reported without 95% confidence intervals. |
| Yeh1998-1 | The study was parallel, whereas all the remaining studies in a group were cross-over. |
| Yonemura2018 | The study assessed the interaction with milk and apple juice and we included only food-effect studies in the meta-analyses. |

**Supplementary material S5**

**Forest plots of the performed meta-analyses, subgroup analyses and sensitivity analyses**

**Didanosine**


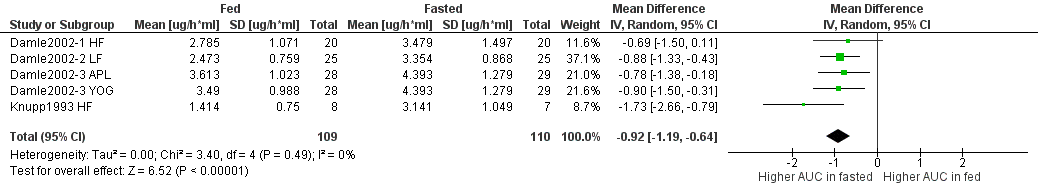


Figure S1. Forest plot showing the mean difference in AUC_inf_ of didanosine under fasted and fed conditions – only randomized studies included (HF – high-fat meal, LF – low-fat meal, APL – apple sauce, YOG – yoghurt).


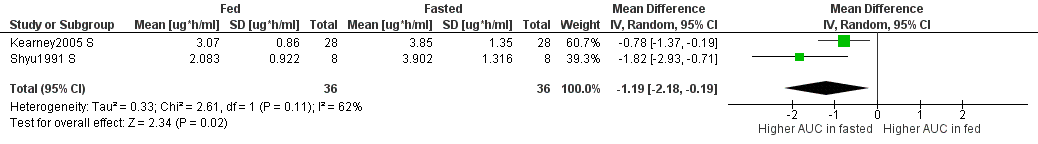


Figure S2. Forest plot showing the mean difference in AUC_inf_ of didanosine under fasted and fed conditions – only non-randomized studies included (S – standard meal).


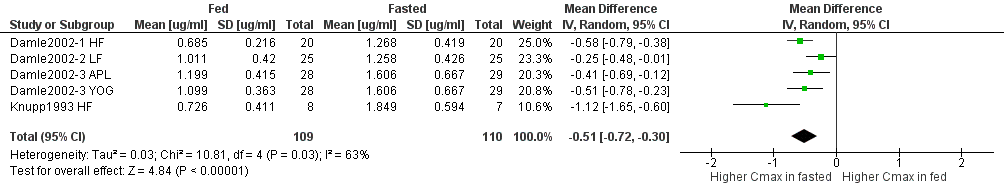


Figure S3. Forest plot showing the mean difference in C_max_ of didanosine under fasted and fed conditions – only randomized studies included (HF – high-fat meal, LF – low-fat meal, APL – apple sauce, YOG – yoghurt).


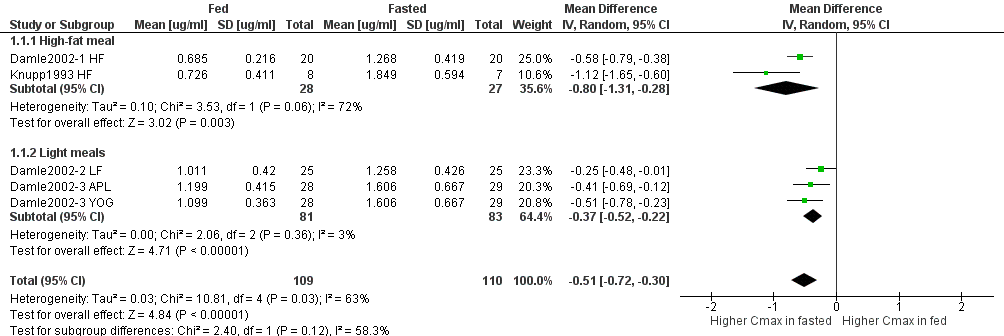


Figure. S3.1. Forest plot showing the mean difference in C_max_ of didanosine under fasted and fed conditions by type of meal – only randomized studies included (HF – high-fat meal, LF – low-fat meal, APL – apple sauce, YOG – yoghurt).


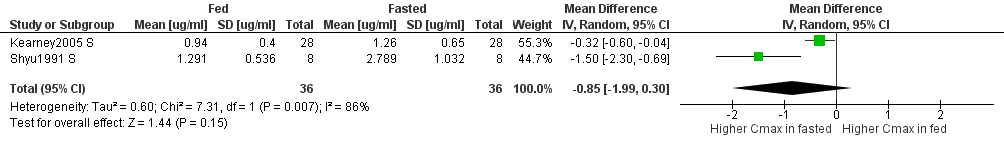


Figure S4. Forest plot showing the mean difference in C_max_ of didanosine under fasted and fed conditions – only non-randomized studies included (S – standard meal). **Random effects model.**


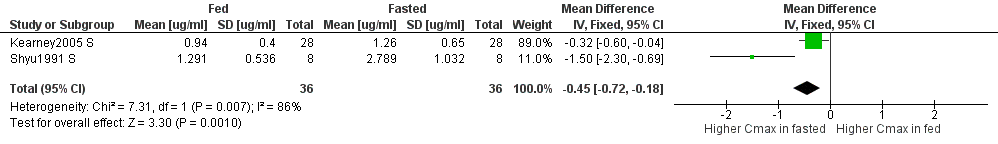


Figure S4.1. Forest plot showing the mean difference in C_max_ of didanosine under fasted and fed conditions – only non-randomized studies included (S – standard meal). **Fixed effects model.**


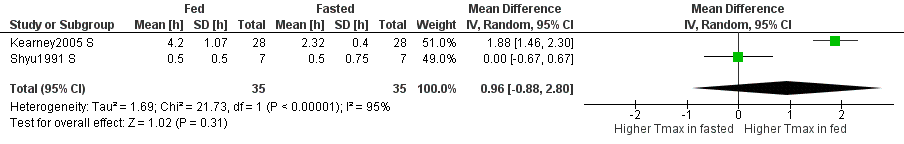


Figure S5. Forest plot showing the mean difference in T_max_ of didanosine under fasted and fed conditions – only non-randomized studies included (S – standard meal). **Random effects model.**


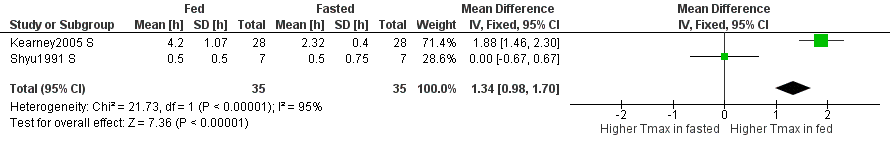


Figure S5.1. Forest plot showing the mean difference in T_max_ of didanosine under fasted and fed conditions – only non-randomized studies included (S – standard meal). **Fixed effects model.**

**Emtricitabine**


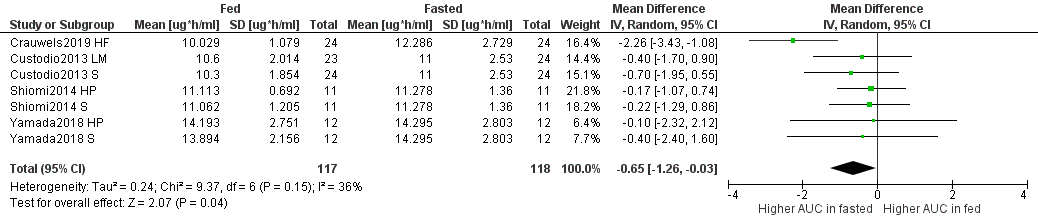


Figure S6. Forest plot showing the mean difference in AUC_inf_ of emtricitabine under fasted and fed conditions (HF – high-fat meal, LM – light meal, S – standard meal, HP – high-protein meal).


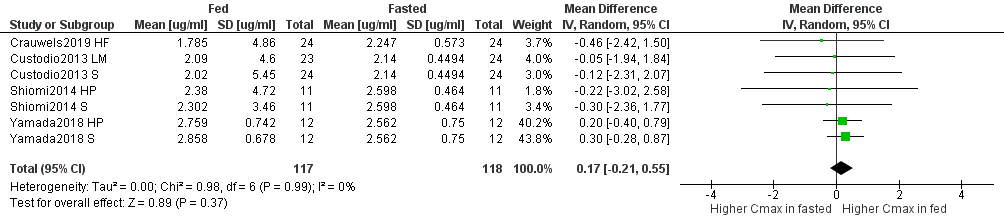


Figure S7. Forest plot showing the mean difference in C_max_ of emtricitabine under fasted and fed conditions (HF – high-fat meal, LM – light meal, S – standard meal, HP – high-protein meal).


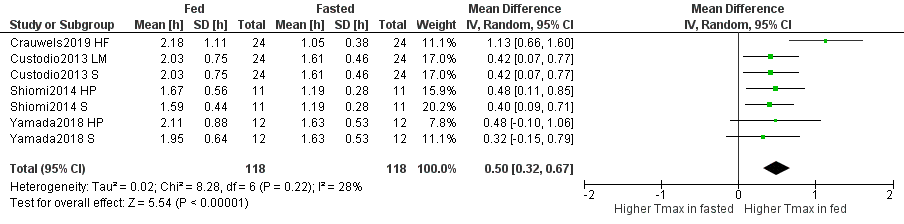


Figure S8. Forest plot showing the mean difference in T_max_ of emtricitabine under fasted and fed conditions (HF – high-fat meal, LM – light meal, S – standard meal, HP – high-protein meal).

**Lamivudine**


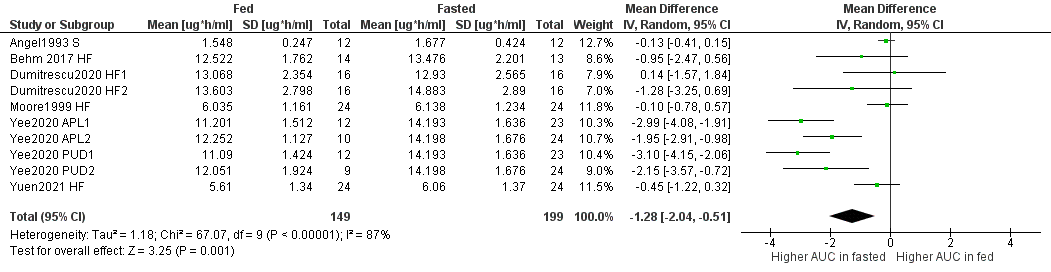


Figure S9. Forest plot showing the mean difference in AUC_inf_ of lamivudine under fasted and fed conditions (S – standard meal, HF – high-fat meal, APL – apple sauce, PUD - pudding).


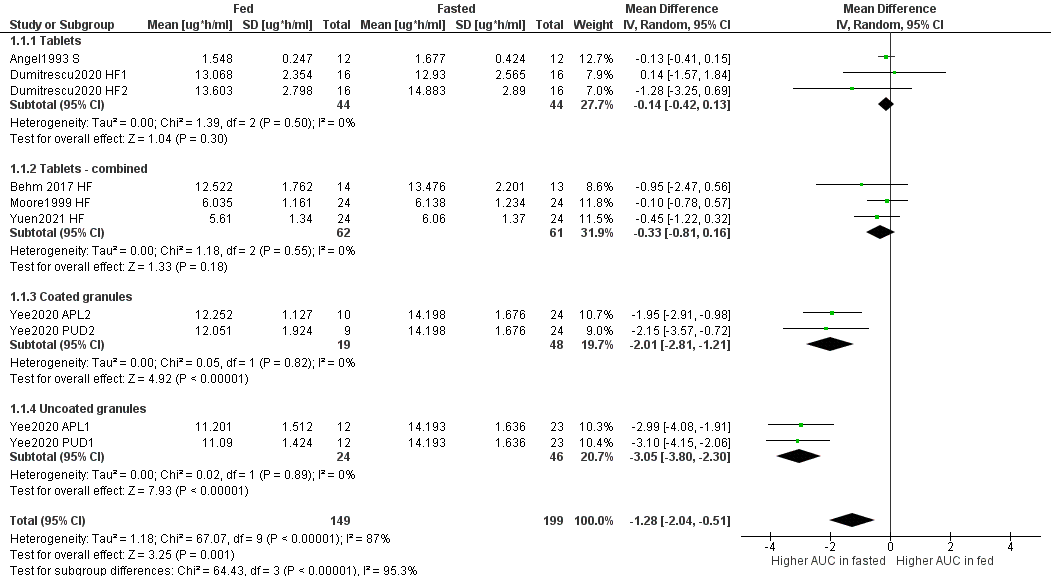


Figure S9.1. Forest plot showing the mean difference in AUC_inf_ of lamivudine under fasted and fed conditions by drug formulation (S – standard meal, HF – high-fat meal, APL – apple sauce, PUD - pudding).


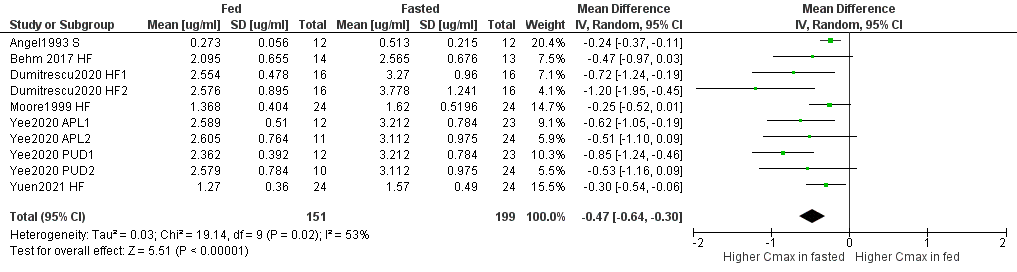


Figure S10. Forest plot showing the mean difference in C_max_ of lamivudine under fasted and fed conditions (S – standard meal, HF – high-fat meal, APL – apple sauce, PUD - pudding).


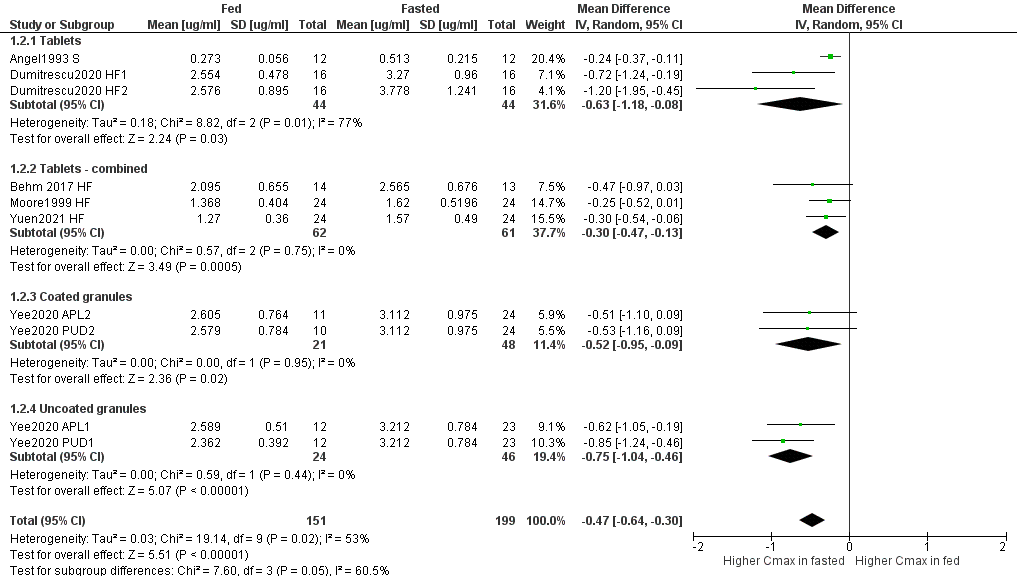


Figure S10.1. Forest plot showing the mean difference in C_max_ of lamivudine under fasted and fed conditions by drug formulation (S – standard meal, HF – high-fat meal, APL – apple sauce, PUD - pudding).


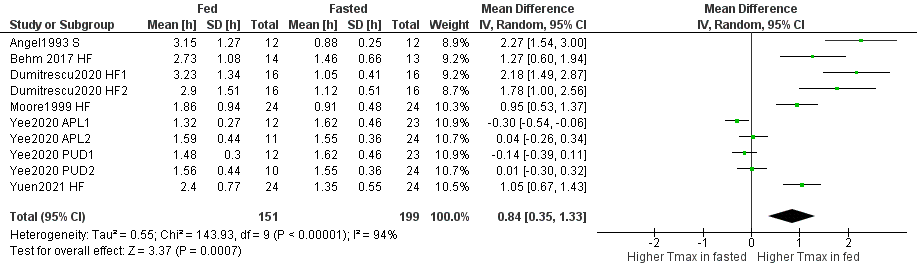


Figure S11. Forest plot showing the mean difference in T_max_ of lamivudine under fasted and fed conditions (S – standard meal, HF – high-fat meal, APL – apple sauce, PUD - pudding).


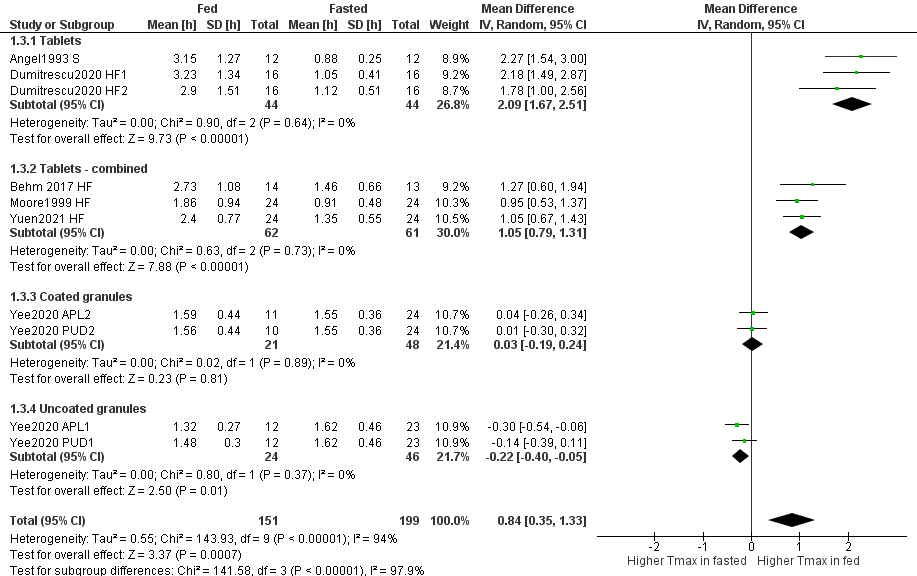


Figure S11.1. Forest plot showing the mean difference in T_max_ of lamivudine under fasted and fed conditions by drug formulation (S – standard meal, HF – high-fat meal, APL – apple sauce, PUD - pudding).

**Tenofovir disoproxil**


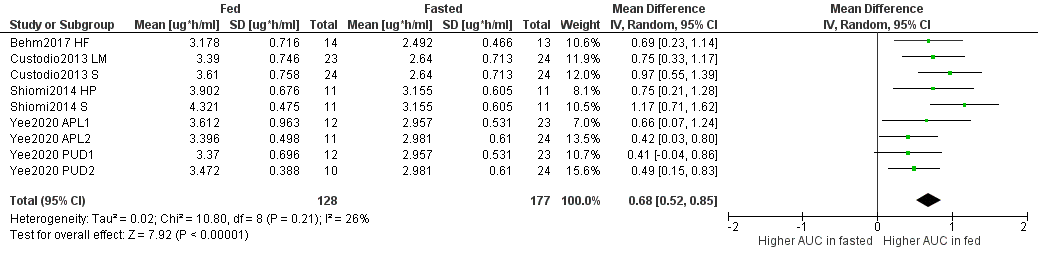


Figure S12. Forest plot showing the mean difference in AUC_inf_ of tenofovir disoproxil under fasted and fed conditions (HF – high-fat meal, LM – light meal, S – standard meal, HP – high-protein meal, APL – apple sauce, PUD - pudding).


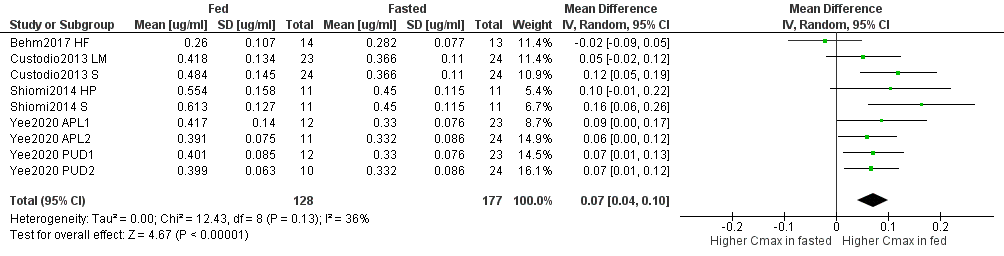


Figure S13. Forest plot showing the mean difference in C_max_ of tenofovir disoproxil under fasted and fed conditions (HF – high-fat meal, LM – light meal, S – standard meal, HP – high-protein meal, APL – apple sauce, PUD - pudding).


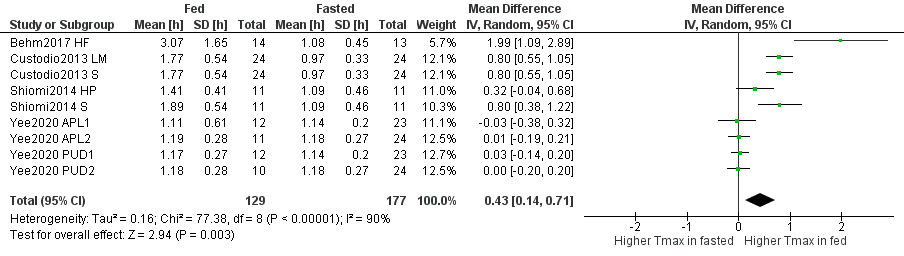


Figure S14. Forest plot showing the mean difference in T_max_ of tenofovir disoproxil under fasted and fed conditions (HF – high-fat meal, LM – light meal, S – standard meal, HP – high-protein meal, APL – apple sauce, PUD - pudding).


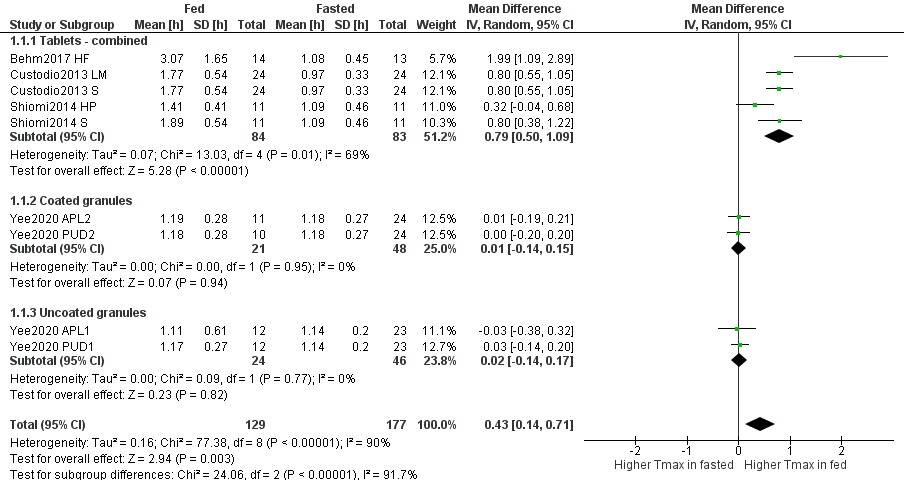


Figure S14. Forest plot showing the mean difference in T_max_ of tenofovir disoproxil under fasted and fed conditions by drug formulation (HF – high-fat meal, LM – light meal, S – standard meal, HP – high-protein meal, APL – apple sauce, PUD - pudding).

**Tenofovir alafenamide**


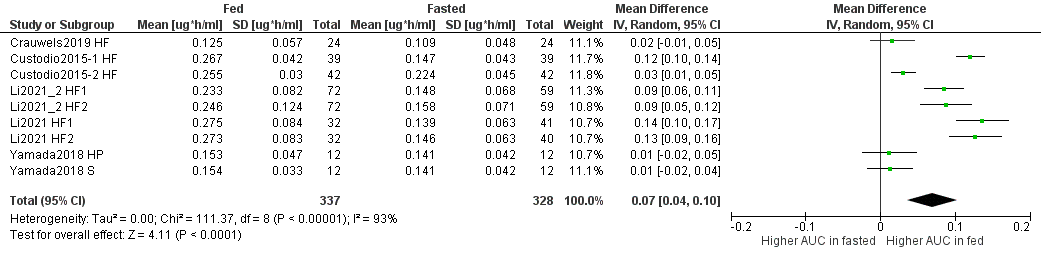


Figure S15. Forest plot showing the mean difference in AUC_inf_ of tenofovir alafenamide under fasted and fed conditions (HF – high-fat meal, HP – high-protein meal, S – standard meal).


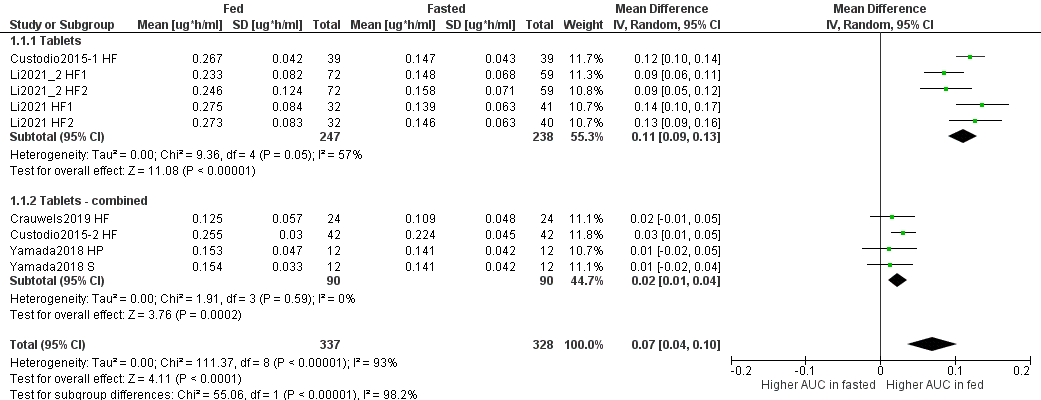


Figure S15.1. Forest plot showing the mean difference in AUC_inf_ of tenofovir alafenamide under fasted and fed conditions by drug formulation (HF – high-fat meal, HP – high-protein meal, S – standard meal).


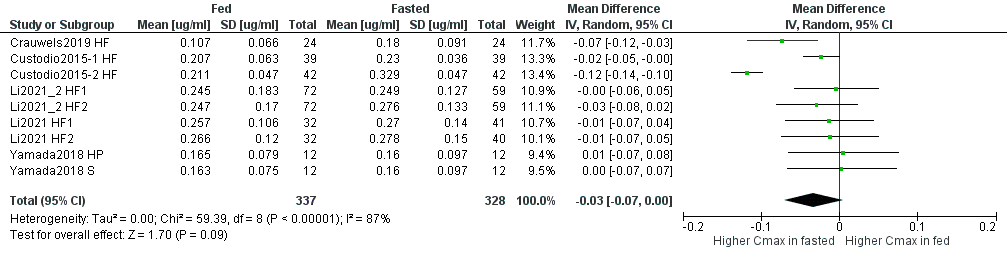


Figure S16. Forest plot showing the mean difference in C_max_ of tenofovir alafenamide under fasted and fed conditions (HF – high-fat meal, HP – high-protein meal, S – standard meal). **Random effects model.**


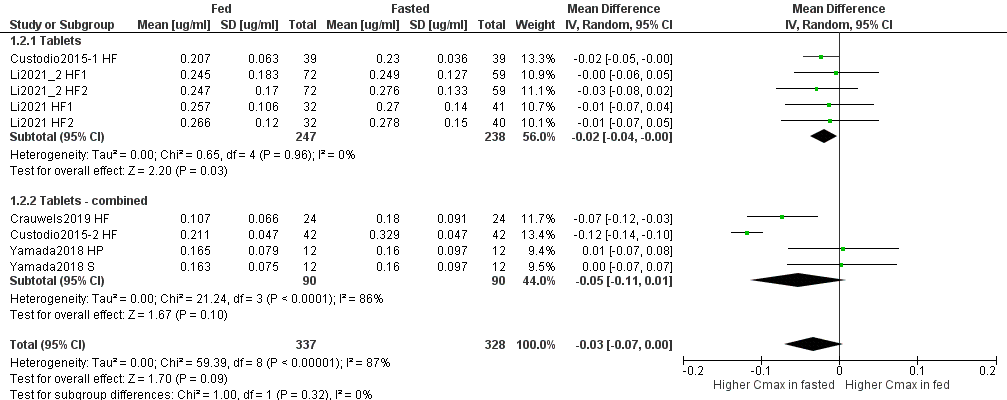


Figure S16.1. Forest plot showing the mean difference in C_max_ of tenofovir alafenamide under fasted and fed conditions by drug formulation (HF – high-fat meal, HP – high-protein meal, S – standard meal).


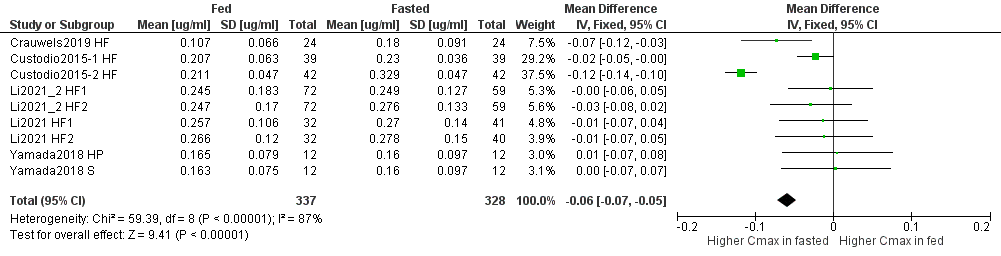


Figure S16.2. Forest plot showing the mean difference in C_max_ of tenofovir alafenamide under fasted and fed conditions (HF – high-fat meal, HP – high-protein meal, S – standard meal). **Fixed effects model.**


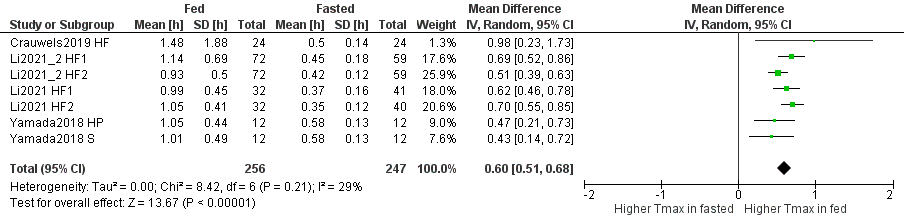


Figure S17. Forest plot showing the mean difference in T_max_ of tenofovir alafenamide under fasted and fed conditions (HF – high-fat meal, HP – high-protein meal, S – standard meal).

**Zidovudine**


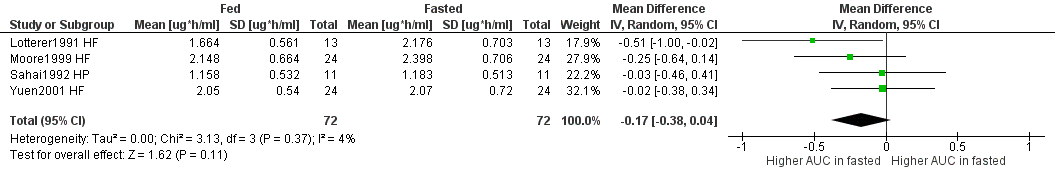


Figure S18. Forest plot showing the mean difference in AUC_inf_ of zidovudine under fasted and fed conditions (HF – high-fat meal, HP – high-protein meal).


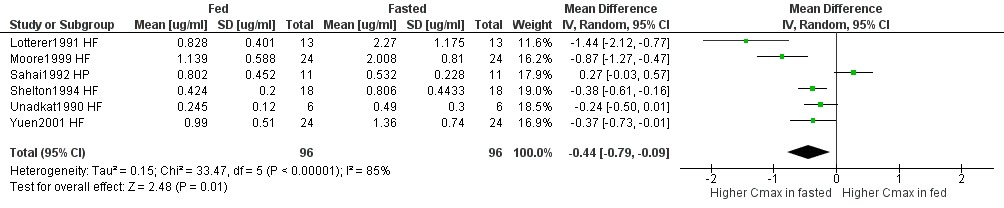


Figure S19. Forest plot showing the mean difference in C_max_ of zidovudine under fasted and fed conditions (HF – high-fat meal, HP – high-protein meal).


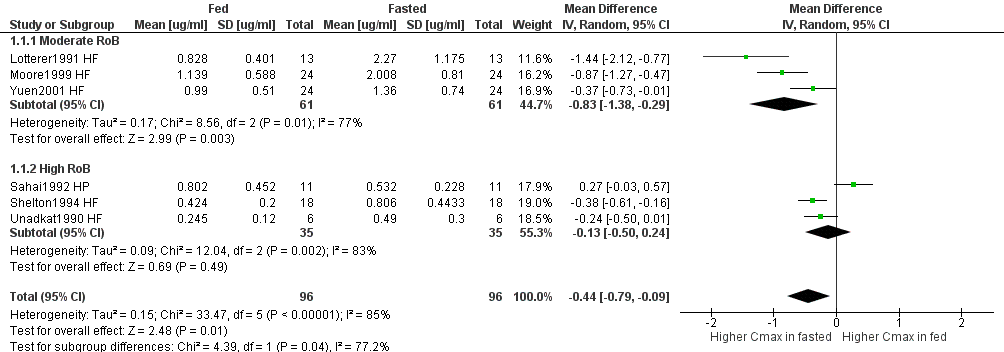


Figure S19.1. Forest plot showing the mean difference in C_max_ of zidovudine under fasted and fed conditions by study risk of bias (HF – high-fat meal, HP – high-protein meal).


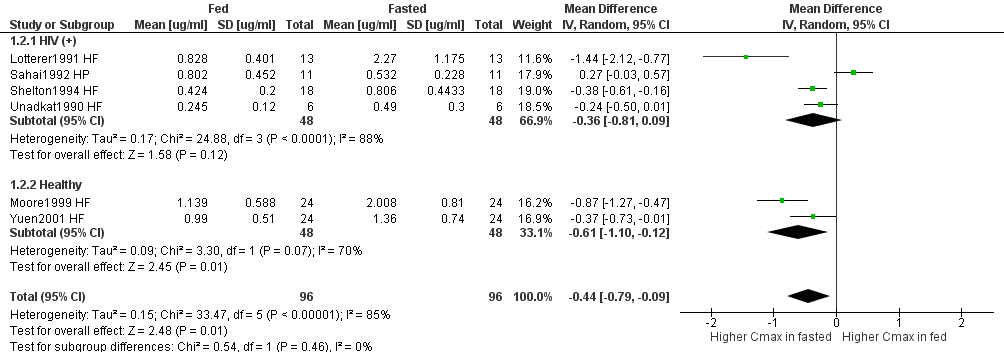


Figure S19.2. Forest plot showing the mean difference in C_max_ of zidovudine under fasted and fed conditions by participants health state (HF – high-fat meal, HP – high-protein meal).


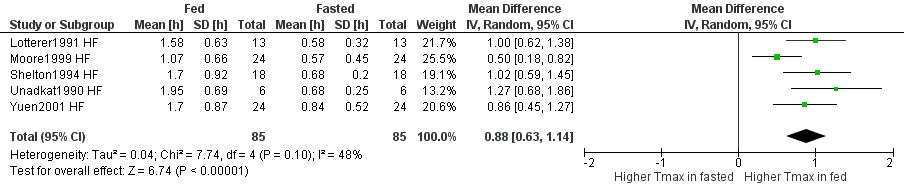


Figure S20. Forest plot showing the mean difference in T_max_ of zidovudine under fasted and fed conditions (HF – high-fat meal).


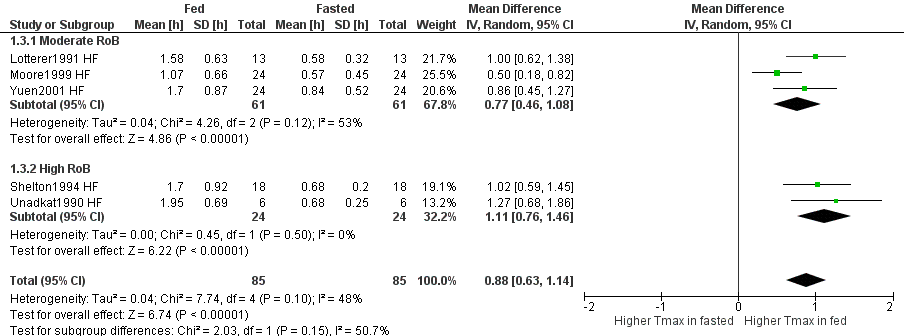


Figure S20.1. Forest plot showing the mean difference in T_max_ of zidovudine under fasted and fed conditions by study risk of bias (HF – high-fat meal).


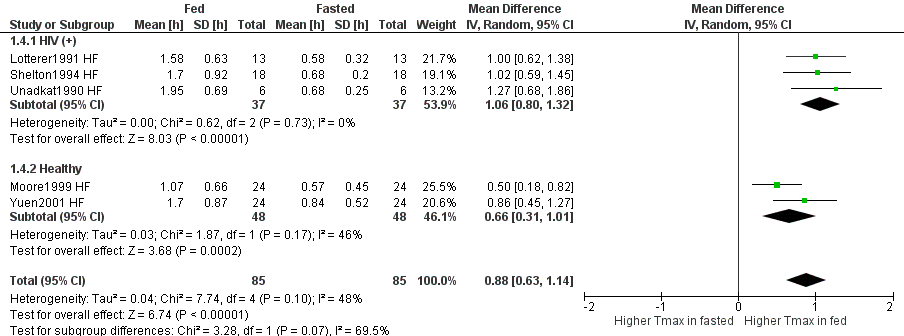


Figure S20.2. Forest plot showing the mean difference in T_max_ of zidovudine under fasted and fed conditions by participants health state (HF – high-fat meal).

**Doravirine**


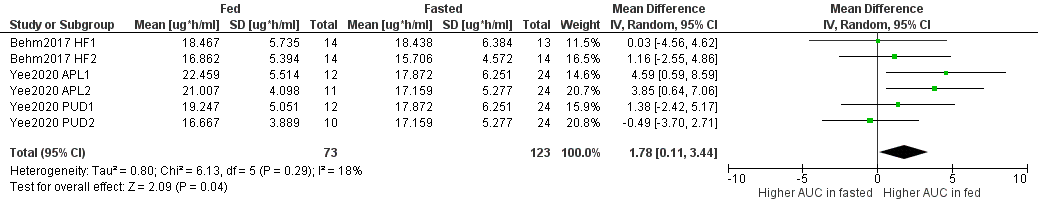


Figure S21. Forest plot showing the mean difference in AUC_inf_ of doravirine under fasted and fed conditions (HF – high-fat meal, APL – apple sauce, PUD - pudding).


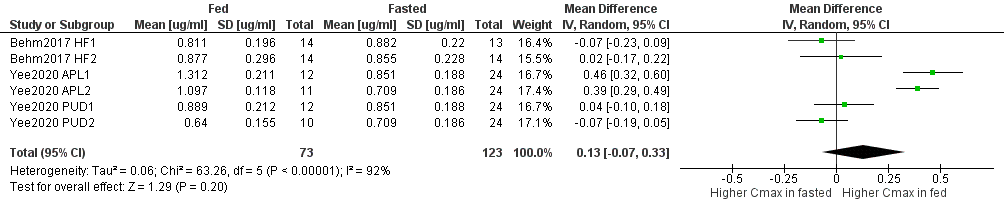


Figure S22. Forest plot showing the mean difference in C_max_ of doravirine under fasted and fed conditions (HF – high-fat meal, APL – apple sauce, PUD - pudding). **Random effects model.**


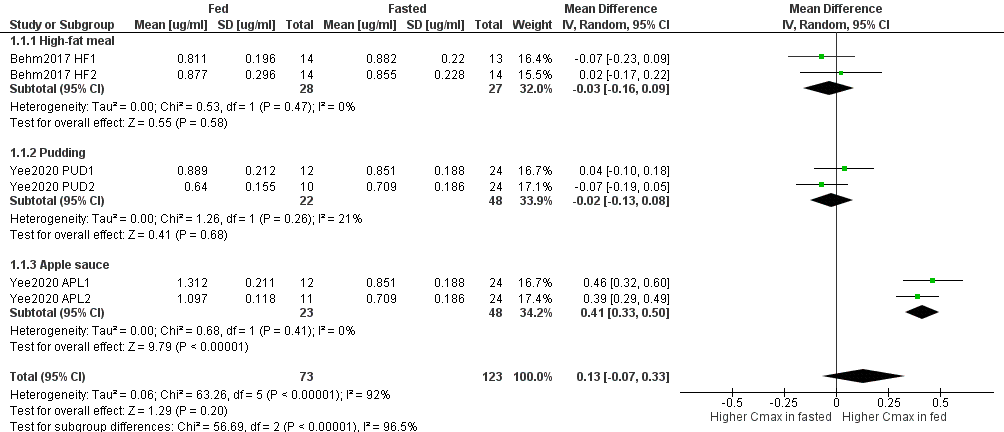


Figure S22.1. Forest plot showing the mean difference in C_max_ of doravirine under fasted and fed conditions by type of meal (HF – high-fat meal, APL – apple sauce, PUD - pudding).


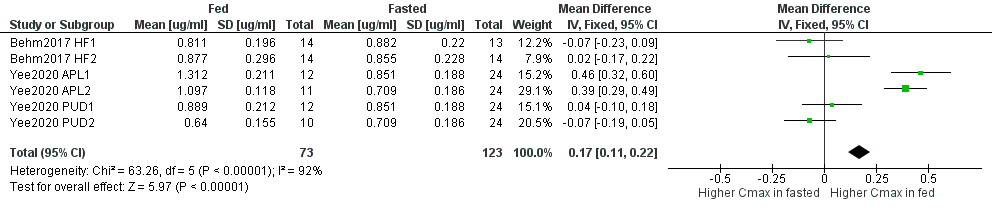


Figure S22.2 Forest plot showing the mean difference in C_max_ of doravirine under fasted and fed conditions (HF – high-fat meal, APL – apple sauce, PUD - pudding). **Fixed effects model.**


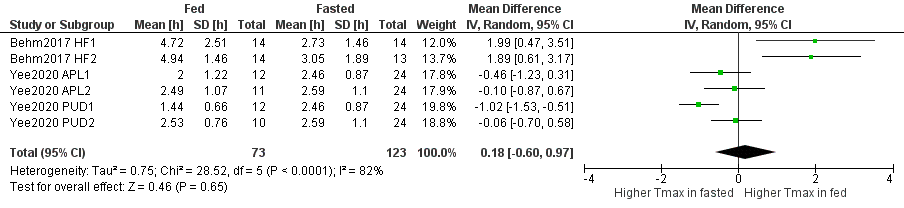


Figure S23. Forest plot showing the mean difference in T_max_ of doravirine under fasted and fed conditions (HF – high-fat meal, APL – apple sauce, PUD - pudding).


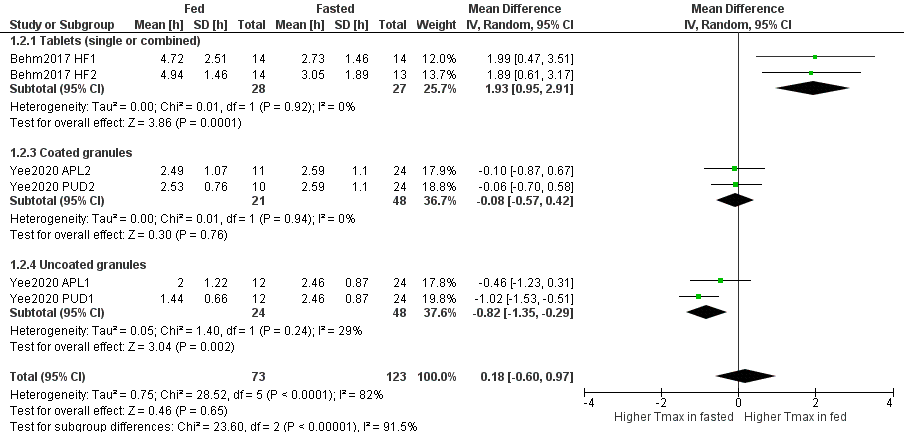


Figure S23.1. Forest plot showing the mean difference in T_max_ of doravirine under fasted and fed conditions by drug formulation (HF – high-fat meal, APL – apple sauce, PUD - pudding).


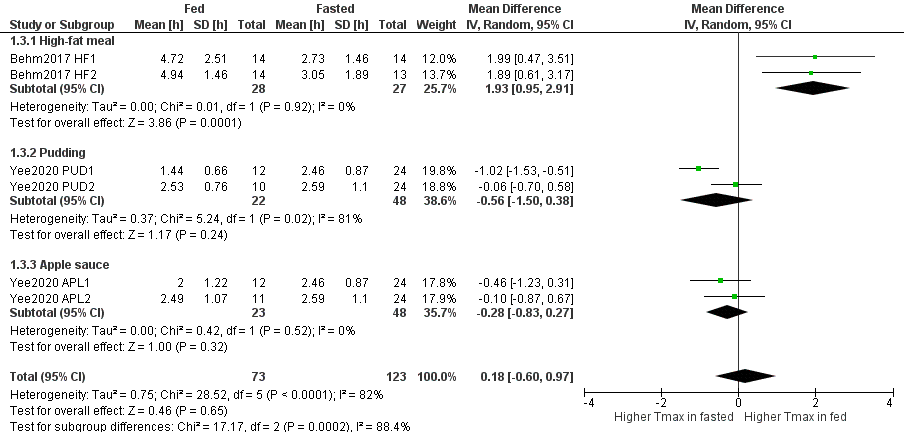


Figure S23.2. Forest plot showing the mean difference in T_max_ of doravirine under fasted and fed conditions by drug formulation (HF – high-fat meal, APL – apple sauce, PUD - pudding).

**Rilpivirine**


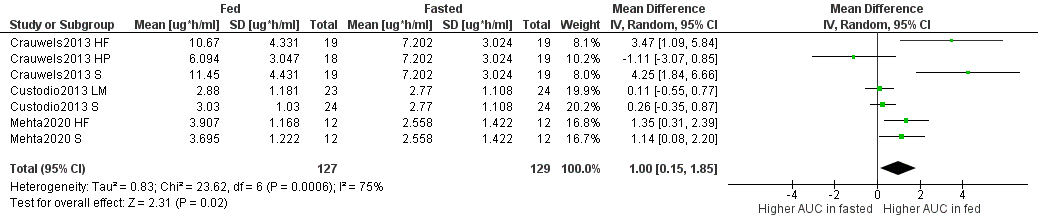


Figure S24. Forest plot showing the mean difference in AUC_inf_ of rilpivirine under fasted and fed conditions (HF – high-fat meal, HP – high-protein meal, S – standard meal, LM – light meal).


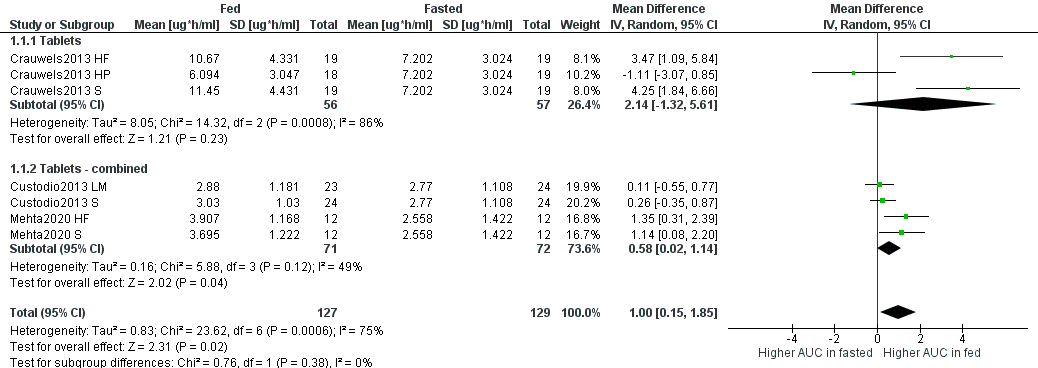


Figure S24.1. Forest plot showing the mean difference in AUC_inf_ of rilpivirine under fasted and fed conditions by drug formulation (HF – high-fat meal, HP – high-protein meal, S – standard meal, LM – light meal).


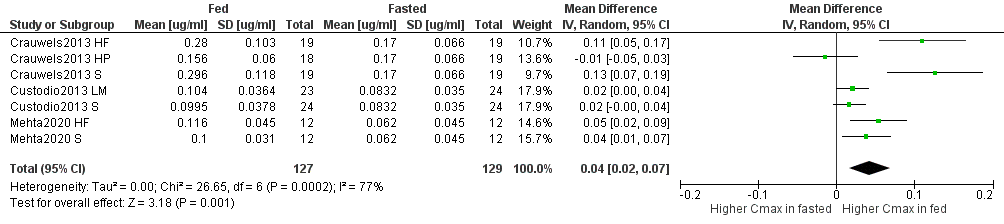


Figure S25. Forest plot showing the mean difference in C_max_ of rilpivirine under fasted and fed conditions (HF – high-fat meal, HP – high-protein meal, S – standard meal, LM – light meal).


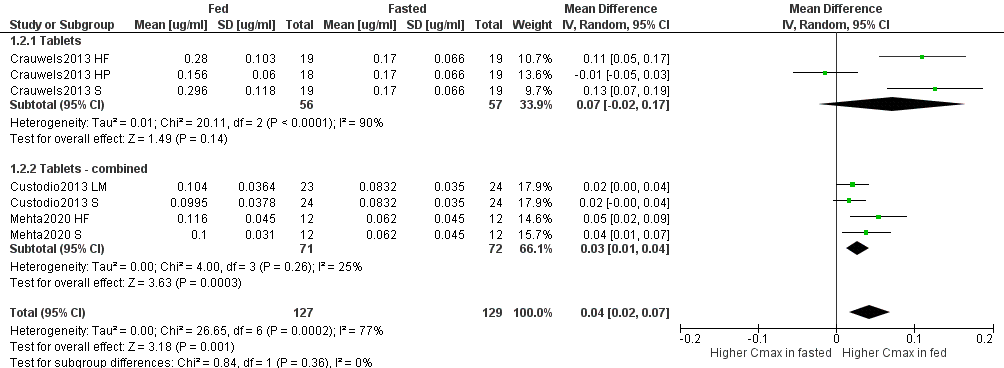


Figure S25.1. Forest plot showing the mean difference in C_max_ of rilpivirine under fasted and fed conditions by drug formulation (HF – high-fat meal, HP – high-protein meal, S – standard meal, LM – light meal).


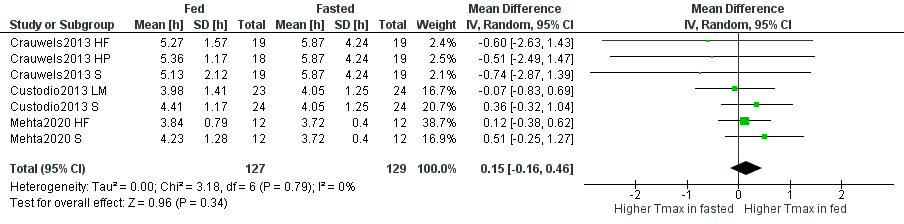


Figure S26. Forest plot showing the mean difference in T_max_ of rilpivirine under fasted and fed conditions (HF – high-fat meal, HP – high-protein meal, S – standard meal, LM – light meal).

**Dolutegravir**


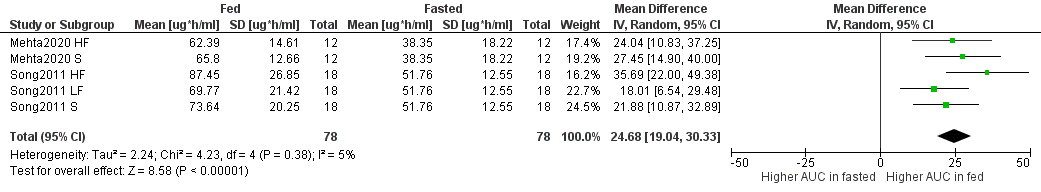


Figure S27. Forest plot showing the mean difference in AUC_inf_ of dolutegravir under fasted and fed conditions (HF – high-fat meal, S – standard meal, LF – low-fat meal).


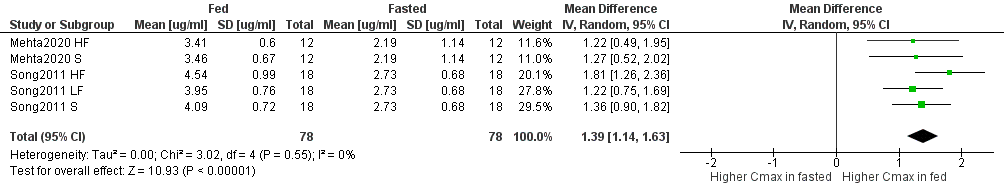


Figure S28. Forest plot showing the mean difference in C_max_ of dolutegravir under fasted and fed conditions (HF – high-fat meal, S – standard meal, LF – low-fat meal).


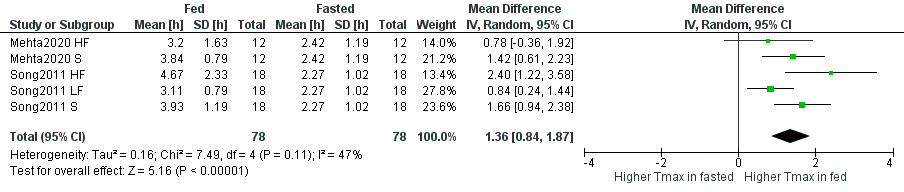


Figure S29. Forest plot showing the mean difference in T_max_ of dolutegravir under fasted and fed conditions (HF – high-fat meal, S – standard meal, LF – low-fat meal).

**Elvitegravir**


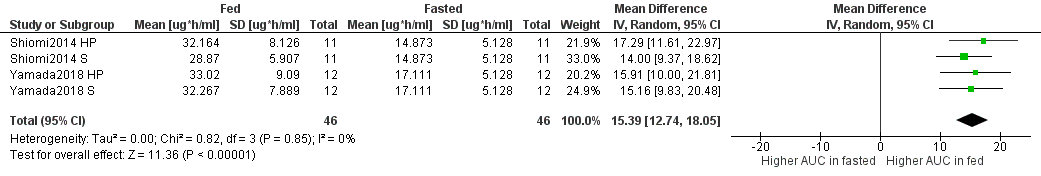


Figure S30. Forest plot showing the mean difference in AUC_inf_ of elvitegravir under fasted and fed conditions (HP – high-protein meal, S – standard meal).


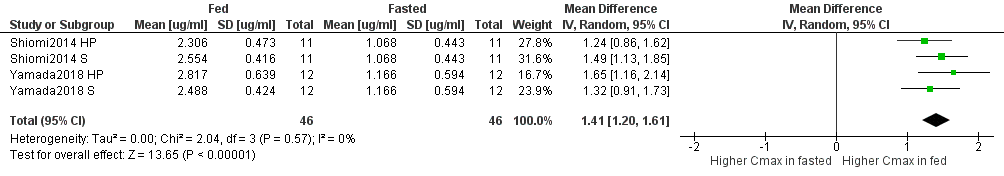


Figure S31. Forest plot showing the mean difference in C_max_ of elvitegravir under fasted and fed conditions (HP – high-protein meal, S – standard meal).


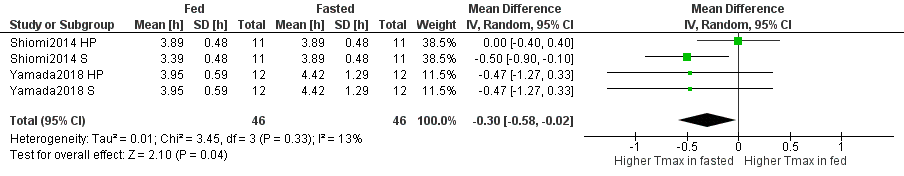


Figure S32. Forest plot showing the mean difference in T_max_ of elvitegravir under fasted and fed conditions (HP – high-protein meal, S – standard meal).

**Raltegravir**


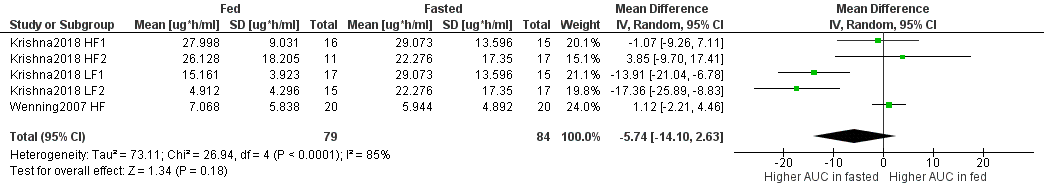


Figure S33. Forest plot showing the mean difference in AUC_inf_ of raltegravir under fasted and fed conditions (HF – high-fat meal, LF – low-fat meal). **Random effects model.**


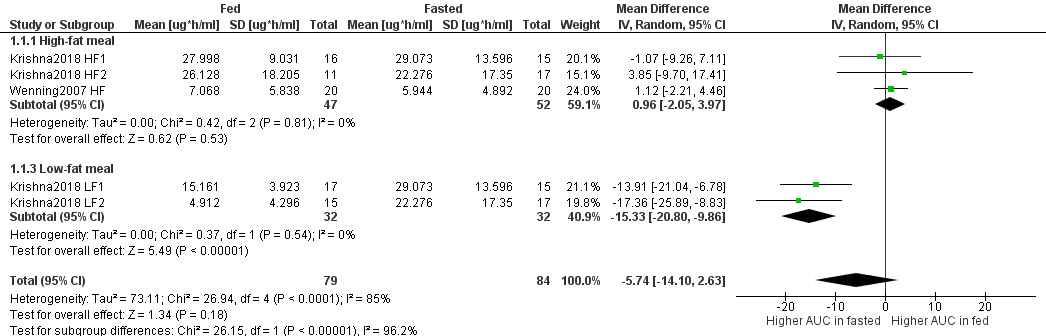


Figure S33.1. Forest plot showing the mean difference in AUC_inf_ of raltegravir under fasted and fed conditions by type of meal (HF – high-fat meal, LF – low-fat meal).


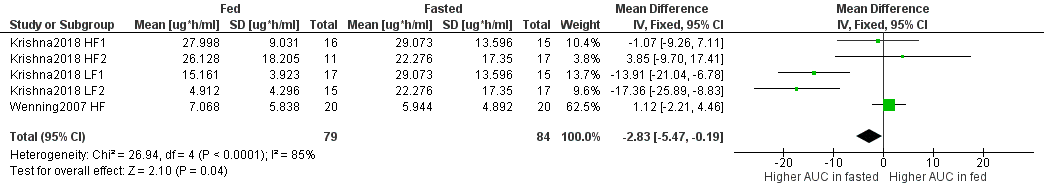


Figure S33.2. Forest plot showing the mean difference in AUC_inf_ of raltegravir under fasted and fed conditions (HF – high-fat meal, LF – low-fat meal). **Fixed effects model.**


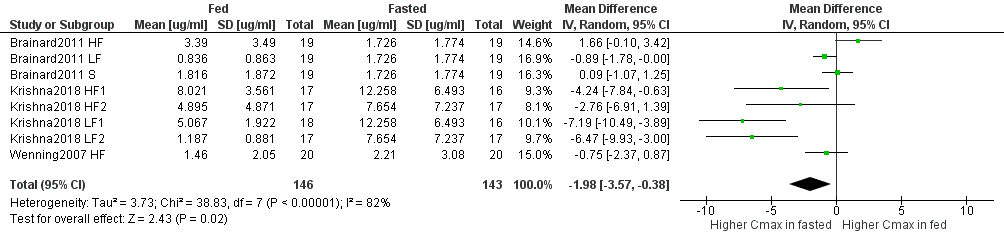


Figure S34. Forest plot showing the mean difference in C_max_ of raltegravir under fasted and fed conditions (HF – high-fat meal, LF – low-fat meal, S – standard meal).


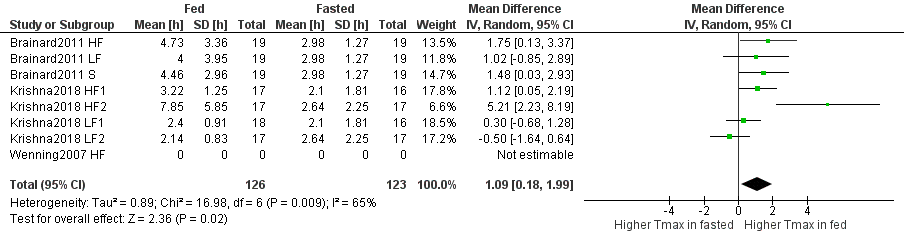


Figure S35. Forest plot showing the mean difference in T_max_ of raltegravir under fasted and fed conditions (HF – high-fat meal, LF – low-fat meal, S – standard meal).

**Amprenavir**


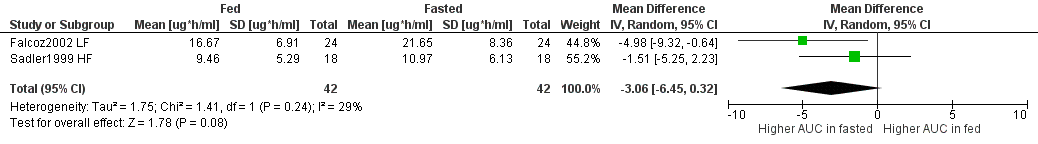


Figure S36. Forest plot showing the mean difference in AUC_inf_ of amprenavir under fasted and fed conditions (LF – low-fat meal, HF – high-fat meal).


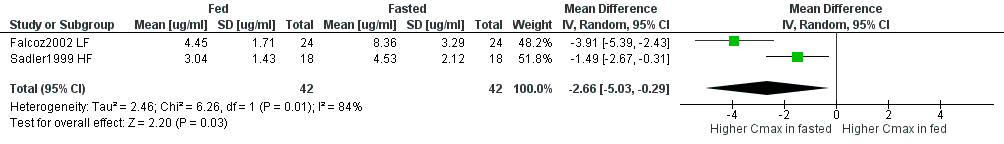


Figure S37. Forest plot showing the mean difference in C_max_ of amprenavir under fasted and fed conditions (LF – low-fat meal, HF – high-fat meal).


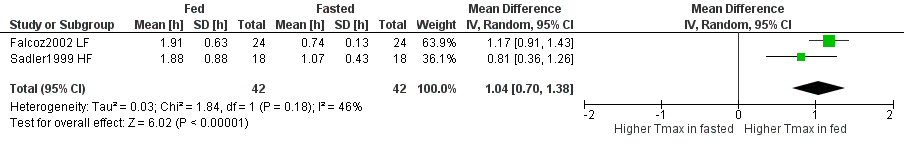


Figure S38. Forest plot showing the mean difference in T_max_ of amprenavir under fasted and fed conditions (LF – low-fat meal, HF – high-fat meal).

**Darunavir**


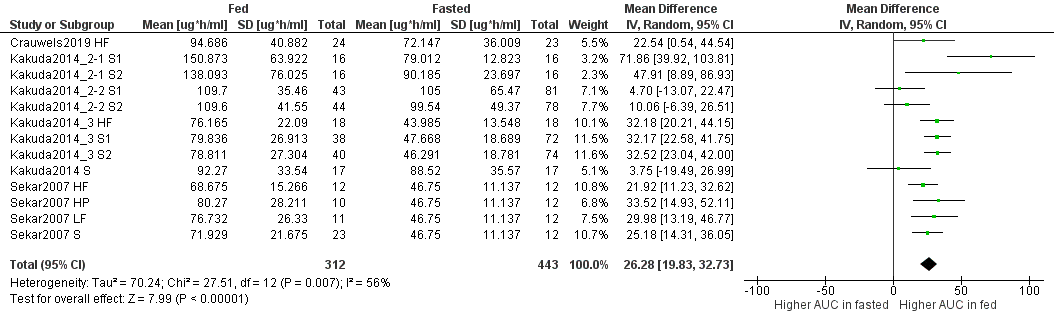


Figure S39. Forest plot showing the mean difference in AUC_inf_ of darunavir under fasted and fed conditions (HF – high-fat meal, S – standard meal, HP – high-protein meal, LF – low-fat meal).


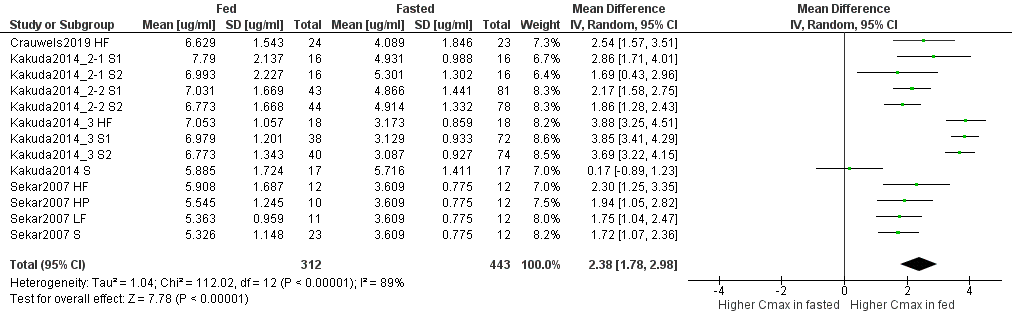


Figure S40. Forest plot showing the mean difference in C_max_ of darunavir under fasted and fed conditions (HF – high-fat meal, S – standard meal, HP – high-protein meal, LF – low-fat meal).


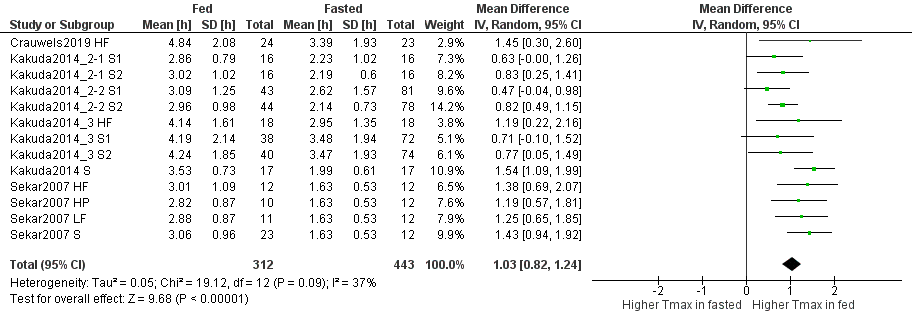


Figure S41. Forest plot showing the mean difference in T_max_ of darunavir under fasted and fed conditions (HF – high-fat meal, S – standard meal, HP – high-protein meal, LF – low-fat meal).

**Indinavir**


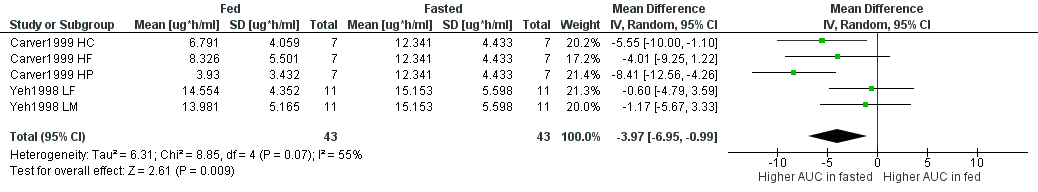


Figure S42. Forest plot showing the mean difference in AUC_inf_ of indinavir under fasted and fed conditions (HC – high-carbohydrate meal, HF – high-fat meal, HP – high-protein meal, LF – low-fat meal, LM – light meal).


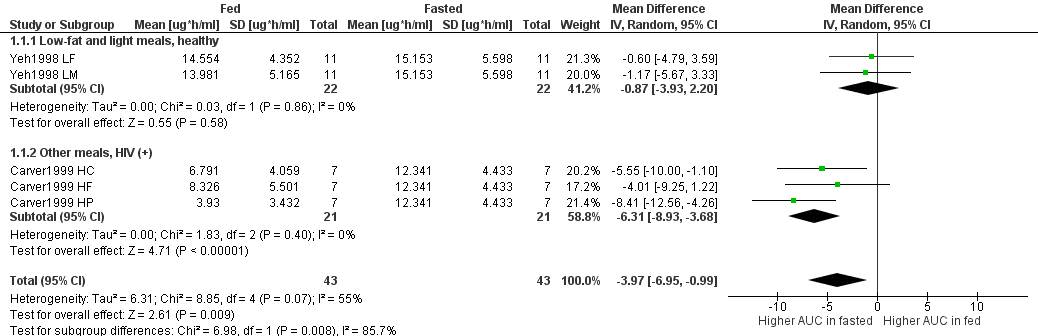


Figure S42.1. Forest plot showing the mean difference in AUC_inf_ of indinavir under fasted and fed conditions by type of meal and participants health state (HC – high-carbohydrate meal, HF – high-fat meal, HP – high-protein meal, LF – low-fat meal, LM – light meal).


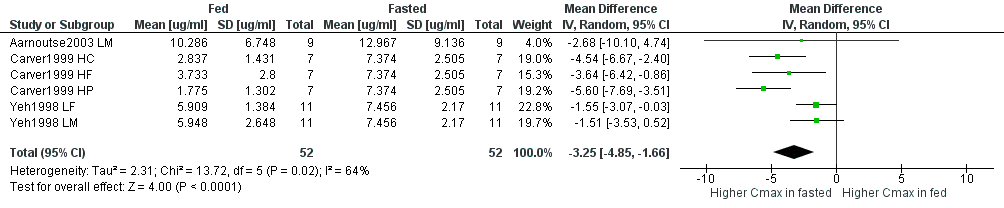


Figure S43. Forest plot showing the mean difference in C_max_ of indinavir under fasted and fed conditions (LM – light meal, HC – high-carbohydrate meal, HF – high-fat meal, HP – high-protein meal, LF – low-fat meal).


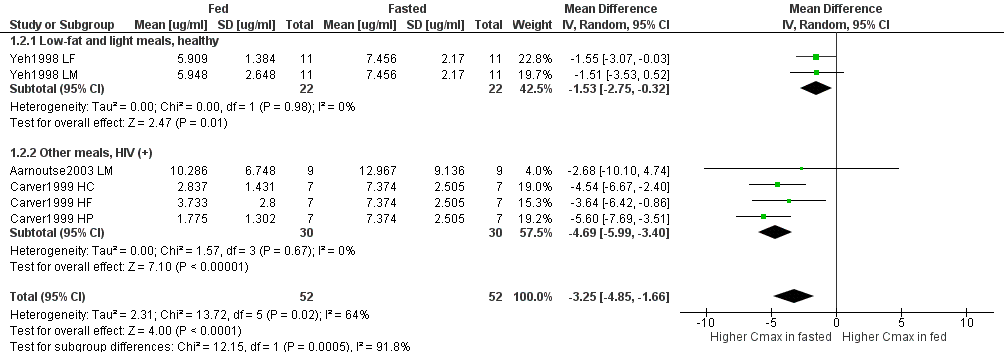


Figure S43.1. Forest plot showing the mean difference in C_max_ of indinavir under fasted and fed conditions by type of meal and participants health state (LM – light meal, HC – high-carbohydrate meal, HF – high-fat meal, HP – high-protein meal, LF – low-fat meal).


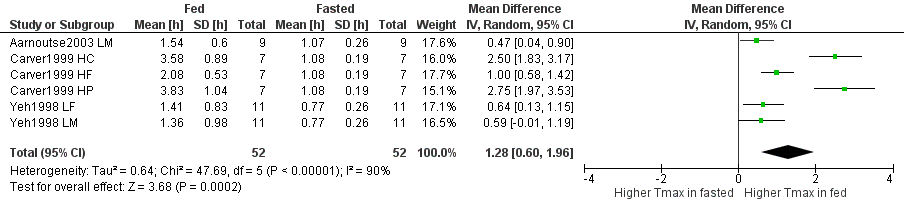


Figure S44. Forest plot showing the mean difference in T_max_ of indinavir under fasted and fed conditions (LM – light meal, HC – high-carbohydrate meal, HF – high-fat meal, HP – high-protein meal, LF – low-fat meal).


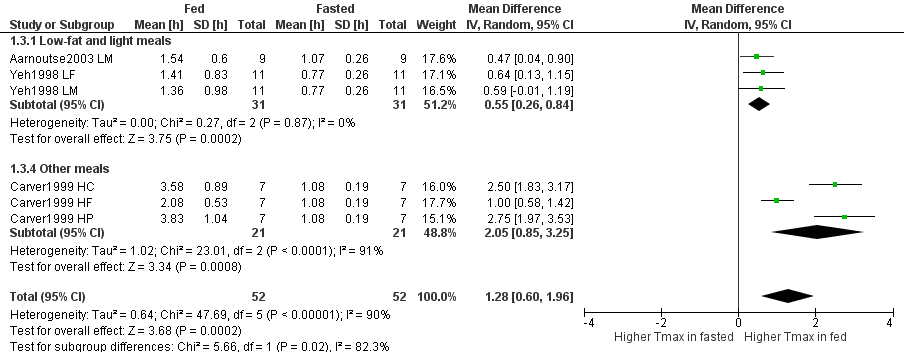


Figure S44.1 Forest plot showing the mean difference in T_max_ of indinavir under fasted and fed conditions by type of meal (LM – light meal, HC – high-carbohydrate meal, HF – high-fat meal, HP – high-protein meal, LF – low-fat meal).


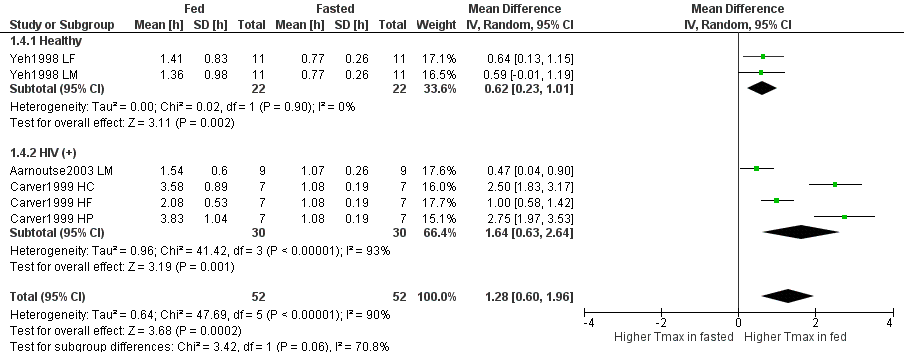


Figure S44.2 Forest plot showing the mean difference in T_max_ of indinavir under fasted and fed conditions by participants health state (LM – light meal, HC – high-carbohydrate meal, HF – high-fat meal, HP – high-protein meal, LF – low-fat meal).

**Lopinavir**


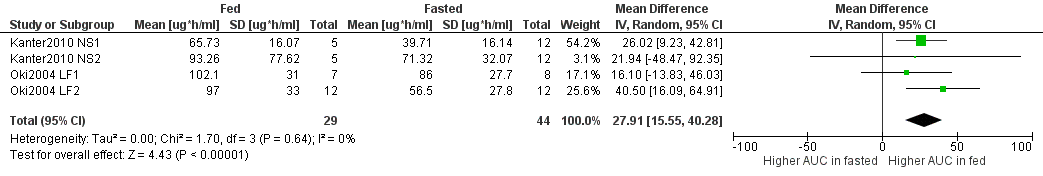


Figure S45. Forest plot showing the mean difference in AUC_inf_ of lopinavir under fasted and fed conditions (NS – not specified, LF – low-fat meal).


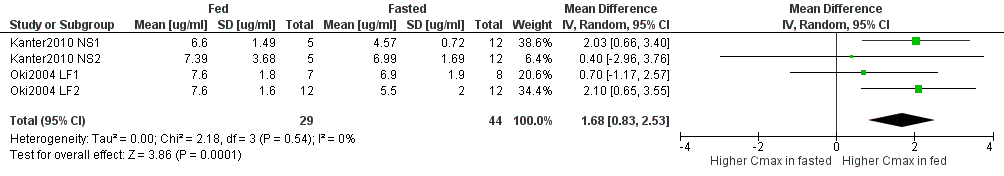


Figure S46. Forest plot showing the mean difference in C_max_ of lopinavir under fasted and fed conditions (NS – not specified, LF – low-fat meal).


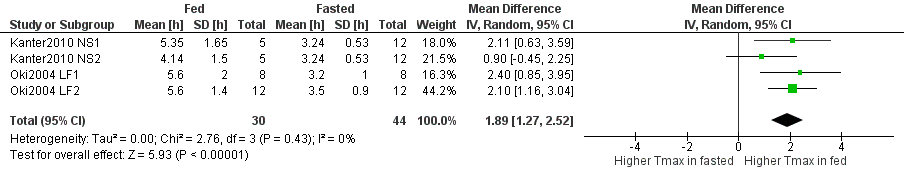


Figure S47. Forest plot showing the mean difference in T_max_ of lopinavir under fasted and fed conditions (NS – not specified, LF – low-fat meal).

**Ritonavir**


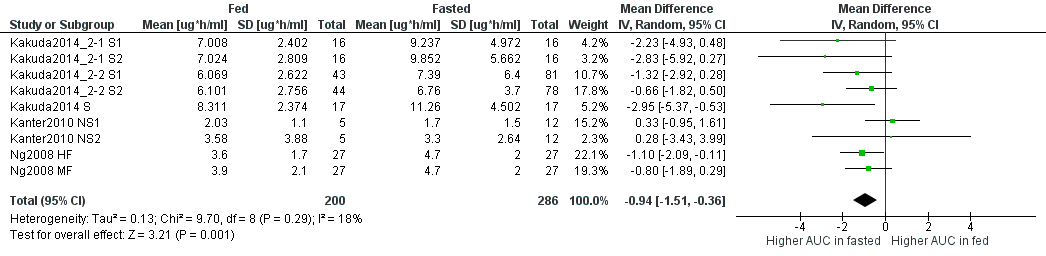


Figure S48. Forest plot showing the mean difference in AUC_inf_ of ritonavir under fasted and fed conditions (S – standard meal, NS – not specified, HF – high-fat meal, MF – moderate-fat meal).


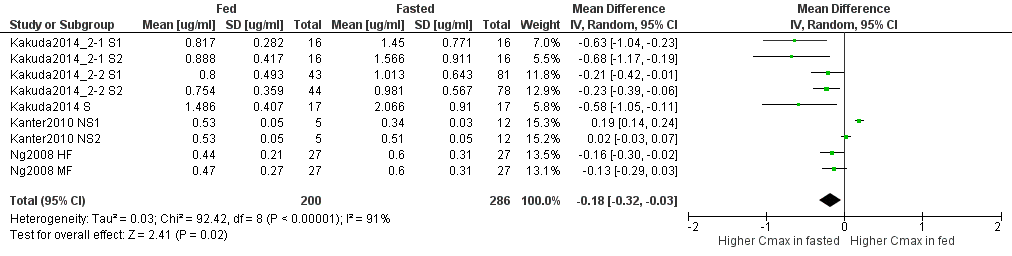


Figure S49. Forest plot showing the mean difference in C_max_ of ritonavir under fasted and fed conditions (S – standard meal, NS – not specified, HF – high-fat meal, MF – moderate-fat meal).


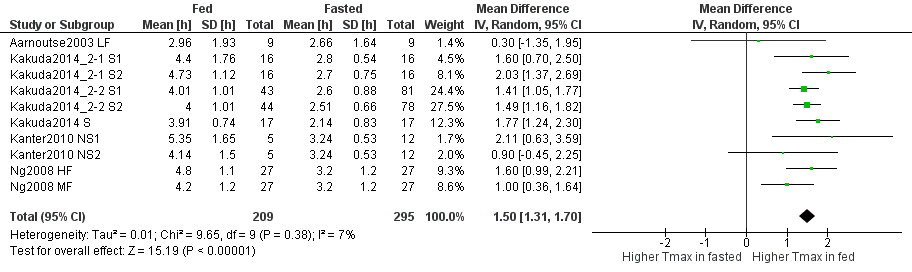


Figure S50. Forest plot showing the mean difference in T_max_ of ritonavir under fasted and fed conditions (LF – low-fat meal, S – standard meal, NS – not specified, HF – high-fat meal, MF – moderate-fat meal).

**References**

1. Chittick GE, Gillotin C, McDowell J a, Lou Y, Edwards KD, Prince WT, et al. Abacavir: absolute bioavailability, bioequivalence of three oral formulations, and effect of food. PHAR Pharmacother J Hum Pharmacol Drug Ther. 1999;19(8):932–42.

2. Yuen GJ, Lou Y, Thompson NF, Otto VR, Allsup TL, Mahony WB, et al. Abacavir/lamivudine/zidovudine as a combined formulation tablet: Bioequivalence compared with each component administered concurrently and the effect of food on absorption. J Clin Pharmacol. 2001;41(3):277–88.

3. Weller S, Chen S, Borland J, Savina P, Wynne B, Piscitelli SC. Bioequivalence of a dolutegravir, abacavir, and lamivudine fixed-dose combination tablet and the effect of food. J Acquir Immune Defic Syndr. 2014;66(4):393–8.

4. Marier JF, Borges M, Plante G, DiMarco M, Morelli G, Tippabhotla SK, et al. Bioequivalence of abacavir generic and innovator formulations under fasting and fed conditions. Int J Clin Pharmacol Ther. 2006;44(6):284–91.

5. Holdich T, Shiveley LA, Sawyer J. Pharmacokinetics of single oral doses of apricitabine, a novel deoxycytidine analogue reverse transcriptase inhibitor, in healthy volunteers. Clin Drug Investig. 2006;26(5):279–86.

6. Knupp CA, Milbrath R, Barbhaiya RH. Effect of Time of Food Administration on the Bioavailability of Didanosine From a Chewable Tablet Formulation. J Clin Pharmacol. 1993;33(6):568–73.

7. Damle BD, Yan JH, Behr D. Effect of food on the oral bioavailability of didanosine from encapsulated enteric-coated beads. J Clin Pharmacol. 2002;42(4):419–27.

8. Hernández-novoa B, Antela A, Gutiérrez C, Pérez-Molina JA, Pérez-Elías MJ, Dronda F, et al. Effect of food on the antiviral activity of didanosine enteric-coated capsules: A pilot comparative study. HIV Med. 2008;9(4):187–91.

9. Shyu WC, Knupp CA, Pittman KA, Dunkle L, Barbhaiya RH. Food-induced reduction in bioavailability of didanosine. Clin Pharmacol Ther. 1991 Nov;50(5/1):503–7.

10. Kearney BP, Sayre JR, Flaherty JF, Chen SS, Kaul S, Cheng AK. Drug-drug and drug-food interactions between tenofovir disoproxil fumarate and didanosine. J Clin Pharmacol. 2005;45(12):1360–7.

11. Stevens RC, Rodman JH, Yong FH, Carey V, Knupp CA, Frenkel LM. Effect of food and pharmacokinetic variability on didanosine systemic exposure in HIV-infected children. Pediatric AIDS Clinical Trials Group Protocol 144 Study Team. AIDS Res Hum Retroviruses. 2000;16(5):415–21.

12. López JC, Moreno S, Jiménez-Oñate F, Clotet B, Rubio R, Hernández-Quero J. A cohort study of the food effect on virological failure and treatment discontinuation in patients on HAART containing didanosine enteric-coated capsules (FOODDle study). HIV Clin Trials. 2006;7(4):155–62.

13. Sánchez-Conde M, Palacios R, Sanz J, Rodríguez-Novoa S, Rivas P, Santos J, et al. Efficacy and safety of a once daily regimen with efavirenz, lamivudine, and didanosine, with and without food, as initial therapy for HIV infection: The ELADI study. AIDS Res Hum Retroviruses. 2007;23(10):1237–41.

14. Wang LH, Gardner P, Frick LW. Pharmacokinetics and safety of 524W91 following single oral administration of escalating doses in HIV-infected volunteers. In: 35th Interscience Conference on Antimicrobial Agents and Chemotherapy. 1995.

15. Crauwels HM, Baugh B, Van Landuyt E, Vanveggel S, Hijzen A, Opsomer M. Bioequivalence of the once-daily single-tablet regimen of darunavir, cobicistat, emtricitabine, and tenofovir alafenamide compared to combined intake of the separate agents and the effect of food on bioavailability. Clin Pharmacol Drug Dev. 2019;8(4):480–91.

16. Bictarvy (Gilead sciences) [Internet]. Prescribing information. [cited 2021 Jul 19]. Available from: https://www.gilead.com/-/media/files/pdfs/medicines/hiv/biktarvy/biktarvy_pi.pdf

17. Han WL, Shang JC, Yan B, Tan R, Huang WX, Zhong XN, et al. Pharmacokinetics of single- and multiple-dose emtricitabine in healthy male chinese volunteers. Pharmacology. 2014;93(3–4):166–71.

18. Majeed SR, German P, West SK, Xiang SS, Costales PJ, Xiao D, et al. Bictegravir / emtricitabine / tenofovir alafenamide low-dose tablet relative bioavailability in healthy volunteers and PK in children with HIV. In: Conference on Retroviruses and Opportunistic Infections, Boston, USA. 2020. p. 3194.

19. Shiomi M, Matsuki S, Ikeda A, Ishikawa T, Nishino N, Kimura M, et al. Effects of a protein-rich drink or a standard meal on the pharmacokinetics of elvitegravir, cobicistat, emtricitabine and tenofovir in healthy Japanese male subjects: A randomized, three-way crossover study. J Clin Pharmacol. 2014;54(6):640–8.

20. Yamada H, Ikushima I, Nemoto T, Ishikawa T, Ninomiya N, Irie S. Effects of a nutritional protein-rich crink on the pharmacokinetics of elvitegravir, cobicistat, emtricitabine, tenofovir alafenamide, and tenofovir compared with a standard meal in healthy Japanese male subjects. Clin Pharmacol Drug Dev. 2018;7(2):132–42.

21. Lamorde M, Byakika-Kibwika P, Tamale WS, Kiweewa F, Ryan M, Amara A, et al. Effect of food on the steady-state pharmacokinetics of tenofovir and emtricitabine plus efavirenz in ugandan adults. AIDS Res Treat. 2012;2012.

22. Custodio JM, Yin X, Hepner M, Ling KHJ, Cheng A, Kearney BP, et al. Effect of food on rilpivirine/emtricitabine/tenofovir disoproxil fumarate, an antiretroviral single-tablet regimen for the treatment of HIV infection. J Clin Pharmacol. 2014;54(4):378–85.

23. Moore KHP. Lamivudine/Zidovudme As a Combined Formulation Tablet: Bioequivalence Compared with lamivudine and zidovudine administered concurrenly and the effect of food on absorption. J Clin Pharmacol. 1999 Mar;39:593–605.

24. Dumitrescu TP, Peddiraju K, Fu C, Bakshi K, Yu S, Zhang Z, et al. Bioequivalence and food effect assessment of 2 fixed-dose combination formulations of dolutegravir and lamivudine. Clin Pharmacol Drug Dev. 2020;9(2):189–202.

25. Behm MO, Yee KL, Liu R, Levine V, Panebianco D, Fackler P. The effect of food on doravirine bioavailability: results from two pharmacokinetic studies in healthy subjects. Clin Drug Investig. 2017;37(6):571–9.

26. Epivir (GlaxoSmithKline) [Internet]. Prescribing information. [cited 2021 Aug 31]. Available from: https://www.accessdata.fda.gov/drugsatfda_docs/label/2017/020564s37_020596s036lbl.pdf

27. Angel JB, Hussey EK, Hall ST, Donn KH, Morris DM, McCormack JP, et al. Pharmacokinetics of 3TC (GR109714X) administered with and without food to HIV-infected patients. Drug Investig. 1993;6(2):70–4.

28. Yee KL, DiBenedetto A, Fan L, Khalilieh S, Triantafyllou I, Vallee MH, et al. Comparative bioavailability of oral granule formulations of the HIV antiretroviral drugs doravirine, lamivudine, and tenofovir disoproxil fumarate. AAPS PharmSciTech. 2020;21(3):1–10.

29. Kaul S, Christofalo B, Raymond RH, Stewart MB, Macleod CM. Effect of food on the bioavailability of stavudine in subjects with human immunodeficiency virus infection. Antimicrob Agents Chemother. 1998;42(9):2295–8.

30. Viread (Gilead sciences) [Internet]. Prescribing information. [cited 2021 Jul 19]. Available from: https://www.ema.europa.eu/en/documents/product-information/viread-epar-product-information_en.pdf

31. Lu C, Jia Y, Chen L, Ding Y, Yang J, Chen M, et al. Pharmacokinetics and food interaction of a novel prodrug of tenofovir, tenofovir dipivoxil fumarate, in healthy volunteers. J Clin Pharm Ther. 2013;38(2):136–40.

32. Custodio JM, Shao Y, Wei X, Vu A, Rhee M, Fordyce M, et al. The effect of food on the pharmacokinetics of unboosted and boosted tenofovir alafenamide. In: 15th European AIDS Conference (EACS). 2015.

33. Li Q, Jia L, Hu W, Dong S, Cai C. Bioequivalence evaluation of two formulations of tenofovir alafenamide tablets in healthy subjects under fasting and fed conditions. Drug Des Devel Ther. 2021;15:2551–62.

34. Li Z, Liu J, Ju G, Yan K, Mao Y, Liu Q, et al. Pharmacokinetics and bioequivalence evaluation of 2 formulations of tenofovir alafenamide. Clin Pharmacol Drug Dev. 2021;10(12):1519–27.

35. Li X, Tan XY, Cui XJ, Yang M, Chen C, Chen XY. Pharmacokinetics of tenofovir alafenamide fumarate and tenofovir in the chinese people: Effects of non-genetic factors and genetic variations. Pharmgenomics Pers Med. 2021;14:1315–29.

36. Nazareno L, Holanzo A, Limjuco R, Passe S. The effect of food on pharmacokinetics of zalcitabine in HIV - positive patients. Pharm Res. 1995;12(10).

37. Unadkat J, Collier A, Crosby SS. Pharmcokinetics of zidovudine (azidothymidine) in patients with AIDS when administered with and without a high-fat meal. AIDS. 1990;4(3):229–32.

38. Lotterer E, Ruhnke M, Trautmann M, Beyer R, Bauer FE. Decreased and variable systemic availability of zidovudine in patients with AIDS if administered with a meal. Eur J Clin Pharmacol. 1991;40(3):305–8.

39. Shelton MJ, Portmore A, Blum MR, Sadler BM, Reichman RC, Morse GD. Prolonged, but not diminished, zidovudine absorption induced by a high‐fat breakfast. Pharmacother J Hum Pharmacol Drug Ther. 1994;14(6):671–7.

40. Sahai J, Gallicano K, Garber G, McGilveray I, Hawley‐Foss N, Turgeon N, et al. The effect of a protein meal on zidovudine pharmacokinetics in HIV‐ infected patients. Br J Clin Pharmacol. 1992;33(6):657–60.

41. Ruhnke M, Bauer FE, Seifert M, Trautmann M, Hille H, Koeppe P. Effects of standard breakfast on pharmacokinetics of oral zidovudine in patients with AIDS. Antimicrob Agents Chemother. 1993;37(10):2153–8.

42. Morse GD, Fischl MA, Shelton MJ, Cox SR, Thompson L, Della-Coletta AA, et al. Effect of food on the steady-state pharmacokinetics of delavirdine in patients with HIV infection. Clin Drug Investig. 2003;23(4):255–61.

43. Anderson MS, Gilmartin J, Cilissen C, De Lepeleire I, Van Bortel L, Dockendorf MF, et al. Safety, tolerability and pharmacokinetics of doravirine, a novel HIV non-nucleoside reverse transcriptase inhibitor, after single and multiple doses in healthy subjects. Antivir Ther. 2015;20(4):397–405.

44. Sustiva (Bristol-Myers Squibb Company) [Internet]. Prescribing information. [cited 2021 Jul 20]. Available from: https://www.ema.europa.eu/en/documents/product-information/sustiva-epar-product-information_en.pdf

45. Kaul S, Ji P, Lu M, Nguyen KL, Shangguan T, Grasela D. Bioavailability in healthy adults of efavirenz capsule contents mixed with a small amount of food. Am J Heal Pharm. 2010;67(3):217–22.

46. Schöller-Gyüre M, Boffito M, Pozniak AL, Leemans R, Kakuda TN, Woodfall B, et al. Effects of different meal compositions and fasted state on the oral bioavailability of etravirine. Pharmacotherapy. 2008;28(10):1215–22.

47. Viramune (Boehringer Ingelheim Pharmaceuticals, Inc.) [Internet]. Prescribing information. [cited 2021 Jul 20]. Available from: https://docs.boehringer-ingelheim.com/Prescribing Information/PIs/Viramune/Viramune.pdf

48. Crauwels HM, Van Heeswijk RPG, Buelens A, Stevens M, Boven K, Hoetelmans RMW. Impact of food and different meal types on the pharmacokinetics of rilpivirine. J Clin Pharmacol. 2013;53(8):834–40.

49. Mehta R, Piscitelli J, Wolstenholme A, Fu C, Crauwels H, Wynne B, et al. The effect of moderate-and high-fat meals on the bioavailability of dolutegravir/rilpivirine fixed-dose combination tablet. Clin Pharmacol Adv Appl. 2020;12:49–52.

50. Crauwels H, Goyvaerts N, Vanveggel S, Solingen R. Relative bioavailability and food effect of a paediatric dispersible tablet formulation of the non-nucleoside reverse transcriptase inhibitor (NNRTI) rilpivirine (RPV). In: International Congress of Drug Therapy in HIV Infection, Glasgow, UK. 2016. p. P032.

51. Lamorde M, Walimbwa S, Byakika-Kibwika P, Katwere M, Mukisa L, Sempa JB, et al. Steady-state pharmacokinetics of rilpivirine under different meal conditions in HIV-1-infected Ugandan adults. J Antimicrob Chemother. 2014;70(5):1482–6.

52. Song I, Borland J, Chen S, Patel P, Wajima T, Peppercorn A, et al. Effect of food on the pharmacokinetics of the integrase inhibitor dolutegravir. Antimicrob Agents Chemother. 2012;56(3):1627–9.

53. Song I, Borland J, Arya N, Wynne B, Piscitelli S. Pharmacokinetics of dolutegravir when administered with mineral supplements in healthy adult subjects. J Clin Pharmacol. 2015;55(5):490–6.

54. Yonemura T, Okada N, Sagane K, Okamiya K, Ozaki H, Iida T, et al. Effects of milk or apple juice ingestion on the pharmacokinetics of elvitegravir and cobicistat in healthy Japanese male volunteers: a randomized, single-dose, three-way crossover study. Clin Pharmacol Drug Dev. 2018;7(7):737–43.

55. Brainard DM, Friedman EJ, Jin B, Breidinger SA, Tillan MD, Wenning LA, et al. Effect of low-, moderate-, and high-fat meals on raltegravir pharmacokinetics. J Clin Pharmacol. 2011;51(3):422–7.

56. Krishna R, Rizk ML, Larson P, Schulz V, Kesisoglou F, Pop R. Single- and Multiple-Dose Pharmacokinetics of Once-Daily Formulations of Raltegravir. Clin Pharmacol Drug Dev. 2018;7(2):196–206.

57. Wenning LA, Anderson MS, Petry AS, Friedman EJ, Kost JT, James S, et al. Raltegravir (RAL) dose proportionality and effect of food. In: 47th Annual ICAAC Chicago, Illinois. 2007. p. A-1046.

58. Rhee EG, Rizk ML, Brainard DM, Gendrano IN, Jin B, Wenning LA, et al. A pharmacokinetic comparison of adult and paediatric formulations of raltegravir in healthy adults. Antivir Ther. 2014;19(6):619–24.

59. Mathias A, Lutz J, West S, Xiao D, Chuck S, Martin H, et al. Pharmacokinetics (PK) of bictegravir (BIC) in combination with polyvalent cation containing (PVCC) antacids and supplements. HIV Med. 2019;20:6.

60. Patel P, Ford SL, Lou Y, Bakshi K, Tenorio AR, Zhang Z, et al. Effect of a high-fat meal on the pharmacokinetics of the HIV integrase inhibitor cabotegravir. Clin Pharmacol Drug Dev. 2019;8(4):443–8.

61. Patel P, Ford SL, Lou Y, Bakshi K, Tenorio AR, Zhang Z, et al. Single dose randomized study to assess the relative bioavailability of two formulations and food effect of cabotegravir in healthy male and female subjects. Unpubl study. 2018;

62. Patel P, Ford SL, Lou Y, Bakshi K, Tenorio AR, Zhang Z, et al. A two part, single-center, randomized, open-label, crossover study to assess the relative bioavailability of new tablet formulations of GSK1265744 in healthy adult subjects. Unpubl study. 2018;

63. Agenerase (Glaxo Group Ltd) [Internet]. Product characteristics. [cited 2021 Jul 21]. Available from: https://ec.europa.eu/health/documents/community-register/2000/200010203852/anx_3852_en.pdf

64. Sadler BM, Hanson CD, Chittick GE, Symonds WT, Roskell NS. Safety and pharmacokinetics of amprenavir (141W94), a human immunodeficiency virus (HIV) type 1 protease inhibitor, following oral administration of single doses to HIV-infected adults. Antimicrob Agents Chemother. 1999;43(7):1686–92.

65. Brouwers J, Tack J, Augustijns P. Parallel monitoring of plasma and intraluminal drug concentrations in man after oral administration of fosamprenavir in the fasted and fed state. Pharm Res. 2007;24(10):1862–9.

66. Falcoz C, Jenkins JM, Bye C, Hardman TC, Kenney KB, Studenberg S, et al. Pharmacokinetics of GW433908, a prodrug of amprenavir, in healthy male volunteers. J Clin Pharmacol. 2002;42(8):887–98.

67. Reyataz (Bristol-Myers Squibb Company) [Internet]. Prescribing information. [cited 2021 Jul 21]. Available from: https://www.accessdata.fda.gov/drugsatfda_docs/label/2016/021567s039,206352s004lbl.pdf

68. Sevinsky H, Tao X, Wang R, Ravindran P, Sims K, Xu X, et al. A randomized trial in healthy subjects to assess the bioequivalence of an atazanavir/cobicistat fixed-dose combination tablet versus administration as separate agents. Antivir Ther. 2015;20(5):493–500.

69. Sekar V, Kestens D, Spinosa-Guzman S, De Pauw M, De Paepe E, Vangeneugden T, et al. The effect of different meal types on the pharmacokinetics of darunavir (TMC114)/ritonavir in HIV-negative healthy volunteers. J Clin Pharmacol. 2007;47(4):479–84.

70. Kakuda TN, De Casteele T Van, Petrovic R, Neujens M, Salih H, Opsomer M, et al. Bioequivalence of a darunavir/cobicistat fixed-dose combination tablet versus single agents and food effect in healthy volunteers. Antivir Ther. 2014;19(6):597–606.

71. Kakuda TN, Leopold L, Timmers M, Van De Casteele T, Hillewaert V, Tomaka FL, et al. Bioavailability and bioequivalence of a darunavir 800-mg tablet formulation compared with the 400-mg tablet formulation. Int J Clin Pharmacol Ther. 2014;52(9):805–16.

72. Kakuda TN, Sekar V, Lavreys L, de Paepe E, Stevens T, Vanstockem M, et al. Pharmacokinetics of darunavir after administration of an oral suspension with low-dose ritonavir and with or without food. Clin Pharmacol Drug Dev. 2014;3(5):346–52.

73. Cloarec N, Solas C, Ladaique A, Tamalet C, Zaegel-Faucher O, Bregigeon S, et al. Sub-therapeutic darunavir concentration and garlic consumption; a “Mediterranean” drug-food interaction, about 2 cases. Eur J Clin Pharmacol. 2017;73(10):1331–3.

74. Lexiva (GlaxoSmithKline) [Internet]. Prescribing information. [cited 2021 Sep 8]. Available from: https://www.accessdata.fda.gov/drugsatfda_docs/label/2009/021548s021,022116s005lbl.pdf

75. Yeh KC, Deutsch PJ, Haddix H, Hesney M, Hoagland V, Ju WD, et al. Single-dose pharmacokinetics of indinavir and the effect of food. Antimicrobal Agents Chemother. 1998;42(2):332–8.

76. Carver PL, Fleisher D, Zhou SY, Kaul D, Kazanjian P, Cheng L. Meal composition effects on the oral bioavailability of indinavir in HIV-infected patients. Vol. 16, Pharmaceutical Research. 1999. p. 718–24.

77. Aarnoutse RE, Wasmuth JC, Fätkenheuer G, Schneider K, Schmitz K, De Boo TM, et al. Administration of indinavir and low-dose ritonavir (800/100 mg twice daily) with food reduces nephrotoxic peak plasma levels of indinavir. Antivir Ther. 2003;8(4):309–14.

78. Saah AJ, Winchell GA, Nessly ML, Seniuk MA, Rhodes RR, Deutsch PJ. Pharmacokinetic profile and tolerability of indinavir-ritonavir combinations in healthy volunteers. Antimicrob Agents Chemother. 2001;45(10):2710–5.

79. Kaletra (Abbott) [Internet]. Prescribing information. [cited 2021 Jul 21]. Available from: https://www.accessdata.fda.gov/drugsatfda_docs/label/2013/021251s046_021906s039lbl.pdf

80. Klein CE, Chiu YL, Awni W, Zhu T, Heuser RS, Doan T, et al. The tablet formulation of lopinavir/ritonavir provides similar bioavailability to the soft-gelatin capsule formulation with less pharmacokinetic variability and diminished food effect. J Acquir Immune Defic Syndr. 2007;44(4):401–10.

81. Lamorde M, Byakika-Kibwika P, Boffito M, Nabukeera L, Mayito J, Ogwal-Okeng J, et al. Steady-state pharmacokinetics of lopinavir plus ritonavir when administered under different meal conditions in HIV-infected ugandan adults. J Acquir Immune Defic Syndr [Internet]. 2012;60(3):295–8. Available from: http://ovidsp.ovid.com/ovidweb.cgi?T=JS&PAGE=reference&D=emed10&NEWS=N&AN=2012439605

82. Oki T, Usami Y, Nakai M, Sagisaka M, Ito H, Nagaoka K, et al. Pharmacokinetics of lopinavir after administration of Kaletra in healthy Japanese volunteers. Biol Pharm Bull. 2004;27(2):261–5.

83. Kanter CTMM, Colbers EPH, Fillekes Q, Hoitsma A, Burger DM. Pharmacokinetics of two generic co-formulations of lopinavir/ritonavir for HIV-infected children: A pilot study of paediatric Lopimune versus the branded product in healthy adult volunteers. J Antimicrob Chemother. 2010;65(3):538–42.

84. Viracept (ViiV Healthcare Company) [Internet]. Prescribing information. [cited 2021 Jul 21]. Available from: https://www.accessdata.fda.gov/drugsatfda_docs/label/2012/020778s036,020779s057,021503s018lbl.pdf

85. Kaeser B, Charoin JE, Gerber M, Oxley P, Birnboeck H, Saiedabadi N, et al. Assessment of the bioequivalence of two nelfinavir tablet formulations under fed and fasted conditions in healthy subjects. Int J Clin Pharmacol Ther. 2005;43(3):154–62.

86. Aarnoutse RE, Droste JAH, Van Oosterhout JJG, Koopmans PP, Popescu M, Reiss P, et al. Pharmacokinetics, food intake requirements and tolerability of once-daily combinations of nelfinavir and low-dose ritonavir in healthy volunteers. Br J Clin Pharmacol. 2003;55(2):115–25.

87. Kurowski M, Kaeser B, Sawyer A, Popescu M, Mroziekiewicz A, Arslan A, et al. Limited effect of food composition on the pharmacokinetics of nelfinavir administered twice daily. Eur J Med Res. 2002;7(10):453–6.

88. Ng J, Klein C, Chui Y, Awni W, Morris J, Podsadecki T, et al. The effect of food on ritonavir bioavailability following administration of ritonavir 100 mg film-coated tablet in healthy adult subjects. J Int AIDS Soc. 2008;11(Suppl 1):P247.

89. Norvir (AbbVie Inc.) [Internet]. Prescribing information. [cited 2021 Jul 21]. Available from: https://www.accessdata.fda.gov/drugsatfda_docs/label/2013/020659s058_022417s010lbl.pdf

90. Salem AH, Chiu YL, Valdes JM, Nilius AM, Klein CE. A novel ritonavir paediatric powder formulation is bioequivalent to ritonavir oral solution with a similar food effect. Antivir Ther. 2015;20(4):425–32.

91. Veldkamp AI, van Heeswijk RPG, Mulder JW, Meenhorst PL, Schreij G, van der Geest S, et al. Steady-state pharmacokinetics of twice-daily dosing of saquinavir plus ritonavir in HIV-1–infected individuals. JAIDS J Acquir Immune Defic Syndr. 2001 Aug;27(4):344–9.

92. Gallicano K, Foster B, Choudhri S. Effect of short-term administration of garlic supplements on single- dose ritonavir pharmacokinetics in healthy volunteers. Br J Clin Pharmacol. 2003;199–202.

93. Invirase (Roche Pharma) [Internet]. Prescribing information. [cited 2021 Aug 31]. Available from: https://www.accessdata.fda.gov/drugsatfda_docs/label/2012/020628s034-021785s011lbl.pdf

94. Kenyon CJ, Brown F, McClelland GR, Wilding IR. The use of pharmacoscintigraphy to elucidate food effects observed with a novel protease inhibitor (saquinavir). Pharm Res. 1998;15(3):417–22.

95. Hugen PWH, Burger DM, Koopmans PP, Cohen Stuart JWT, Kroon FP, Van Leusen R, et al. Saquinavir soft-gel capsules (Fortovase®) give lower exposure than expected, even after a high-fat breakfast. Pharm World Sci. 2002;24(3):83–6.

96. Piscitelli SC, Burstein AH, Welden N, Gallicano KD, Falloon J. The Effect of Garlic Supplements on the Pharmacokinetics of Saquinavir. Clin Infect Dis. 2002;34(2):234–8.

97. Aptivus (Boehringer Ingelheim Pharmaceuticals, Inc.) [Internet]. Prescribing information. [cited 2021 Jul 21]. Available from: https://www.accessdata.fda.gov/drugsatfda_docs/label/2011/021814s011lbl.pdf

98. Selzentry (Pfeizer Labs) [Internet]. Prescribing information. [cited 2021 Aug 18]. Available from: https://www.accessdata.fda.gov/drugsatfda_docs/label/2007/022128lbl.pdf#:~:text=SELZENTRY is a CCR5 co-receptor antagonist indicated for,history should guide the use of SELZENTRY %281%29.

99. Fätkenheuer G, Pozniak AL, Johnson MA, Plettenberg A, Staszewski S, Hoepelman AIM, et al. Efficacy of short-term monotherapy with maraviroc, a new CCR5 antagonist, in patients infected with HIV-1. Nat Med. 2005;11(11):1170–2.

100. Rukobia (ViiV Healthcare BV) [Internet]. Product characteristics. [cited 2021 Aug 18]. Available from: https://www.ema.europa.eu/en/documents/product-information/rukobia-epar-product-information_en.pdf

101. Shelton MJ, Hewitt RG, Adams JM, Cox SR, Chambers JH, Morse GD. Delavirdine malabsorption in HIV-infected subjects with spontaneous gastric hypoacidity. J Clin Pharmacol. 2003;43(2):171–9.

102. Demarles D, Gillotin C, Bonaventure-Paci S, Vincent I, Fosse S, Taburet AM. Single-dose pharmacokinetics of amprenavir coadministered with grapefruit juice. Antimicrob Agents Chemother. 2002;46(5):1589–90.

103. Shelton MJ, Wynn HE, Hewitt RG, DiFrancesco R. Effects of grapefruit juice on pharmacokinetic exposure to indinavir in HIV-positive subjects. J Clin Pharmacol. 2001;41(4):435–42.

104. Penzak SR, Acosta EP, Turner M, Edwards DJ, Hon YY, Desai HD, et al. Effect of Seville orange juice and grapefruit juice on indinavir pharmacokinetics. J Clin Pharmacol. 2002;42(10):1165–70.

105. Kupferschmidt HHT, Fattinger KE, Ha HR, Follath F, Krähenbühl S. Grapefruit juice enhances the bioavailability of the HIV protease inhibitor saquinavir in man. Br J Clin Pharmacol. 1998;45(4):355–9.

106. Gruber VA, Rainey PM, Lum PJ, Beatty GW, Aweeka FT, McCance-Katz EF. Interactions between alcohol and the HIV entry inhibitor maraviroc. J Int Assoc Provid AIDS Care. 2013;12(6):375–7.

107. McDowell JA, Chittick GE, Stevens C, Edwards KD, Stein DS. Pharmacokinetic interaction of abacavir ( 1592U89 ) and ethanol in Human Immunodeficiency Virus-infected adults. Antimicrobal Agents Chemother. 2000;44(6):1686–90.

108. McCance-Katz EF, Gruber VA, Beatty G, Lum PJ, Rainey PM. Interactions between alcohol and the antiretroviral medications ritonavir or efavirenz. J Addict Med. 2013;7(4):264–70.
